# Effect of exercise on cardiometabolic health of adults with overweight or obesity: Focus on blood pressure, insulin resistance, and intrahepatic fat—A systematic review and meta‐analysis

**DOI:** 10.1111/obr.13269

**Published:** 2021-05-06

**Authors:** Francesca Battista, Andrea Ermolao, Marleen A. van Baak, Kristine Beaulieu, John E. Blundell, Luca Busetto, Eliana V. Carraça, Jorge Encantado, Dror Dicker, Nathalie Farpour‐Lambert, Adriyan Pramono, Alice Bellicha, Jean‐Michel Oppert

**Affiliations:** ^1^ Sport and Exercise Medicine Division, Department of Medicine University of Padova Padua Italy; ^2^ NUTRIM School of Nutrition and Translational Research in Metabolism, Department of Human Biology Maastricht University Maastricht The Netherlands; ^3^ Appetite Control and Energy Balance Research Group (ACEB), School of Psychology, Faculty of Medicine and Health University of Leeds Leeds UK; ^4^ Obesity Management Task Force (OMTF) European Association for the Study of obesity (EASO); ^5^ Department of Medicine University of Padova Padua Italy; ^6^ Faculdade de Educação Física e Desporto, CIDEFES Universidade Lusófona de Humanidades e Tecnologias Lisbon Portugal; ^7^ APPsyCI – Applied Psychology Research Center Capabilities & Inclusion ISPA ‐ University Institute Lisbon Portugal; ^8^ Department of Internal Medicine D, Hasharon Hospital, Rabin Medical Center, Sackler School of Medicine Tel Aviv University Tel Aviv Israel; ^9^ Obesity Prevention and Care Program Contrepoids, Service of Endocrinology, Diabetology, Nutrition and Patient Education, Department of Internal Medicine University Hospitals of Geneva and University of Geneva Geneva Switzerland; ^10^ INSERM, Nutrition and Obesities: Systemic Approaches (NutriOmics) Sorbonne University Paris France; ^11^ UFR SESS‐STAPS University Paris‐Est Créteil Créteil France; ^12^ Assistance Publique‐Hôpitaux de Paris (AP‐HP), Pitié‐Salpêtrière hospital, Department of Nutrition, Institute of Cardiometabolism and Nutrition Sorbonne University Paris France

**Keywords:** hypertension, insulin resistance, morbid obesity, NAFLD, physical activity, type 2 diabetes

## Abstract

This systematic review examined the impact of exercise intervention programs on selected cardiometabolic health indicators in adults with overweight or obesity. Three electronic databases were explored for randomized controlled trials (RCTs) that included adults with overweight or obesity and provided exercise‐training interventions. Effects on blood pressure, insulin resistance (homeostasis model of insulin resistance, HOMA‐IR), and magnetic resonance measures of intrahepatic fat in exercise versus control groups were analyzed using random effects meta‐analyses. Fifty‐four articles matched inclusion criteria. Exercise training reduced systolic and diastolic blood pressure (mean difference, MD = −2.95 mmHg [95% CI −4.22, −1.68], *p* < 0.00001, *I* ^2^ = 63% and MD = −1.93 mmHg [95% CI −2.73, −1.13], *p* < 0.00001, *I* ^2^ = 54%, 60 and 58 study arms, respectively). Systolic and diastolic blood pressure decreased also when considering only subjects with hypertension. Exercise training significantly decreased HOMA‐IR (standardized mean difference, SMD = −0.34 [−0.49, −0.18], *p* < 0.0001, *I* ^2^ = 48%, 37 study arms), with higher effect size in subgroup of patients with type 2 diabetes (SMD = −0.50 [95% CI: −0.83, −0.17], *p* = 0.003, *I* ^2^ = 39%). Intrahepatic fat decreased significantly after exercise interventions (SMD = −0.59 [95% CI: −0.78, −0.41], *p* < 0.00001, *I* ^2^ = 0%), with a larger effect size after high‐intensity interval training. In conclusion, exercise training is effective in improving cardiometabolic health in adults with overweight or obesity also when living with comorbitidies.

## 1 INTRODUCTION

Obesity is a global and growing multifactorial disease as well as a public health issue.^1^ The pandemic expansion of obesity is accompanied by the concomitant growth of many related comorbidities, themselves determinants of increased cardiovascular risk.^2^ Indeed, increased blood glucose, high blood pressure, altered lipid profile, and increased intrahepatic fat are all cardiometabolic risk markers closely related to obesity, with insulin resistance as a common pathophysiological pathway.^3^ All international guidelines recommend to prescribe physical exercise for the management of type 2 diabetes,^4^ arterial hypertension,^5^ and obesity‐related comorbidities such as nonalcoholic fatty liver disease (NAFLD).^6^ However, the effect of exercise training on these aspects of cardiometabolic health have not been specifically addressed in subjects with overweight or obesity. In 2006, a Cochrane review based on a very limited number of randomized controlled trials (RCTs) available showed that groups of adults with overweight or obesity participating in physical exercise intervention programs experienced advantages for cardiometabolic health (i.e., blood pressure lowering and improved lipid profile), particularly if involved in higher‐intensity exercise training.^7^ In 2013, Pattyn et al.,^8^ focusing on subjects with metabolic syndrome, displayed the efficacy of aerobic training in improving cardiovascular risk factors. In 2012, Cornelissen et al.^9^ described the significant effect of resistance training in reducing systolic and diastolic blood pressure, but this meta‐analysis did not address individuals with overweight or obesity. Moreover, previous systematic reviews and meta‐analysis outlined significant effect of regular exercise in improving insulin sensitivity in patients with type 2 diabetes, but limited data are available in patients with overweight and obesity with or without type 2 diabetes.^10^, ^11^

High‐intensity interval training (HIIT) is currently receiving increasing attention regarding its metabolic and cardiovascular effects. Recently, Batacan et al.^12^ highlighted the beneficial effect of HIIT on cardiometabolic health in subjects with overweight or obesity. On the other hand, also high intensity continuous training showed powerful effect in reducing intrahepatic fat, a main marker of metabolic dysfunction in people with obesity.^13^ NAFLD is currently recognized as a major health concern, considering that it can give rise to serious forms of liver disease such as cirrhosis and hepatic carcinoma.^14^ Prior reviews showed that exercise training alone is effective in reducing different measures of intrahepatic fat in patients with NAFLD,^13^, ^15^, ^16^ particularly for higher energy expenditure programmes.^17^

In summary, a specific focus on overweight or obesity is lacking in most previous reviews and meta‐analyses. Therefore, given the growing number of individuals affected by obesity‐related comorbidities^18^, ^19^, ^20^, ^21^ and the central role of physical exercise in their management,^22^ it seemed suitable to perform an update and synthesis about the effect of exercise training programs on cardiometabolic health parameters in adults with overweight or obesity. The main objective of this work is to evaluate through systematic review and meta‐analysis the effect of exercise training on blood pressure values, insulin resistance, and magnetic resonance (MR) measures of intrahepatic fat in patients with overweight or obesity with or without obesity‐related comorbidities.

## 2 METHODS

This systematic review follows the Preferred Reporting Items for Systematic Reviews and Meta‐Analysis (PRISMA) guidelines and is registered in the PROSPERO database (registration number CRD42019157823).

### 2.1 Search strategy

Three electronic databases (PubMed, Web of Science, and Cochrane Library) were searched for original articles published up to April 2020 using the strategy “obesity AND physical activity AND age AND comorbidities.” Previous systematic reviews were screened to identify relevant subject headings and keywords to include within each subject category. The specific keywords used for the search are listed in Table S1. Limits were set to include RCTs, only with adults, and that were published in English. Reference lists from the resulting articles were also screened to identify additional articles.

### 2.2 Study selection, inclusion, and exclusion

RCTs were included if they involved adults (≥18 years including older adults) with overweight (body mass index [BMI] ≥ 25 kg/m^2^) or obesity (BMI ≥ 30 kg/m^2^) participating in physical activity interventions, that is, exercise training programs. Original articles focusing on the primary prevention of weight gain/obesity were not included. Presence of obesity comorbidities was not an exclusion criterion, provided that the article focused on subjects with obesity. Specifically, subjects with the following comorbidities were not excluded: type 2 diabetes, hypertension, dyslipidaemia, metabolic syndrome, NAFLD, and nonalcoholic steatohepatitis (NASH). No minimum intervention length criterion was applied. Exercise training programs included sessions with one or more types of exercise (aerobic and/or resistance and/or HIIT). Exercise sessions could have been fully supervised, partially supervised or nonsupervised. Physical activity programs in combination with other interventions (e.g., diet, dietary supplements, or drugs) with appropriate controls were included. Comparators included no intervention or usual care (i.e., intervention that any patient would have received in the framework of obesity management).

Abstracts and full texts were independently assessed for eligibility by two authors (FB and AE) with uncertainty regarding eligibility discussed among authors.

### 2.3 Data extraction and synthesis

Data were extracted by two authors (FB and AE) using standardized forms. Characteristics of each included original article were recorded: reference, study design, participants included in intervention and control groups, population characteristics (age, BMI, % female, and comorbidities for intervention and control groups), description of intervention (program duration, number of sessions/week, type of training/modality, and supervision/delivery), and comparison, outcomes, and duration of follow‐up.

The findings pertaining to systolic blood pressure, diastolic blood pressure, insulin resistance, and intrahepatic fat (measured only by MR) of each included original article are reported. In addition, the study authors' conclusions were extracted, and an overview of the quality of the original studies and current authors' assessment of conclusions is provided for each included article.

Effects on systolic and diastolic blood pressure, Homeostasis Model Assessment Insulin Resistance Index (HOMA‐IR), and intrahepatic fat were examined using random effects meta‐analyses (Review Manager Version 5.3). Analyses are presented as mean difference (MD) for systolic and diastolic blood pressure and as standardized mean difference (SMD) for HOMA‐IR and intrahepatic fat. Sensitivity analysis by using one‐study‐removed procedure was also conducted. Effect sizes were considered large, medium, small, and negligible when SMD was >0.8, between 0.5 and 0.8, between 0.2 and 0.5, and below 0.2, respectively. Heterogeneity was calculated using the *I* ^2^ test. Heterogeneity was considered low, moderate, and high when *I* ^2^ was <50%, between 50% and 75%, and ≥75%, respectively.^23^ Publication bias was assessed with visual inspection of the funnel plot. Limit for statistical significance was fixed at a *p* value of 0.05. Absolute change (pre‐ to postintervention values) in intervention and control groups was indicated as mean and standard deviation (*SD*). For missing data, authors were contacted. Alternatively, conversion of confidence intervals in *SD* or calculation of *SD* of absolute change (when studies provided the exact *p* value for intragroup or intergroup analyses) was performed by formulas and transformation methods of the Cochrane handbook.^24^ When these values were not reported, we calculated the MD as the difference in mean pre‐ and postintervention and its *SD* using the formula: *SD* = square root ([*SD*_pretreatment_]^2^ + [*SD*_posttreatment_]^2^) – [2*r* × *SD*_pretreatment_ × *SD*_posttreatment_]). Because the pretest–posttest correlation coefficients (*r*) were not reported in the studies, a conservative *r* value of 0.5 was assumed throughout. Data from figures were extracted using an online tool (WebPlotDigitizer; https://automeris.io/WebPlotDigitizer/). If a study included more than two experimental groups, which were compared with one control group, the number of subjects in the control group was divided by the number of included intervention arms.

### 2.4 Quality assessment

Study quality was assessed with a standardized tool including 14 criteria, as previously described.^25^ Study quality was defined as good, fair, and poor when 0, 1, or ≥2 criteria considered as “fatal flaws” were not fulfilled.^25^ Three assessment items represented fatal flaws if answered “No/Not reported/Can't determine”: (i) randomization (#1), (ii) dropout rate <20% (#7), and (iii) intent‐to‐treat analysis (#14). Quality assessment was conducted independently by two reviewers (FB ad AE) using this standardized tool. Any disagreement between the reviewers was resolved through discussion.

## 3 RESULTS

**FIGURE 1:**
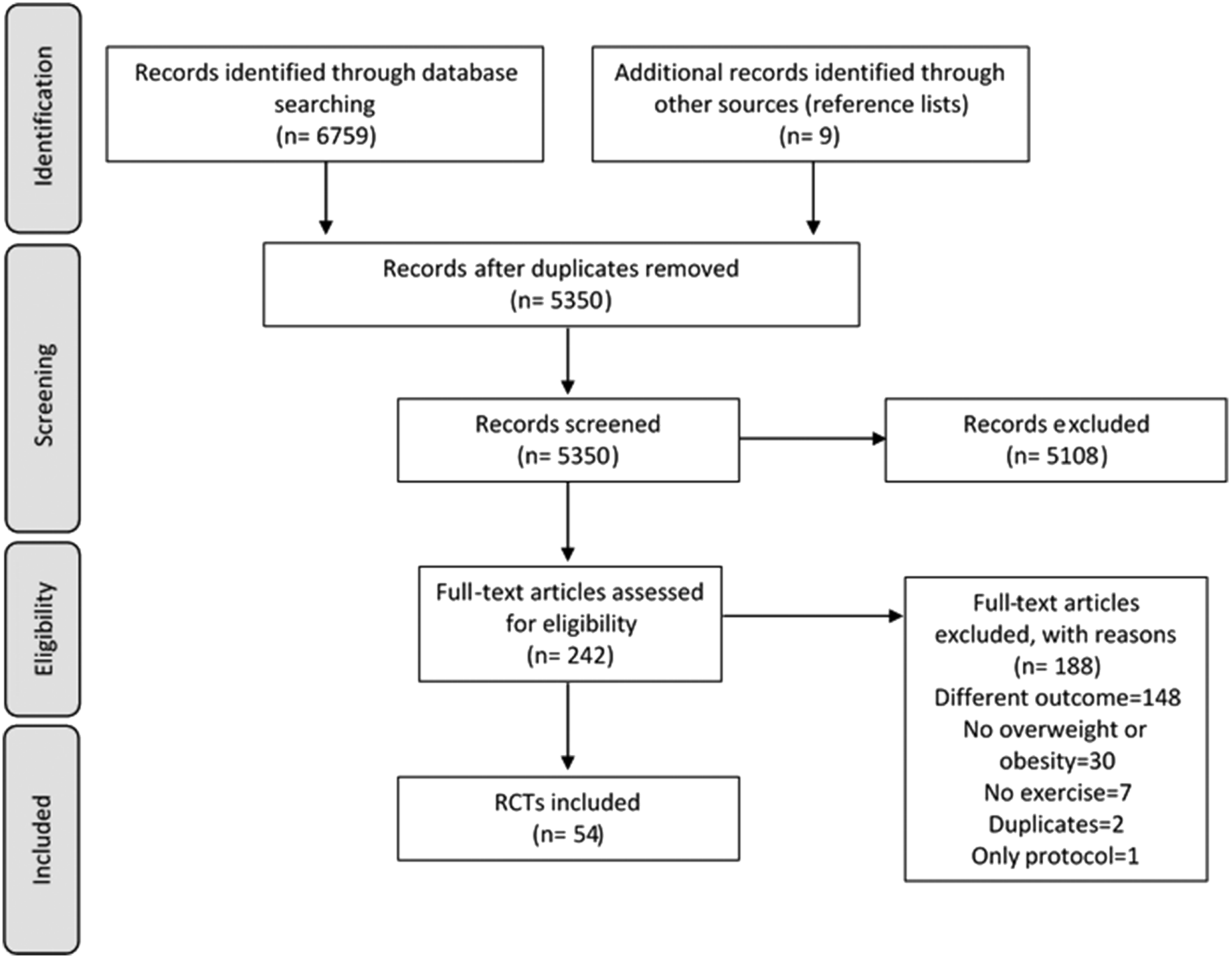
Preferred Reporting Items for Systematic Reviews and Meta‐Analysis (PRISMA) flow diagram

The systematic review flow diagram is presented in Figure 1. The comprehensive (databases and reference lists) search yielded 6768 articles; 1418 were removed because they were duplicates. Of the remaining 5350, 5108 were eliminated based on titles and abstracts alone. The full text was retrieved from 242 articles, and 54 satisfied the inclusion criteria.

### 3.1 Study characteristics

Characteristics and findings of the included original studies are presented in Tables S3–S4. RCTs were published between 2006 and April 2020. Fifty‐four RCTs were included. The median (range) total sample size of the included studies was 38 (10–404) subjects. The median (range) age was 52 (22–70) years. Fifty‐three studies reported BMI at baseline, with the median (range) being 31.9 (26.3–43.7) kg/m^2^. In the single study^26^ not reporting BMI values, the presence of obesity (BMI ≥ 30 kg/m^2^) was an inclusion criteria. Males and females were present in 34 studies^18^, ^21^, ^26^, ^27^, ^28^, ^29^, ^30^, ^31^, ^32^, ^33^, ^34^, ^35^, ^36^, ^37^, ^38^, ^39^, ^40^, ^41^, ^42^, ^43^, ^44^, ^45^, ^46^, ^47^, ^48^, ^49^, ^50^, ^51^, ^52^, ^53^, ^54^, ^55^, ^56^, ^57^ in which the median (range) percentage of females was 59.0 (15.9–92.1)%. Eight studies included only male,^58^, ^59^, ^60^, ^61^, ^62^, ^63^, ^64^, ^65^ and 12 studies included only female adults.^66^, ^67^, ^68^, ^69^, ^70^, ^71^, ^72^, ^73^, ^74^, ^75^, ^76^, ^77^ Five studies included subjects with several comorbidities including arterial hypertension, type 2 diabetes, and metabolic syndrome.^27^, ^43^, ^46^, ^47^, ^53^ Six studies included patients with hypertension and metabolic syndrome.^26^, ^36^, ^44^, ^54^, ^57^, ^68^ Four studies were performed on subjects affected by hypertension and NAFLD,^41^, ^51^, ^55^, ^78^ three studies included subjects affected by NAFLD and type 2 diabetes,^28^, ^29^, ^50^ and two included also patients with dyslipidaemia.^18^, ^52^ Two studies enrolled patients with only type 2 diabetes,^49^, ^58^ 12 studies with only hypertension,^30^, ^31^, ^33^, ^39^, ^42^, ^48^, ^62^, ^63^, ^64^, ^66^, ^71^, ^75^ one with NAFLD,^45^ and one with NASH.^32^ Interventions are described in detail in Table S3. Duration of exercise training ranged from 4 to 48 weeks, with a median duration of 12 weeks and a median of 3 (range 2–7) sessions/week. Aerobic training was performed in 36 studies, resistance training in 12 studies, a combination of aerobic and resistance training in eight studies, and HIIT in 11 studies. Exercise duration was prescribed in minutes or as energy expenditure (kcal), and the exercise intensity defined as percentage of maximal aerobic capacity (VO_2_max) or heart rate (HRmax or HRReserve). The mean (range) exercise prescription was 45 (17–90) min per session or 1698 (668–2430) kcal/week at 60 (45–85)% VO_2_max, 70 (50–85)% HRmax, or 60 (45–80)% HRReserve for aerobic training. Resistance training was prescribed with a median (range) of 3 (2–4) sets of 10 (8–15) repetitions for 8 (4–12) exercises at 70 (60–85)% of 1 RM. HIIT was prescribed as a median (range) of 5 (3–11) bouts of 128 (30–480) s at 90 (85–95)% VO_2_max or 90 (80–100)% HRmax with a median (range) recovery duration of 120 (45–180) s at 50 (20–70)% VO_2_max or 70 (70–70)% HRmax. Four studies evaluated exercise intensity as rate of perceived exertion (RPE) measured with Borg scale with a median value of 14 (13–18). One study prescribed exercise training at individualized VO_2_, corresponding to maximal fat oxidation (34.5%, range 24.4–46.8% VO_2_max). Exercise sessions were fully supervised in 28 studies, partially supervised in 14 studies, and not supervised in five studies. Information about supervision was not reported in seven studies. Supervision was conducted by physical therapists, physicians, fitness instructors, exercise physiologists or specialists, or kinesiologists. Control groups were characterized by no exercise prescription, sham exercise (i.e., stretching), usual care, or dietary prescription, if the intervention group received a diet prescription plus exercise. One study compared exercise and rosiglitazone versus rosiglitazone alone considered as control group. Reported outcomes included systolic blood pressure (36 studies), diastolic blood pressure (35 studies), HOMA‐IR (30 studies), and intrahepatic fat (13 studies). Except for two RCTs that assessed outcome after the conclusion of the exercise intervention program 6‐^54^ and 8‐month follow‐ups,^30^ all RCTs reported measures recorded immediately after the intervention period. When studies reported more than one follow‐up measure, those assessed immediately after the conclusion of the intervention period were chosen.

### 3.2 Study findings

Findings of the included studies are listed in Table S4.

#### 3.2.1 Blood pressure

Included outcomes were systolic and diastolic blood pressure assessed as office measures. Only two RCTs^48^, ^63^ used 24‐h blood pressure monitoring (ABPM), and the mean of daytime measures was included in meta‐analyses. Nine studies included only subjects with prehypertension or hypertension, six studies included only subjects with normal blood pressure, and 16 studies included mixed populations, that is, subjects with or without hypertension.

#### 3.2.2 Systolic blood pressure

**FIGURE 2:**
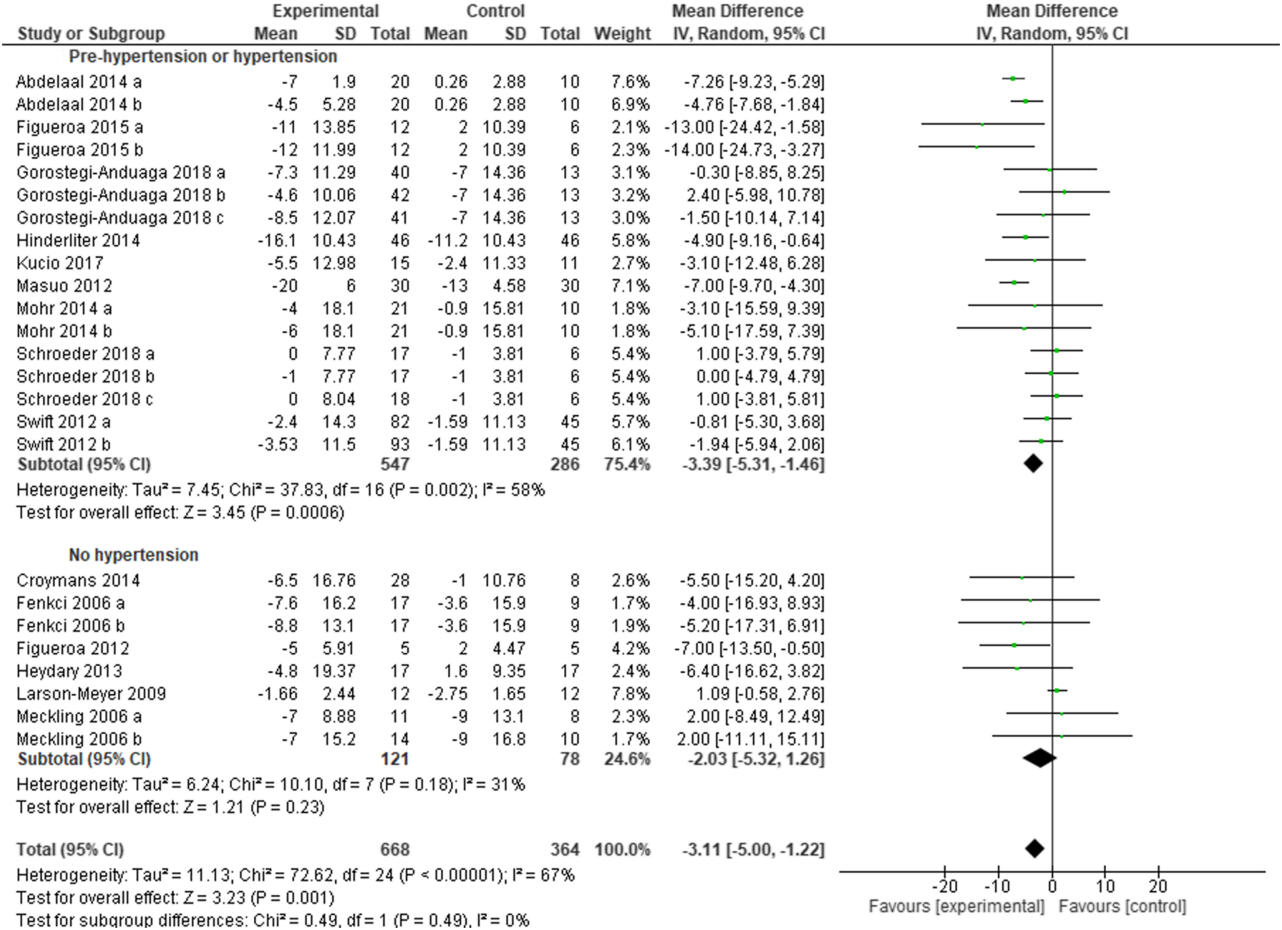
Forest plot of the effect of exercise training versus control on systolic blood pressure in adults with overweight or obesity, grouped by presence/absence of arterial hypertension. Presents mean difference between subjects participating in exercise training versus control in change of systolic blood pressure. Subgroup “Normotensive patients” includes randomized controlled trials (RCTs) that excluded patients with overweight or obesity and hypertension. Subgroup “Pre‐hypertension or hypertension” includes RCTs that included only patients with overweight or obesity and prehypertension or hypertension. Articles are presented in alphabetical order. Abdelaal 2014 (a): aerobic exercise; Abdelaal 2014 (b): resistance exercise. Fenkci (a): aerobic training; Fenkci (b): resistance training. Figueroa 2015 (a): high ankle blood pressure; Figueroa 2015 (b): low ankle blood pressure. Gorostegi‐Anduaga 2018 (a): high volume‐moderate intensity continuous training; Gorostegi‐Anduaga 2018 (b): high volume‐high intensity interval training; Gorostegi‐Anduaga 2018 (c): low volume‐high intensity interval training. Meckling (a): control diet + exercise versus control diet; Meckling (b): high protein diet + exercise versus high‐protein diet. Mohr 2014 (a): moderate intensity continuous training versus control. Mohr 2014 (b): high intensity interval training versus control. Schroeder 2018 (a): aerobic exercise; Schroeder 2018 (b): resistance exercise; Schroeder 2018 (c): combined exercise. Swift 2012 (a): lower energy expenditure (8 kcal/kg/week); Swift 2012 (b): higher energy expenditure (12 kcal/kg/week)

To assess the overall effect of exercise on systolic blood pressure, all studies were included in the meta‐analysis, whatever the blood pressure status (60 study arms, 1334 subjects in the experimental groups, and 942 subjects in the control groups). As shown in Figure S1, exercise training programs were effective in reducing systolic blood pressure (MD: −2.95 mmHg [95% CI: −4.22, −1.68], *p* < 0.00001, *I* ^2^ = 63%). When including only studies with hypertensive subjects (17 study arms), blood pressure was reduced on average by −3.39 mmHg (95% CI −5.31, −1.46), *p* = 0.0006, *I* ^2^ = 58% (Figure 2). When including only studies with normotensive subjects (eight study arms), the effect was not significant (MD: −2.03 mmHg [95% CI: −5.32, 1.26], *p* = 0.23, *I* ^2^ = 31%). Funnel plots all looked symmetric (Figure S5a).

Sensitivity analyses were conducted according to study quality. Out of the 36 studies included in the meta‐analyses, 24 studies were rated as fair or good quality and 12 as poor quality (Table S2). When eliminating the poor‐quality studies, the overall effect on systolic blood pressure remained unchanged (MD: −3.04 mmHg [95% CI: −4.37, −1.71], *p* < 0.00001, *I* ^2^ = 54%). Sensitivity analyses performed by eliminating the two studies that used ABPM did not reveal substantial differences in the overall MD. One‐study removing sensitivity analyses did not change the overall result.

#### 3.2.3 Diastolic blood pressure

**FIGURE 3:**
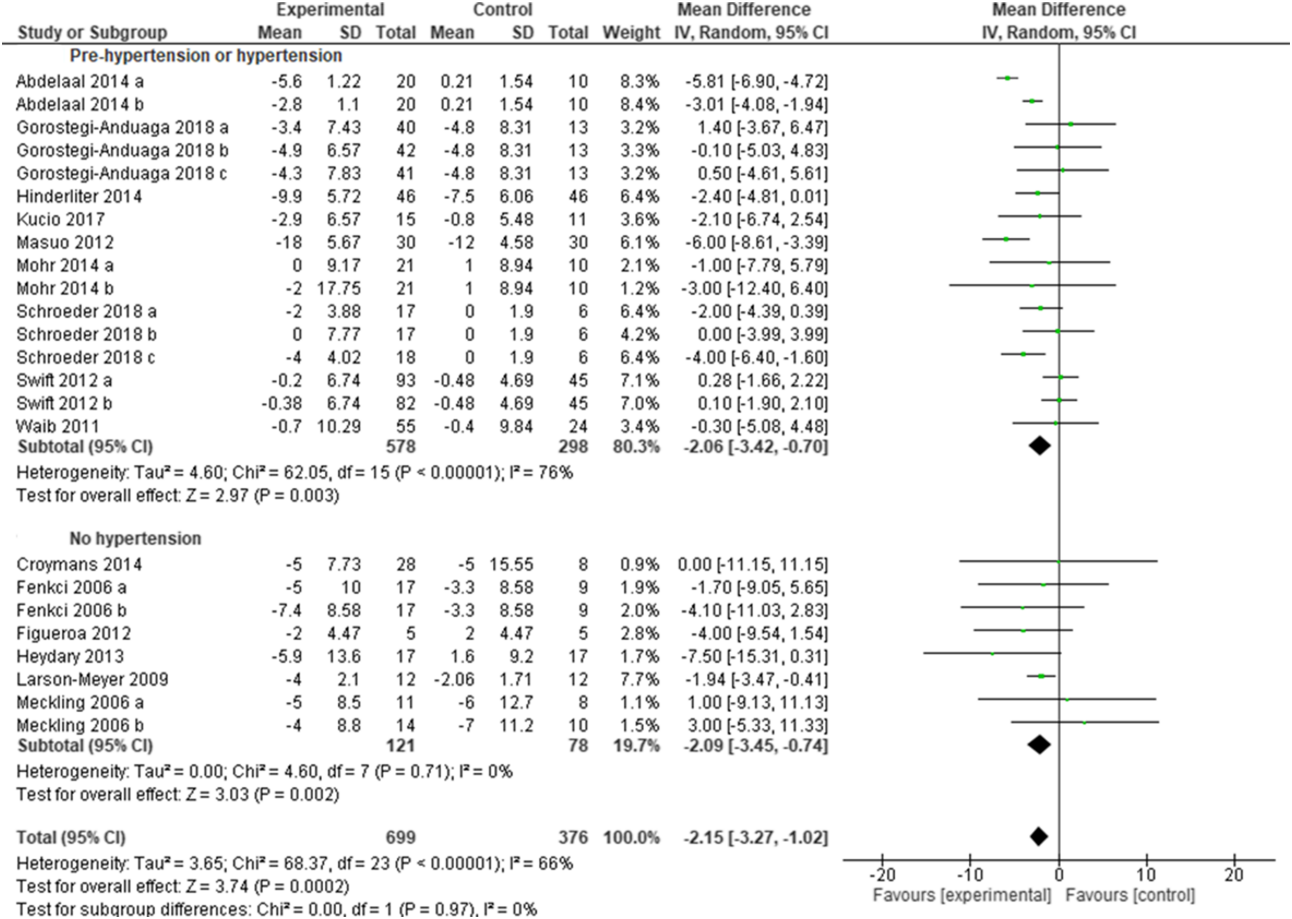
Forest plot of the effect of exercise training versus control on diastolic blood pressure in adults with overweight or obesity, grouped by presence/absence of arterial hypertension. Presents mean difference between subjects participating in exercise training versus control in change of diastolic blood pressure. Articles are presented in alphabetical order. Subgroup “Normotensive patients” includes randomized controlled trials (RCTs) that excluded patients with overweight or obesity and hypertension. Subgroup “Pre‐hypertension or hypertension” includes RCTs that included only patients with overweight or obesity and prehypertension or hypertension. Abdelaal 2014 (a): Aerobic exercise; Abdelaal 2014 (b): resistance exercise. Fenkci (a): aerobic training; Fenkci (b): resistance training; Gorostegi‐Anduaga 2018 (a): high volume‐moderate intensity continuous training; Gorostegi‐Anduaga 2018 (b): high volume‐high intensity interval training; Gorostegi‐Anduaga 2018 (c): low volume‐high intensity interval training. Meckling (a): control diet + exercise versus control diet; Meckling (b): high protein diet + exercise versus high protein diet. Mohr 2014 (a): moderate intensity continuous training versus control; Mohr 2014 (b): high intensity interval training versus control. Schroeder 2018 (a): aerobic exercise; Schroeder 2018 (b): resistance exercise; Schroeder 2018 (c): combined exercise. Swift 2012 (a): higher energy expenditure (12 kcal/kg/week); Swift 2012 (b): lower energy expenditure (8 kcal/kg/week)

A total of 58 study arms (1310 subjects in the experimental groups and 930 subjects in the control groups) were included to assess the overall effect of exercise training programs on diastolic blood pressure (whatever the initial blood pressure status). As shown in Figure S2, exercise training programs were effective in reducing diastolic blood pressure (MD: −1.93 mmHg [95% CI: −2.73, −1.13], *p* < 0.00001, *I* ^2^ = 54%). Blood pressure decreased significantly with exercise, both when including only studies with hypertensive subjects (16 study arms), (MD: −2.06 mmHg [95% CI: −3.42, −0.70], *p* = 0.003, *I* ^2^ = 76%), and only studies with normotensive subjects (eight study arms) MD: −2.09 mmHg (95% CI: −3.45, −0.74), *p* = 0.002, *I* ^2^ = 0%), as reported in Figure 3. Funnel plots demonstrated a symmetric distribution (Figure S5b).

Twenty‐three studies were rated as fair or good quality and 12 as poor quality (Table S2). When eliminating the poor‐quality studies, the overall effect of exercise was comparable (MD: −1.98 mmHg [95% CI: −2.87, −1.09], *p* < 0.0001, *I* ^2^ = 54%). Sensitivity analyses performed by eliminating the two studies that used ABPM did not reveal substantial differences in the overall MD. One study removing sensitivity analyses did not change the overall result.

#### 3.2.4 Insulin resistance

**FIGURE 4:**
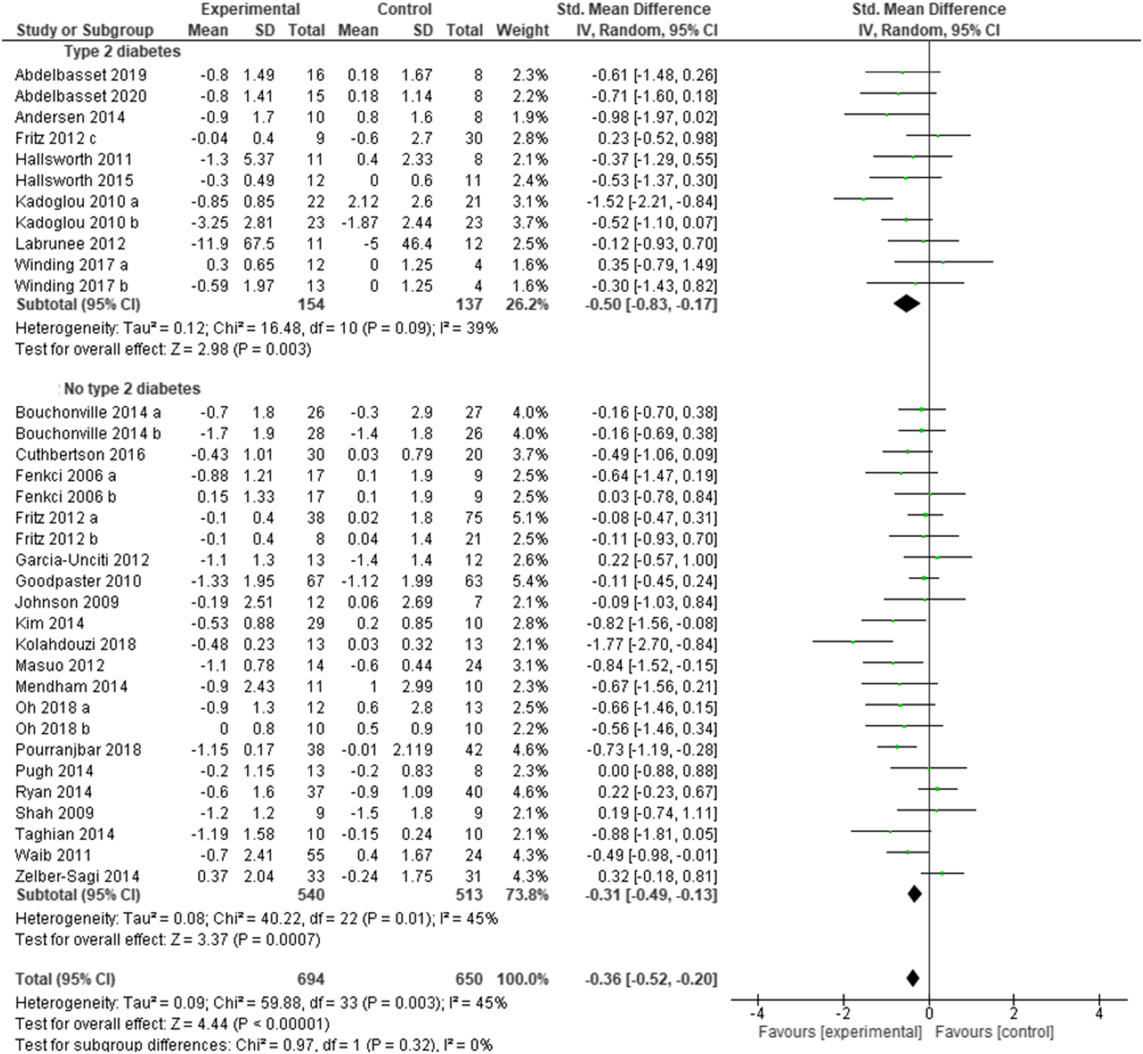
Forest plot of the effect of exercise training versus control on Homeostasis Model Assessment Insulin Resistance Index (HOMA‐IR) in adults with overweight or obesity, grouped by presence/absence type 2 diabetes. Presents mean difference between subjects participating in exercise training versus control in change of HOMA‐IR. Articles are presented in alphabetical order. Subgroup “No type 2 diabetes” includes randomized controlled trials (RCTs) that excluded patients with overweight or obesity and type 2 diabetes. Subgroup “Type 2 diabetes” includes RCTs that included only patients with overweight or obesity and type 2 diabetes. Articles are presented in alphabetical order. Bouchonville 2014 (a): exercise versus control. Bouchonville 2014 (b):diet + exercise versus diet; Fenkci (a): aerobic training; Fenkci (b): resistance training. Fritz 2012 (a): normal glucose tolerance; Fritz 2012 (b): impaired glucose tolerance; Fritz 2012 (c): type 2 diabetes. Kadoglou 2010 (a): exercise versus control; Kadoglou 2010 (b): rosiglitazone + exercise versus rosiglitazone. Oh 2018 (a): diet + exercise versus diet; Oh 2018 (b): exercise versus control; Winding 2014 (a): endurance training; Winding 2014 (b): high intensity interval training

Out of the 30 RCTs that reported HOMA‐IR, 18 studies included subjects without type 2 diabetes, eight studies included subjects with type 2 diabetes, one study assessed three arms of intervention based on the glycaemic status, and three studies provided no information or included mixed populations. A total of 37 study arms (740 subjects in the experimental groups and 697 subjects in the control groups) were included to assess the overall effect of exercise training programs on insulin resistance (whatever the initial glycemic status). As shown in Figure S3, exercise reduced HOMA‐IR (SMD: −0.34 [95% CI: −0.49, −0.18], *p* < 0.0001, *I* ^2^ = 48%). HOMA‐IR decreased significantly when we included only interventions performed in subjects with type 2 diabetes (SMD: −0.50 [95% CI: −0.83, −0.17], *p* = 0.003, *I* ^2^ = 39%) or only interventions performed in subjects without type 2 diabetes (SMD: −0.31 [95% CI: −0.49, −0.13], *p* = 0.0007, *I* ^2^ = 45%) (Figure 4). Funnel plots demonstrated a symmetric distribution (Figure S5c).

Fifteen studies were rated as fair or good quality and 15 as poor quality. Removing the poor‐quality studies did not change the overall effect of exercise (SMD: −0.38 [95% CI: −0.56, −0.20], *p* < 0.0001, *I* ^2^ = 34%). One study removing sensitivity analyses did not change the overall result.

#### 3.2.5 Intrahepatic fat

**FIGURE 5:**
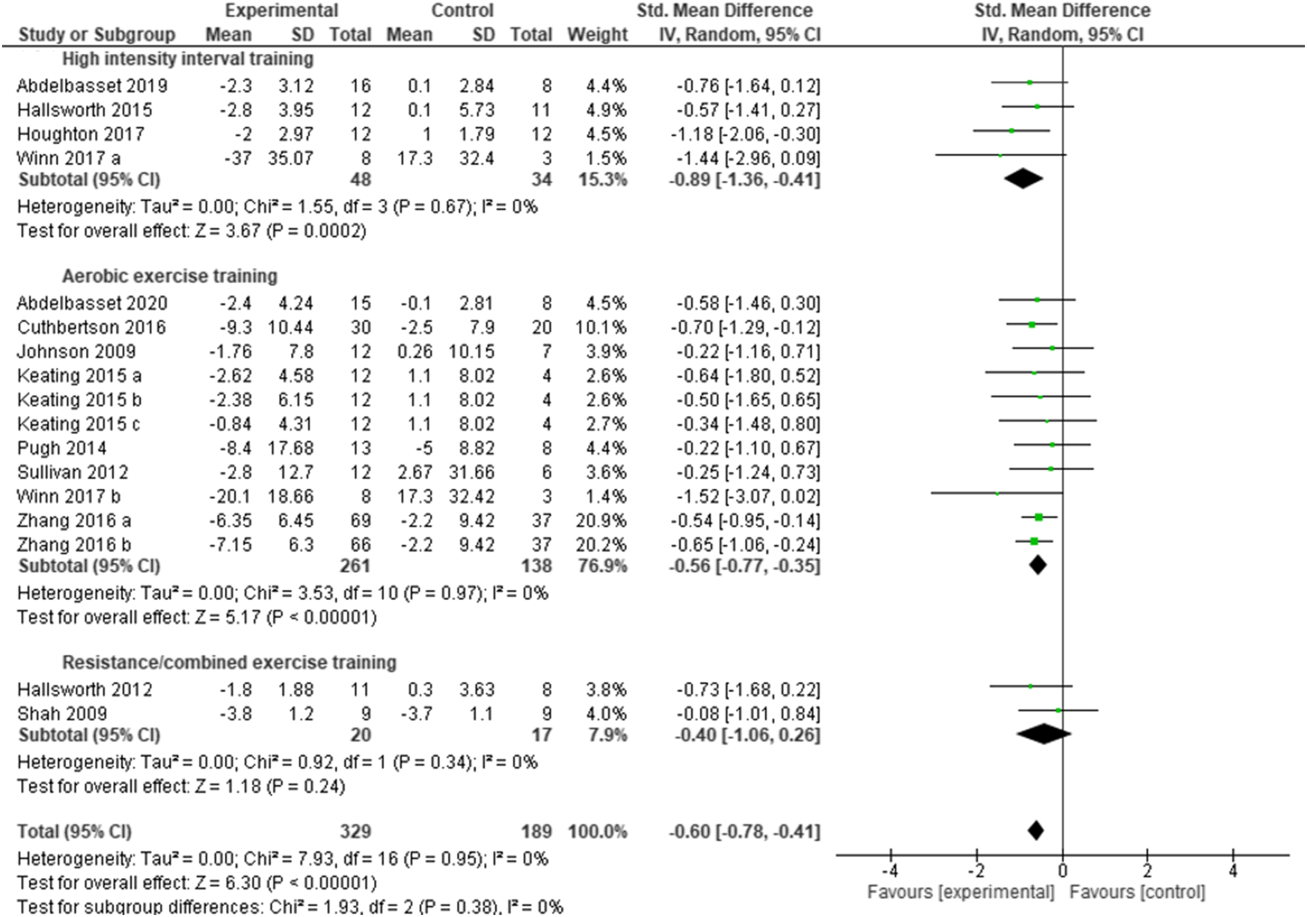
Pooled analysis for the effect of exercise training versus control on intrahepatic fat in adults with overweight or obesity, grouped by exercise modality. Presents mean difference between subjects participating in exercise training versus control in change of intrahepatic fat. Articles are presented in alphabetical order. Keating 2015 (a): high intensity, low volume aerobic exercise; Keating 2015 (b): low intensity, high volume aerobic exercise. Keating 2015 (c): low intensity, low volume aerobic exercise. Winn 2017 (a): high intensity interval training; Winn 2017 (b): moderate intensity continuous training; Zhang 2016 (a): moderate intensity training; Zhang (b): vigorous intensity training

Out of the 13 studies assessing the effect of exercise on MR measures of intrahepatic fat, nine studies included subjects with NAFLD, one study included subjects with NASH, and three studies included mixed populations (with and without NAFLD). A total of 17 study arms (329 subjects in the experimental groups and 189 subjects in the control groups) were included to assess the overall effect of exercise training programs on intrahepatic fat (whatever the initial status of participants and the type of exercise). Exercise reduced intrahepatic fat (SMD: −0.59 [95% CI: −0.78, −0.41], *p* < 0.00001, *I* ^2^ = 0%) (Figure S4). The funnel plot was symmetric (Figure S5d). Meta‐analyses stratified according to the type of exercise training (Figure 5) showed that HIIT, aerobic, and resistance alone or combined with aerobic training all decreased intrahepatic fat (SMD: −0.89 [95% CI: −1.36, −0.41], *p* = 0.0002, *I* ^2^ = 0%; SMD: −0.56 [95% CI: −0.77, −0.35], *p* < 0.00001, *I* ^2^ = 0%; and SMD: −0.40 [95% CI −1.06, 0.26], *p* = 0.24, *I* ^2^ = 0%, respectively).

Nine studies were rated as fair or good quality and four as poor quality. Removing the poor‐quality studies, the overall effect of exercise did not change (SMD: −0.59 [95% CI: −0.80, −0.38], *p* < 0.00001, *I* ^2^ = 0%). One study removing sensitivity analyses did not change the overall result.

### 3.3 Study quality

Study quality was rated as good, fair, and poor in 11 (20%), 20 (37%), and 23 (43%) studies, respectively (Table S2). Most RCTs did not perform intention to treat analysis (Criterion 14), blinding of participants was generally not applicable (Criterion 4), and blinding of allocation and of the people assessing outcomes (Criteria 3 and 5) was not accomplished in more than half of the RCTs.

## 4 DISCUSSION

This systematic review and meta‐analysis provides a comprehensive analysis on the effect of exercise training interventions on key aspects of cardiometabolic health in adults with overweight or obesity. Our results demonstrate that exercise training programs are effective in improving cardiometabolic health in adults with overweight or obesity, including patients living with obesity‐related comorbidities.

### 4.1 Blood pressure

An overall reduction in both systolic and diastolic blood pressure was observed. The reduction of systolic blood pressure, whatever the blood pressure status of participants, was about 2 mmHg. Similar reductions in blood pressure have been previously reported after exercise training, although the effect of exercise was not specifically assessed in subjects with overweight or obesity.^79^ Interestingly, we found that the decrease in blood pressure appeared to be larger in subjects with hypertension compared with normotensive patients, reaching on average 3 mmHg versus a nonsignificant effect. This is in line with results of Cornelissen and Smart that reported a larger effect of resistance exercise training on systolic blood pressure in subjects with hypertension compared with those with prehypertension or normal blood pressure.^80^ It should be noted, however, that even a small decrease in systolic blood pressure of 2 mmHg is associated with a 10% decrease in stroke mortality and 7% decrease in ischemic heart disease and vascular mortality in middle‐aged adults.^81^ Therefore, the observed reduction in blood pressure resulting from exercise training in subjects with overweight or obesity may bring substantial health benefits in the long term. Despite a relatively large number of studies included in our meta‐analysis (*N* = 54), the heterogeneity of participants, especially regarding their initial blood pressure status, was important and prevented us from comparing different types of exercise training. In studies enrolling only patients with hypertension, a majority of study arms was based on aerobic training; this enabled us to conclude that aerobic exercise is effective in reducing systolic and diastolic blood pressure in subjects with overweight or obesity and hypertension. A recent systematic review focusing on individuals with hypertension confirmed the efficacy of aerobic exercise and highlighted the potential role of HIIT in reducing blood pressure values with a greater magnitude.^82^ Also, resistance training seems to have similar effect in reducing blood pressure, with higher effect in patients with hypertension.^83^, ^84^, ^85^ These findings cannot yet be extended to hypertensive subjects with overweight or obesity because of the currently limited available data.

A further source of heterogeneity came from the interventions provided in addition to exercise training. One study, by Larson‐Meyer et al.,^38^ aimed to assess the effect of energy intake restriction versus energy intake restriction plus aerobic exercise, whereas the research of Kadoglu et al.^34^ was the only one combining exercise and drug treatment and one of the two with a long duration of the intervention (48 weeks). Nevertheless, the overall effect did not significantly change by eliminating these studies from the meta‐analysis. Although previous data have shown that the effect of exercise is higher when it is associated with weight loss,^86^ there are several exercise‐induced mechanisms that can explain blood pressure lowering, independently from weight loss.^87^ Indeed, changes in body composition, particularly when characterized by increased lean mass and decreased adipose tissue, especially ectopic fat, are able to improve the endocrine, metabolic, and inflammatory profile, with beneficial effects on blood pressure control.^35^, ^88^ In this connection, mice experiments demonstrated that increasing muscle mass improves obesity‐associated hypertension and that a plausible mechanism involves the relationship between glucose metabolism and the vascular and renal function.^89^ Accordingly, a previous meta‐analysis focused on the effect of dynamic resistance training in prehypertensive patients showed a significant reduction in systolic and diastolic blood pressure values, with a notable dose–response relationship.^90^ A link between myokines (i.e., myostatin) and arterial stiffness has also been recently described in healthy adolescents.^91^ Moreover, the improvement of endothelial function, arterial structure, and stiffness due to vasodilation and the reduction of vascular oxidative stress play also an important role in reducing blood pressure in response to exercise training.^92^, ^93^

### 4.2 Insulin resistance

Exercise training programs were effective in reducing insulin resistance in patients with overweight or obesity, with or without type 2 diabetes. Current results confirm findings of Thaane et al.^10^ who found a significant effect on insulin sensitivity, examining the effect of short term exercise training (<12 weeks) in subjects with overweight or obesity and type 2 diabetes. In addition, authors stated that the effect was poorly influenced by weight loss and was more pronounced for vigorous exercise. The efficacy of exercise was demonstrated also for dynamic measures of insulin sensitivity (i.e., clamp, oral glucose tolerance test, and insulin tolerance test), in patients with type 2 diabetes.^11^ It is known that physical exercise improves metabolic flexibility also by increasing insulin‐dependent glucose uptake, particularly in individuals with type 2 diabetes.^94^ Furthermore, physical exercise can enhance glucose metabolism not only in muscle but also in adipose and hepatic tissue,^95^ determining an overall improvement of insulin sensitivity.^96^ Overall, it is still unknown what the best exercise prescription would be (i.e., modality, volume, and intensity of exercise) to improve insulin sensitivity in patients with obesity and type 2 diabetes. Our systematic review found nine RCTs including only patients with type 2 diabetes, and only one performed resistance training, three performed HIIT, and five performed aerobic exercise. However, our subanalysis suggests that aerobic exercise, also including high intensity, reduces insulin resistance in patients with obesity and type 2 diabetes. Previous studies indicated that high‐intensity exercise had a greater beneficial effect than moderate intensity exercise on insulin resistance.^97^ Actually, high‐intensity exercise seems to have a more pronounced effect in recruiting glycolytic fibers,^98^ and in decreasing visceral fat,^99^ counteracting insulin resistance. Also for this outcome, heterogeneity was partly reduced by removing the study of Kadoglu et al.^34^ without changes in the overall effect. In this systematic review, it was not possible to identify the role played by exercise in improving insulin sensitivity, regardless of weight loss. Nevertheless, possible explanations for a higher insulin sensitivity can rely on the reduction in visceral adipose tissue and the virtuous hormonal cascade leading to a multiorgan improvement of glucose metabolism.^87^ On the other hand, also a quantitative and qualitative improvement in muscle mass induced by exercise training may explain the enhancement in insulin sensitivity observed in subjects with obesity with or without type 2 diabetes that followed exercise programs.^11^, ^100^, ^101^ From a clinical perspective, it is well‐known that enhancement of insulin sensitivity, rather than plasma glucose level, has a major role in improving diabetes outcomes, while insulin resistance is a better predictor of future cardiovascular events in nondiabetic individuals.^99^ Furthermore, there is compelling evidence that insulin resistance by itself is a cardiovascular risk factor in a variety of population groups, including the general population and patients with diabetes.^99^

### 4.3 Intrahepatic fat

Our results show the effective role of exercise training in reducing MR measures of intrahepatic fat in groups of adults with overweight or obesity. These findings are in line with previous reviews that pointed in the same direction. Keating et al. in 2012 showed that exercise training alone is effective in reducing intrahepatic fat in patients with NAFLD.^35^ Similarly, in 2017, Katsagoni et al. showed that exercise, alone or combined with dietary intervention, improves serum hepatic enzymes and intrahepatic fat (moderate to large effect size) in patients with NAFLD even in absence of weight loss.^13^ This well‐conducted systematic review included all types of intrahepatic fat measures (MR, ultrasound, and liver biopsy). Our work is focused on MR measures of intrahepatic fat, with 10 included studies using MR spectroscopy and the others with MR imaging. MR is the noninvasive method that better correlates with liver biopsy, recognized as the gold standard for the identification of liver steatosis. These MR techniques showed great accuracy and high intraindividual reproducibility.^102^ Hence, restricting meta‐analysis to studies that used these methods for intrahepatic fat quantification strengthen the reliability of our findings. In 2018, Smart et al. described the effect of exercise alone on surrogate markers of liver function, in subjects with overweight/obesity or fatty liver disease. The authors described a significant effect of exercise in reducing intrahepatic fat, particularly when selecting RCTs based on energy expenditure (at least 10,000 kcal for the total exercise program) and exercise without a diet intervention (only three studies included). Our pooled analyses showed that HIIT has the larger effect size in reducing intrahepatic fat, even though other exercise modalities also exhibited a statistically significant effect. Similar results were previously suggested by Katsagoni et al.,^13^ who showed that the effect on intrahepatic fat was larger for higher intensity and volume of training. These results can be achieved independently from weight loss.^13^ Several mechanisms can be responsible for the effect of exercise on NAFLD.^103^ In fact, it is well‐known that physical exercise is effective in decreasing hepatic lipogenesis^104^ and gluconeogenesis and in improving skeletal muscle glycogen synthesis, resulting in lower plasma and liver triglycerides^105^ and higher insulin sensitivity, also in patients with obesity.^106^ NAFLD shares common pathophysiological pathways with sarcopenia, such as insulin resistance and inflammation, and people that perform regular exercise have a lower probability to develop NAFLD. Indeed, a preserved muscle mass seems to be a key component for the exercise‐induced amelioration of fatty liver.^107^ Further experimental studies in rats report the increase of mitochondrial oxidation of lipids and the prevention or attenuation of the endoplasmic reticulum stress, a condition tightly linked to reactive oxygen species generation, chronic low‐grade inflammation, and apoptosis in the setting of metabolic diseases.^13^ The efficacy of exercise in reducing intrahepatic fat levels has important clinical implications. Indeed, liver fat content is considered a sensitive “barometer” for metabolic health.^108^ Intrahepatic fat accumulation leads to NAFLD, a condition recognized as the hepatic manifestation of the metabolic syndrome and metabolically related with adipose tissue insulin resistance and diabetes pathophysiology.^109^

## 5 LIMITATIONS AND PERSPECTIVES

Physical exercise and pharmacological treatments are both key components for the management of obesity and its related comorbidities. Their interactive effects might be explained by shared metabolic and vascular mechanisms, with a potential synergistic or compensatory effect on cardiometabolic health.^110^, ^111^, ^112^ However, future studies should explore the actual relationship between antidiabetic and/or anti‐obesity agents and exercise, in order to identify the best combination of pharmacological and nonpharmacological individualized treatment for each patient. Moreover, previous literature described that the response to lifestyle intervention for cardiometabolic health may be influenced by several factors such as sex and age, highlighting the importance of tailored actions.^113^, ^114^ Our current systematic review and meta‐analysis did not address these interesting aspects; thus, upcoming literature may focus on the analysis of the differential response to exercise therapy linked to age, sex, and the age–sex interaction.

Additional potential limitation is that data were often retrieved from secondary outcomes of the selected studies, introducing a potential inaccuracy tied to the power calculation of each study. Finally, most RCTs evaluated the effect of aerobic exercise; as a result, pooled analyses based on exercise modality were not performed for blood pressure and insulin resistance.

## 6 CONCLUSIONS

In conclusion, this systematic review and meta‐analysis showed a beneficial effect of physical exercise training in improving cardiometabolic health of adults with overweight or obesity. In these subjects, blood pressure, insulin resistance, and intrahepatic fat all decreased significantly after an exercise training program. Given the prevalence of these cardiometabolic risk factors in people with overweight or obesity, such improvements might bring substantial health benefits in this population. Our results indicate that blood pressure and insulin resistance may decrease to a larger extent in subjects with hypertension and with type 2 diabetes, respectively, reinforcing the importance of physical activity in people with overweight or obesity and comorbidities. Aerobic training has many well‐known benefits on cardiometabolic health and was indeed the most frequent type of exercise assessed in studies included in our review. We were able to assess the effect of different types of exercise training (aerobic, combined aerobic and resistance, and HIIT) on intrahepatic fat, and we found that all types of exercise were effective in reducing intrahepatic fat. Therefore, given the multiple benefits of exercise on major aspects of cardiometabolic health, individualized exercise prescription should be more broadly included in the management of people with overweight or obesity.

## AUTHOR CONTRIBUTIONS

FB and AE performed the literature search, study selection, data extraction, and quality assessment. FB performed the meta‐analysis. All authors participated in the interpretation of data. FB and AE drafted the manuscript, and authors critically revised the manuscript.

## CONFLICT OF INTEREST

The authors declare that they have no conflict of interest.

## Supporting information

**Table S1.** Keywords included in database search strategy
Figure S1. Effect of exercise training vs. control on systolic blood pressure in adults with overweight or obesity.
Figure S2. Forest plot Effect of exercise training vs. control on diastolic blood pressure in adults with overweight or obesity.
Figure S3. Forest plot of the effect of exercise training programmes vs. control on HOMA‐IR in adults with overweight or obesity.
Figure S4. Forest plot of the effect of exercise training programmes vs. control on intrahepatic fat in adults with overweight or obesity.
Figure S5. Funnel plot.
Table S2. Summary of quality assessment of original studies.
Table S3. Characteristics of original studies
Table S4. Findings of original studies.
