# Supplementary material for: Effect of exercise on cardiometabolic health of adults with overweight or obesity: Focus on blood pressure, insulin resistance, and intrahepatic fat—A systematic review and meta‐analysis
Source: Obes Rev. 2021 May 6;22(Suppl 4):e13269. doi: 10.1111/obr.13269 (PMC8365642; doi:10.1111/obr.13269)
Supplement: Supplementary file 1 — Table S1. Keywords included in database search strategy Figure S1. Effect of exercise training vs. control on systolic blood pressure in adults with overweight or obesity. Figure S2. Forest plot Effect of exercise training vs. control on diastolic blood pressure in adults with overweight or obesity. Figure S3. Forest plot of the effect of exercise training programmes vs. control on HOMA‐IR in adults with overweight or obesity. Figure S4. Forest plot of the effect of exercise training programmes vs. control on intrahepatic fat in adults with overweight or obesity. Figure S5. Funnel plot. Table S2. Summary of quality assessment of original studies. Table S3. Characteristics of original studies Table S4. Findings of original studies. [file OBR-22-e13269-s001.pdf]

SUPPORTING INFORMATION

Effect of exercise on cardio-metabolic health of adults with overweight or obesity: focus on blood pressure, insulin resistance and intrahepatic fat. A systematic review and meta-analysis.

Francesca Battista[1], Andrea Ermolao* [1], Marleen A. van Baak[2], Kristine Beaulieu[3], John E. Blundell[3], Luca Busetto[4,5], Eliana V. Carraça[6], Jorge Encantado[7], Dror Dicker[4,8], Nathalie Farpour-Lambert[4,9], Adriyan Pramono[2], Alice Bellicha[10,11], Jean-Michel Oppert[12]

Corresponding Author

Andrea Ermolao

Department of Medicine

University of Padova

Via Giustiniani, 2

Padova, 35128, Italy

Tel: +39 049 8215676

E-mail: andrea.ermolao@unipd.it

Table S1. Keywords included in database search strategy

**Obesity**	**Physical activity**	**Age**	**Comorbidities**
Overweight<br>Obesity<br>Obese	Physical activit*<br>Exercise<br>Sport<br>Endurance activity<br>Endurance activities<br>Aerobic activity<br>Aerobic activities<br>Cardiovascular activit*<br>Resistance training<br>Strength training<br>Muscle-strengthening<br>Weight-Lifting program<br>High-intensity interval training<br>HIIT<br>Physical conditioning<br>Walking<br>Sedentary time<br>Sedentary lifestyle<br>Sitting time	Adults<br>(NOT child, children, adolescents, pediatric)	Type 2 diabetes<br>Type 2 diabetes mellitus<br>NIDDM<br>noninsulin-dependent diabetes<br>insulin resistance<br>hyperglycemia<br>glucose intolerance<br>hypertension<br>high blood pressure<br>fatty liver disease<br>non-alcoholic fatty liver disease<br>non-alcoholic steatohepatitis<br>NAFLD<br>NASH

Figure S1. Effect of exercise training vs. control on systolic blood pressure in adults with overweight or obesity.

Study or Subgroup	Experimental Mean	Experimental SD	Experimental Total	Control Mean	Control SD	Control Total	Weight	Mean Difference IV, Random, 95% CI
Abdelaal 2014 a	-7	1.9	20	0.26	2.88	10	3.3%	-7.26 [-9.23, -5.29]
Abdelaal 2014 b	-4.5	5.28	20	0.26	2.88	10	3.0%	-4.76 [-7.68, -1.84]
Balducci 2012	-7	17.7	36	-3	17.05	34	1.5%	-4.00 [-12.14, 4.14]
Bouchonville 2014 a	-15.9	18.9	28	-13.1	15.1	26	1.3%	-2.80 [-11.89, 6.29]
Bouchonville 2014 b	-1.2	15.5	26	-5.9	23	27	1.0%	4.70 [-5.82, 15.22]
Cao 2019	-4	16	13	0	14	15	0.9%	-4.00 [-15.22, 7.22]
Croymans 2014	-6.5	16.76	28	-1	10.76	8	1.2%	-5.50 [-15.20, 4.20]
Fenkci 2006 a	-7.6	16.2	17	-3.6	15.9	9	0.8%	-4.00 [-16.93, 8.93]
Fenkci 2006 b	-8.8	13.1	17	-3.6	15.9	9	0.8%	-5.20 [-17.31, 6.91]
Figueroa 2012	-5	5.91	5	2	4.47	5	1.8%	-7.00 [-13.50, -0.50]
Figueroa 2015 a	-11	13.85	12	2	10.39	6	0.9%	-13.00 [-24.42, -1.58]
Figueroa 2015 b	-12	11.99	12	2	10.39	6	1.0%	-14.00 [-24.73, -3.27]
Fritz 2012 a	2	11.9	38	-2.1	11.9	75	2.4%	4.10 [-0.54, 8.74]
Fritz 2012 b	-13.1	10.7	8	1.6	11.1	21	1.3%	-14.70 [-23.50, -5.90]
Fritz 2012 c	3.9	13.9	9	-3.8	15.2	30	1.0%	7.70 [-2.89, 18.29]
Goodpaster 2010	-3.39	14.39	67	-1.83	14.9	63	2.3%	-1.56 [-6.60, 3.48]
Gorostegi-Anduaga 2018 a	-7.3	11.29	40	-7	14.36	13	1.4%	-0.30 [-8.85, 8.25]
Gorostegi-Anduaga 2018 b	-4.6	10.06	42	-7	14.36	13	1.4%	2.40 [-5.98, 10.78]
Gorostegi-Anduaga 2018 c	-8.5	12.07	41	-7	14.36	13	1.3%	-1.50 [-10.14, 7.14]
Heydary 2013	-4.8	19.37	17	1.6	9.35	17	1.1%	-6.40 [-16.62, 3.82]
Hinderliter 2014	-16.1	10.43	46	-11.2	10.43	46	2.6%	-4.90 [-9.16, -0.64]
Ho 2012 a	0.6	15.76	15	-4	9.1	5	0.9%	4.60 [-6.68, 15.88]
Ho 2012 b	-1.69	18.92	16	-4	9.1	5	0.8%	2.31 [-9.92, 14.54]
Ho 2012 c	-4.95	14.29	17	-4	9.1	5	1.0%	-0.95 [-11.43, 9.53]
Kadoglou 2010 a	-6.29	4.25	22	0.79	2.54	21	3.3%	-7.08 [-9.16, -5.00]
Kadoglou 2010 b	-13.08	6.28	23	-6.38	3.22	23	3.0%	-6.70 [-9.58, -3.82]
Keating 2015 a	-4.83	8.62	12	1.33	3.98	4	1.9%	-6.16 [-12.40, 0.08]
Keating 2015 b	-5.6	7.06	12	1.33	3.98	4	2.1%	-6.93 [-12.51, -1.35]
Keating 2015 c	5.76	6.06	12	1.33	3.98	4	2.2%	4.43 [-0.76, 9.62]
Kim 2012	-8.63	4.86	15	2.15	3.47	15	3.0%	-10.78 [-13.80, -7.76]
Kozey-Keadle 2014 a	-7	9.95	16	-5.9	8.58	8	1.5%	-1.10 [-8.79, 6.59]
Kozey-Keadle 2014 b	-5.9	8.1	16	-4.7	11.16	14	1.7%	-1.20 [-8.27, 5.87]
Kucio 2017	-5.5	12.98	15	-2.4	11.33	11	1.2%	-3.10 [-12.48, 6.28]
Larson-Meyer 2009	-1.66	2.44	12	-2.75	1.65	12	3.4%	1.09 [-0.58, 2.76]
Masuo 2012	-20	6	30	-13	4.58	30	3.1%	-7.00 [-9.70, -4.30]
Meckling 2006 a	-7	8.88	11	-9	13.1	8	1.0%	2.00 [-8.49, 12.49]
Meckling 2006 b	-7	15.2	14	-9	16.8	10	0.7%	2.00 [-11.11, 15.11]
Mendham 2014	-0.3	8.2	11	-3.7	13.76	10	1.1%	3.40 [-6.41, 13.21]
Mohr 2014 a	-4	18.1	21	-0.9	15.81	10	0.8%	-3.10 [-15.59, 9.39]
Mohr 2014 b	-6	18.1	21	-0.9	15.81	10	0.8%	-5.10 [-17.59, 7.39]
Oh 2018 a	-5.8	8.8	8	0	8.1	8	1.4%	-5.80 [-14.09, 2.49]
Oh 2018 b	-3.3	8.3	10	-1.8	5.8	9	1.9%	-1.50 [-7.89, 4.89]
Plotnikoff 2010	-2.7	11.22	27	-0.3	11.76	21	1.8%	-2.40 [-8.97, 4.17]
Pugh 2014	-0.5	8.1	13	-2	7.53	8	1.8%	1.50 [-5.33, 8.33]
Ryan 2014	-5	12.6	37	-3	10.94	40	2.2%	-2.00 [-7.29, 3.29]
Schroeder 2018 a	0	7.77	17	-1	3.81	6	2.4%	1.00 [-3.79, 5.79]
Schroeder 2018 b	-1	7.77	17	-1	3.81	6	2.4%	0.00 [-4.79, 4.79]
Schroeder 2018 c	0	8.04	18	-1	3.81	6	2.4%	1.00 [-3.81, 5.81]
Shah 2009	-24.4	12.6	9	-19.9	18.9	9	0.6%	-4.50 [-19.34, 10.34]
Stensvold 2010 a	-5.5	8.78	11	0.7	3.95	4	1.9%	-6.20 [-12.67, 0.27]
Stensvold 2010 b	-4.2	11.46	10	0.7	3.95	4	1.5%	-4.90 [-12.99, 3.19]
Stensvold 2010 c	-2.8	9.08	11	0.7	3.95	4	1.8%	-3.50 [-10.12, 3.12]
Straznicky 2012	-10.3	10.5	19	-10.5	9.9	20	1.9%	0.20 [-6.21, 6.61]
Swift 2012 a	-2.4	14.3	82	-1.59	11.13	45	2.5%	-0.81 [-5.30, 3.68]
Swift 2012 b	-3.53	11.5	93	-1.59	11.13	45	2.6%	-1.94 [-5.94, 2.06]
Tjonna 2008 a	-10	17.61	8	-5	12.45	5	0.5%	-5.00 [-21.37, 11.37]
Tjonna 2008 b	-9	17.32	11	-5	12.45	5	0.6%	-4.00 [-18.96, 10.96]
Waib 2011	-0.3	11.48	55	0	10.72	24	2.2%	-0.30 [-5.55, 4.95]
Winding 2017 a	-1	19.97	12	4	8.18	4	0.7%	-5.00 [-18.85, 8.85]
Winding 2017 b	-1	15.09	13	4	8.18	4	0.9%	-5.00 [-16.47, 6.47]
**Total (95% CI)**			**1334**			**942**	**100.0%**	**-2.95 [-4.22, -1.68]**

Heterogeneity: Tau² = 11.78; Chi² = 159.69, df = 59 (P < 0.00001); I² = 63%
Test for overall effect: Z = 4.54 (P < 0.00001)

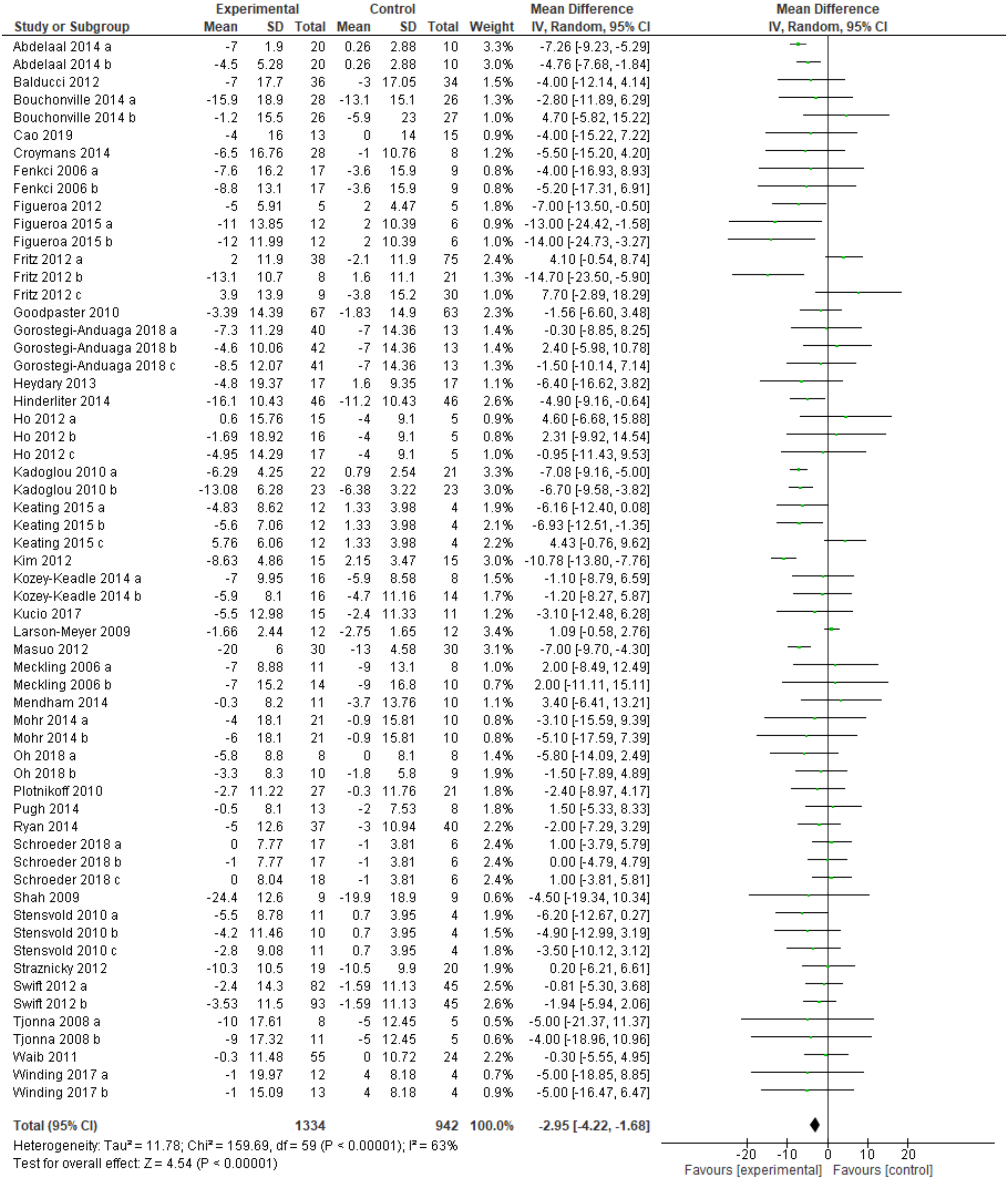

Articles are presented in alphabetical order. Abdelaal 2014 (a): Aerobic exercise; Abdelaal 2014 (b): resistance exercise. Bouchonville 2014 (a): diet+exercise vs. diet; Bouchonville 2014 (b): exercise vs. control. Fenkci (a): aerobic training; Fenkci (b): resistance training; Figueroa 2015 (a): high ankle blood pressure; Figueroa 2015 (b): low ankle blood pressure. Fritz 2012 (a): normal glucose tolerance; Fritz 2012 (b): impaired glucose tolerance; Fritz 2012 (c): type 2 diabetes. Gorostegi-Anduaga 2018 (a): moderate intensity continuous training; Gorostegi-Anduaga 2018 (b): high volume-high intensity interval training; Gorostegi-Anduaga 2018 (c): low volume-high intensity interval training. Ho 2012 (a): aerobic exercise; Ho 2012 (b): resistance exercise; Ho 2012 (c): combined exercise. Kadoglou 2010 (a): exercise vs. control; Kadoglou 2010 (b): rosiglitazone+exercise vs. rosiglitazone. Keating 2015 (a): high intensity, low volume aerobic exercise; Keating 2015 (b): low intensity, high volume aerobic exercise; Keating (c): low intensity, low volume aerobic exercise. Kozey-Keadle 2014 (a): exercise vs. control; Kozey-Keadle 2014 (b): exercise+reducing sitting time vs. reducing sitting time. Meckling (a): control diet+exercise vs. control diet; Meckling (b): high protein diet+exercise vs. high protein diet. Mohr 2014 (a): moderate intensity continuous training vs. control; Mohr 2014 (b): high intensity interval training vs. control. Oh 2018 (a): exercise vs. control; Oh 2018 (b): diet+exercise vs. diet. Schroeder 2018 (a): aerobic exercise; Schroeder 2018 (b): resistance exercise; Schroeder 2018 (c): combined exercise. Stensvold 2010 (a): aerobic interval training; Stensvold 2010 (b): combined training; Stensvold 2010 (c): strength training. Swift 2012 (a): lower energy expenditure (8 kcal/Kg/week); Swift 2012 (b): lower energy

expenditure (12 kcal/Kg/week). Tjonna 2008 (a): moderate intensity continuous training; Tjonna 2008 (b): aerobic interval training. Winding 2014 (a): endurance training; Winding 2014 (b): high intensity interval training.

Figure S2. Forest plot Effect of exercise training vs. control on diastolic blood pressure in adults with overweight or obesity.

	Experimental			Control				Mean Difference
Study or Subgroup	Mean	SD	Total	Mean	SD	Total	Weight	IV, Random, 95% CI
Abdelaal 2014 a	-5.6	1.22	20	0.21	1.54	10	4.2%	-5.81 [-6.90, -4.72]
Abdelaal 2014 b	-2.8	1.1	20	0.21	1.54	10	4.2%	-3.01 [-4.08, -1.94]
Balducci 2012	-3	9.85	36	-2	9.64	34	1.8%	-1.00 [-5.57, 3.57]
Bouchonville 2014 a	-2.1	6.1	26	-1.1	11.2	27	1.7%	-1.00 [-5.83, 3.83]
Bouchonville 2014 b	-4.9	9.5	28	-6.7	10.3	26	1.5%	1.80 [-3.50, 7.10]
Cao 2019	-2	12	13	4	9.5	15	0.8%	-6.00 [-14.10, 2.10]
Croymans 2014	-5	7.73	28	-5	15.55	8	0.5%	0.00 [-11.15, 11.15]
Fenkci 2006 a	-5	10	17	-3.3	8.58	9	0.9%	-1.70 [-9.05, 5.65]
Fenkci 2006 b	-7.4	8.58	17	-3.3	8.58	9	1.0%	-4.10 [-11.03, 2.83]
Figueroa 2012	-2	4.47	5	2	4.47	5	1.4%	-4.00 [-9.54, 1.54]
Fritz 2012 a	0.9	7.8	38	-0.3	8	75	2.7%	1.20 [-1.87, 4.27]
Fritz 2012 b	-3.8	9.1	8	0.5	7.3	21	1.0%	-4.30 [-11.34, 2.74]
Fritz 2012 c	-3.3	13.5	9	-1.7	8.7	30	0.6%	-1.60 [-10.95, 7.75]
Goodpaster 2010	-2.33	8.66	67	-1.54	8.62	63	2.8%	-0.79 [-3.76, 2.18]
Gorostegi-Anduaga 2018 a	-3.4	7.43	40	-4.8	8.31	13	1.6%	1.40 [-3.67, 6.47]
Gorostegi-Anduaga 2018 b	-4.9	6.57	42	-4.8	8.31	13	1.7%	-0.10 [-5.03, 4.83]
Gorostegi-Anduaga 2018 c	-4.3	7.83	41	-4.8	8.31	13	1.6%	0.50 [-4.61, 5.61]
Heydary 2013	-5.9	13.6	17	1.6	9.2	17	0.9%	-7.50 [-15.31, 0.31]
Hinderliter 2014	-9.9	5.72	46	-7.5	6.06	46	3.2%	-2.40 [-4.81, 0.01]
Ho 2012 a	0.2	8.34	15	-2.19	7.21	5	0.9%	2.39 [-5.21, 9.99]
Ho 2012 b	-1	9.76	16	-2.19	7.21	5	0.8%	1.19 [-6.74, 9.12]
Ho 2012 c	-2.88	7.42	17	-2.19	7.21	5	1.0%	-0.69 [-7.93, 6.55]
Kadoglou 2010 a	-2.86	4.29	22	0.89	8.48	21	2.1%	-3.75 [-7.80, 0.30]
Kadoglou 2010 b	-7.31	3.35	23	-3.45	4.45	23	3.3%	-3.86 [-6.14, -1.58]
Keating 2015 a	-3.83	6.68	12	-0.17	5.54	4	1.1%	-3.66 [-10.28, 2.96]
Keating 2015 b	-8.05	7.66	12	-0.17	5.54	4	1.0%	-7.88 [-14.83, -0.93]
Keating 2015 c	-1.67	8.3831	12	-0.17	5.54	4	1.0%	-1.50 [-8.71, 5.71]
Kim 2012	-7.54	5.74	15	1.79	3.07	15	2.6%	-9.33 [-12.62, -6.04]
Kozey-Keadle 2014 a	-1.3	8.45	16	-1.8	8.75	8	0.9%	0.50 [-6.84, 7.84]
Kozey-Keadle 2014 b	-3.5	7.66	16	-4	6.25	14	1.7%	0.50 [-4.48, 5.48]
Kucio 2017	-2.9	6.57	15	-0.8	5.48	11	1.8%	-2.10 [-6.74, 2.54]
Larson-Meyer 2009	-4	2.1	12	-2.06	1.71	12	3.9%	-1.94 [-3.47, -0.41]
Masuo 2012	-18	5.67	30	-12	4.58	30	3.1%	-6.00 [-8.61, -3.39]
Meckling 2006 a	-5	8.5	11	-6	12.7	8	0.5%	1.00 [-9.13, 11.13]
Meckling 2006 b	-4	8.8	14	-7	11.2	10	0.8%	3.00 [-5.33, 11.33]
Mendham 2014	-1.5	7.83	11	-1	10.2	10	0.9%	-0.50 [-8.33, 7.33]
Mohr 2014 a	0	9.17	21	1	8.94	10	1.1%	-1.00 [-7.79, 5.79]
Mohr 2014 b	-2	17.75	21	1	8.94	10	0.6%	-3.00 [-12.40, 6.40]
Oh 2018 a	-3.7	8.3	8	1.8	5	8	1.1%	-5.50 [-12.21, 1.21]
Oh 2018 b	-2.3	4.6	10	-1.4	8.6	9	1.2%	-0.90 [-7.20, 5.40]
Plotnikoff 2010	-1.4	7.73	27	0.1	8.44	21	1.8%	-1.50 [-6.14, 3.14]
Pugh 2014	-0.3	5.73	13	-3.1	7.27	8	1.3%	2.80 [-3.12, 8.72]
Ryan 2014	-2	6.08	37	-3	6.32	40	3.0%	1.00 [-1.77, 3.77]
Schroeder 2018 a	-2	3.88	17	0	1.9	6	3.3%	-2.00 [-4.39, 0.39]
Schroeder 2018 b	0	7.77	17	0	1.9	6	2.1%	0.00 [-3.99, 3.99]
Schroeder 2018 c	-4	4.02	18	0	1.9	6	3.2%	-4.00 [-6.40, -1.60]
Shah 2009	-7.8	6.3	9	-3	11.7	9	0.7%	-4.80 [-13.48, 3.88]
Stensvold 2010 a	-4.1	6.25	11	-0.6	2.82	4	1.8%	-3.50 [-8.11, 1.11]
Stensvold 2010 b	0.8	7.54	10	-0.6	2.82	4	1.5%	1.40 [-4.03, 6.83]
Stensvold 2010 c	-1.7	6.69	11	-0.6	2.82	4	1.7%	-1.10 [-5.92, 3.72]
Straznicky 2012	-3.6	6.7	19	-3.2	6	20	2.1%	-0.40 [-4.40, 3.60]
Swift 2012 a	-0.2	6.74	93	-0.48	4.69	45	3.6%	0.28 [-1.66, 2.22]
Swift 2012 b	-0.38	6.74	82	-0.48	4.69	45	3.6%	0.10 [-1.90, 2.10]
Tjonna 2008 a	-6	14.49	8	1	10.25	5	0.3%	-7.00 [-20.47, 6.47]
Tjonna 2008 b	-6	10.39	11	1	10.25	5	0.5%	-7.00 [-17.88, 3.88]
Waib 2011	-0.7	10.29	55	-0.4	9.84	24	1.7%	-0.30 [-5.08, 4.48]
Winding 2017 a	-3	8.18	12	-2	6.24	4	0.9%	-1.00 [-8.67, 6.67]
Winding 2017 b	-1	5	13	-2	6.24	4	1.1%	1.00 [-5.69, 7.69]
**Total (95% CI)**			**1310**			**930**	**100.0%**	**-1.93 [-2.73, -1.13]**

Heterogeneity: Tau² = 3.65; Chi² = 124.12, df = 57 (P < 0.00001); I² = 54%
Test for overall effect: Z = 4.72 (P < 0.00001)

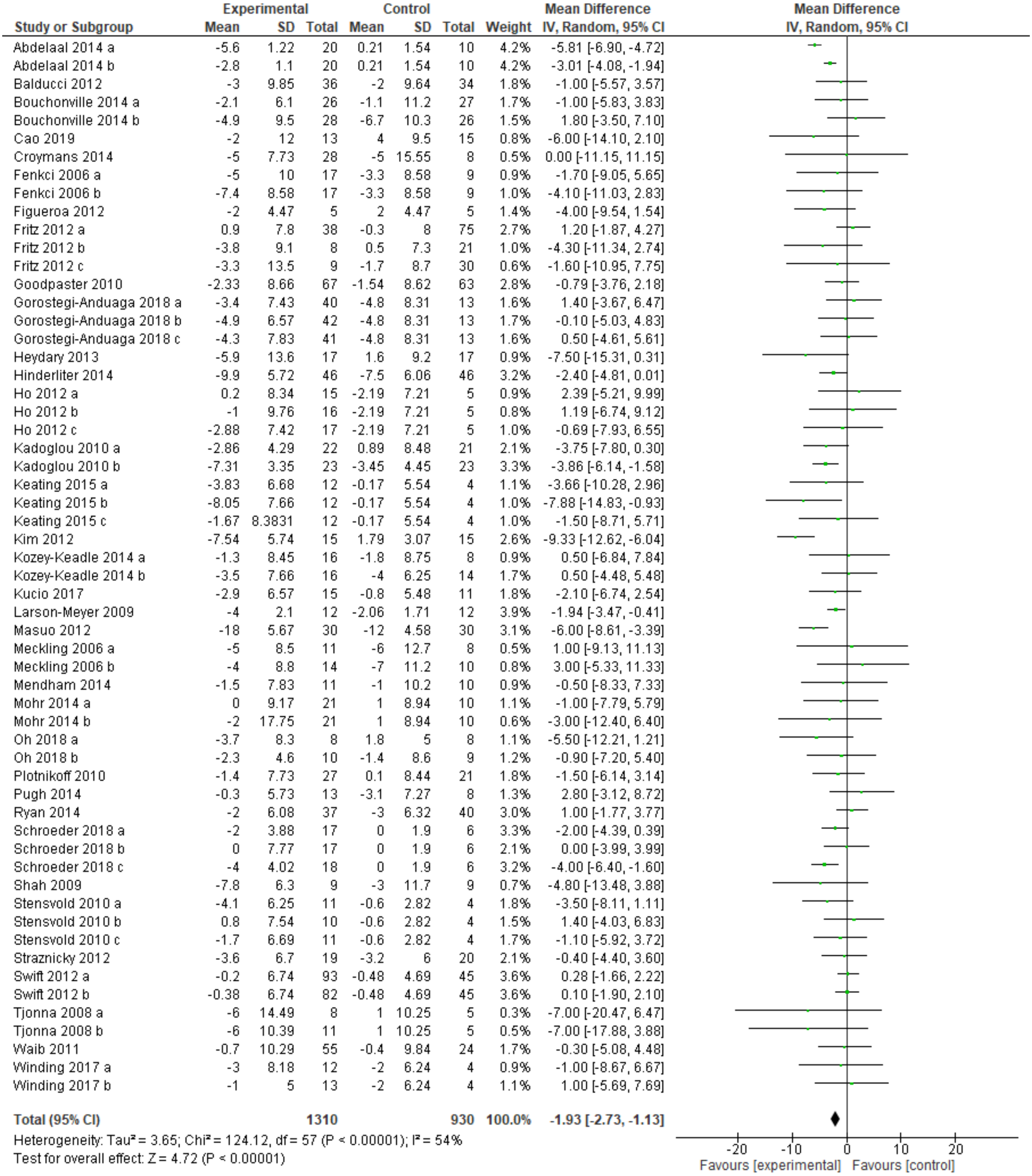

Articles are presented in alphabetical order. Abdelaal 2014 (a): Aerobic exercise; Abdelaal 2014 (b): resistance exercise. Bouchonville 2014 (a): diet+exercise vs. diet; Bouchonville 2014 (b): exercise vs. control. Fenkci (a): aerobic training; Fenkci (b): resistance training. Fritz 2012 (a): normal glucose tolerance; Fritz 2012 (b): impaired glucose tolerance; Fritz 2012 (c): type 2 diabetes. Gorostegi-Anduaga 2018 (a): moderate intensity continuous training; Gorostegi-Anduaga 2018 (b): high volume-high intensity interval training; Gorostegi-Anduaga 2018 (c): low volume-high intensity interval training. Ho 2012 (a): aerobic exercise; Ho 2012 (b): resistance exercise; Ho 2012 (c): combined exercise. Kadoglou 2010 (a): exercise vs. control; Kadoglou 2010 (b): rosiglitazone+exercise vs. rosiglitazone. Keating 2015 (a): high intensity, low volume aerobic exercise; Keating 2015 (b): low intensity, high volume aerobic exercise; Keating (c): low intensity, low volume aerobic exercise. Kozey-

Keadle 2014 (a): exercise vs. control; Kozey-Keadle 2014 (b): exercise+reducing sitting time vs. reducing sitting time. Meckling (a): control diet+exercise vs. control diet; Meckling (b): high protein diet+exercise vs. high protein diet.Mohr 2014 (a): moderate intensity continuous training vs. control; Mohr 2014 (b): high intensity interval training vs. control. Oh 2018 (a): exercise vs. control; Oh 2018 (b): diet+exercise vs. diet. Schroeder 2018 (a): aerobic exercise; Schroeder 2018 (b): resistance exercise; Schroeder 2018 (c): combined exercise. Stensvold 2010 (a): aerobic interval training; Stensvold 2010 (b): combined training; Stensvold 2010 (c): strength training. Swift 2012 (a): higher energy expenditure (12 kcal/Kg/week); Swift 2012 (b): lower energy expenditure (8 kcal/Kg/week). Tjonna 2008 (a): moderate intensity continuous training; Tjonna 2008 (b): aerobic interval training. Winding 2014 (a): endurance training; Winding 2014 (b): high intensity interval training;

Figure S3. Forest plot of the effect of exercise training programmes vs. control on HOMA-IR in adults with overweight or obesity.

	Experimental			Control				Std. Mean Difference
Study or Subgroup	Mean	SD	Total	Mean	SD	Total	Weight	IV, Random, 95% CI
Abdelbasset 2019	-0.8	1.49	16	0.18	1.67	8	2.2%	-0.61 [-1.48, 0.26]
Abdelbasset 2020	-0.8	1.41	15	0.18	1.14	8	2.1%	-0.71 [-1.60, 0.18]
Andersen 2014	-0.9	1.7	10	0.8	1.6	8	1.8%	-0.98 [-1.97, 0.02]
Bouchonville 2014 a	-0.7	1.8	26	-0.3	2.9	27	3.6%	-0.16 [-0.70, 0.38]
Bouchonville 2014 b	-1.7	1.9	28	-1.4	1.8	26	3.6%	-0.16 [-0.69, 0.38]
Cuthbertson 2016	-0.43	1.01	30	0.03	0.79	20	3.4%	-0.49 [-1.06, 0.09]
Fenkci 2006 a	-0.88	1.21	17	0.1	1.9	9	2.3%	-0.64 [-1.47, 0.19]
Fenkci 2006 b	0.15	1.33	17	0.1	1.9	9	2.4%	0.03 [-0.78, 0.84]
Fritz 2012 a	-0.1	0.4	38	0.02	1.8	75	4.5%	-0.08 [-0.47, 0.31]
Fritz 2012 b	-0.1	0.4	8	0.04	1.4	21	2.3%	-0.11 [-0.93, 0.70]
Fritz 2012 c	-0.04	0.4	9	-0.6	2.7	30	2.6%	0.23 [-0.52, 0.98]
Garcia-Unciti 2012	-1.1	1.3	13	-1.4	1.4	12	2.4%	0.22 [-0.57, 1.00]
Goodpaster 2010	-1.33	1.95	67	-1.12	1.99	63	4.8%	-0.11 [-0.45, 0.24]
Hallsworth 2011	-1.3	5.37	11	0.4	2.33	8	2.0%	-0.37 [-1.29, 0.55]
Hallsworth 2015	-0.3	0.49	12	0	0.6	11	2.3%	-0.53 [-1.37, 0.30]
Houghton 2017	-0.4	1.16	12	0.1	0.2419	12	2.3%	-0.58 [-1.40, 0.24]
Johnson 2009	-0.19	2.51	12	0.06	2.69	7	2.0%	-0.09 [-1.03, 0.84]
Kadoglou 2010 a	-0.85	0.85	22	2.12	2.6	21	2.9%	-1.52 [-2.21, -0.84]
Kadoglou 2010 b	-3.25	2.81	23	-1.87	2.44	23	3.3%	-0.52 [-1.10, 0.07]
Kim 2012	-0.07	0.16	15	0.09	1.02	15	2.7%	-0.21 [-0.93, 0.50]
Kim 2014	-0.53	0.88	29	0.2	0.85	10	2.6%	-0.82 [-1.56, -0.08]
Kolahdouzi 2018	-0.48	0.23	13	0.03	0.32	13	2.0%	-1.77 [-2.70, -0.84]
Labrunee 2012	-11.9	67.5	11	-5	46.4	12	2.3%	-0.12 [-0.93, 0.70]
Masuo 2012	-1.1	0.78	14	-0.6	0.44	24	2.9%	-0.84 [-1.52, -0.15]
Mendham 2014	-0.9	2.43	11	1	2.99	10	2.1%	-0.67 [-1.56, 0.21]
Oh 2018 a	-0.9	1.3	12	0.6	2.8	13	2.4%	-0.66 [-1.46, 0.15]
Oh 2018 b	0	0.8	10	0.5	0.9	10	2.1%	-0.56 [-1.46, 0.34]
Pourranjbar 2018	-1.15	0.17	38	-0.01	2.119	42	4.1%	-0.73 [-1.19, -0.28]
Pugh 2014	-0.2	1.15	13	-0.2	0.83	8	2.1%	0.00 [-0.88, 0.88]
Ryan 2014	-0.6	1.6	37	-0.9	1.09	40	4.1%	0.22 [-0.23, 0.67]
Shah 2009	-1.2	1.2	9	-1.5	1.8	9	2.0%	0.19 [-0.74, 1.11]
Straznicky 2012	-0.84	1	19	-1.66	1.44	20	3.1%	0.64 [-0.00, 1.29]
Taghian 2014	-1.19	1.58	10	-0.15	0.24	10	2.0%	-0.88 [-1.81, 0.05]
Waib 2011	-0.7	2.41	55	0.4	1.67	24	3.9%	-0.49 [-0.98, -0.01]
Winding 2017 a	0.3	0.65	12	0	1.25	4	1.5%	0.35 [-0.79, 1.49]
Winding 2017 b	-0.59	1.97	13	0	1.25	4	1.5%	-0.30 [-1.43, 0.82]
Zelber-Sagi 2014	0.37	2.04	33	-0.24	1.75	31	3.9%	0.32 [-0.18, 0.81]
**Total (95% CI)**			**740**			**697**	**100.0%**	**-0.34 [-0.49, -0.18]**

Heterogeneity: Tau² = 0.10; Chi² = 68.66, df = 36 (P = 0.0008); I² = 48%
Test for overall effect: Z = 4.16 (P < 0.0001)

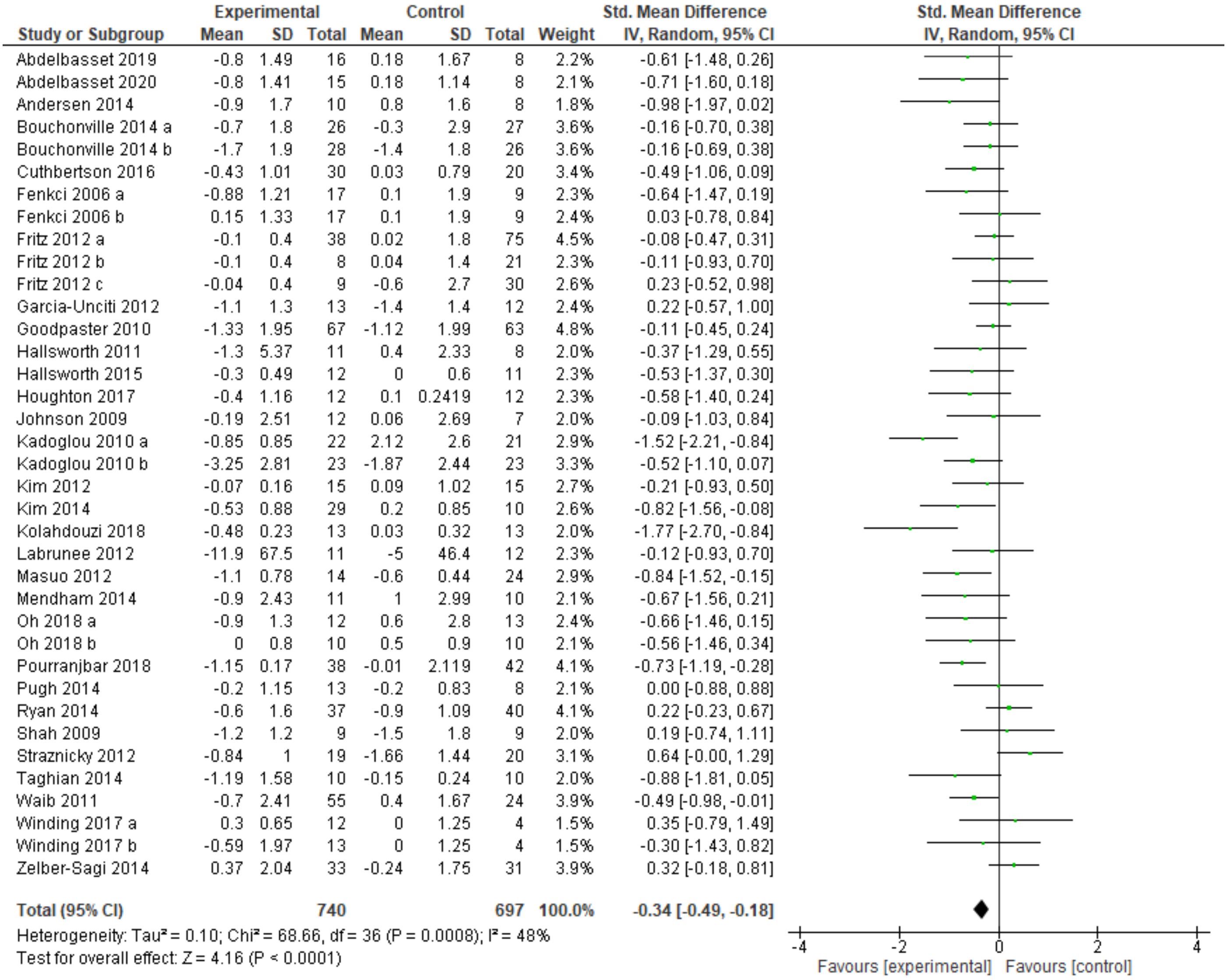

Articles are presented in alphabetical order. Bouchonville 2014 (a): diet+exercise vs. diet; Bouchonville 2014 (b): exercise vs. control. Fenkci 2006 (a): aerobic training; Fenkci (b): resistance training. Fritz 2012 (a): normal glucose tolerance; Fritz 2012 (b): impaired glucose tolerance; Fritz 2012 (c): type 2 diabetes. Kadoglou 2010 (a): exercise vs. control; Kadoglou 2010 (b): rosiglitazone+exercise vs. rosiglitazone. Oh 2018 (a): exercise vs. control; Oh 2018 (b): diet+exercise vs. diet; Winding 2014 (a): endurance training; Winding 2014 (b): high intensity interval training;

Figure S4. Forest plot of the effect of exercise training programmes vs. control on intrahepatic fat in adults with overweight or obesity.

Study or Subgroup	Experimental Mean	Experimental SD	Experimental Total	Control Mean	Control SD	Control Total	Weight	Std. Mean Difference IV, Random, 95% CI
Abdelbasset 2019	-2.3	3.12	16	0.1	2.84	8	4.4%	-0.76 [-1.64, 0.12]
Abdelbasset 2020	-2.4	4.24	15	-0.1	2.81	8	4.5%	-0.58 [-1.46, 0.30]
Cuthbertson 2016	-9.3	10.44	30	-2.5	7.9	20	10.1%	-0.70 [-1.29, -0.12]
Hallsworth 2012	-1.8	1.88	11	0.3	3.63	8	3.8%	-0.73 [-1.68, 0.22]
Hallsworth 2015	-2.8	3.95	12	0.1	5.73	11	4.9%	-0.57 [-1.41, 0.27]
Houghton 2017	-2	2.97	12	1	1.79	12	4.5%	-1.18 [-2.06, -0.30]
Johnson 2009	-1.76	7.8	12	0.26	10.15	7	3.9%	-0.22 [-1.16, 0.71]
Keating 2015 a	-2.62	4.58	12	1.1	8.02	4	2.6%	-0.64 [-1.80, 0.52]
Keating 2015 b	-2.38	6.15	12	1.1	8.02	4	2.6%	-0.50 [-1.65, 0.65]
Keating 2015 c	-0.84	4.31	12	1.1	8.02	4	2.7%	-0.34 [-1.48, 0.80]
Pugh 2014	-8.4	17.68	13	-5	8.82	8	4.4%	-0.22 [-1.10, 0.67]
Shah 2009	-3.8	3.6	9	-3.7	3.3	9	4.0%	-0.03 [-0.95, 0.90]
Sullivan 2012	-2.8	12.7	12	2.67	31.66	6	3.6%	-0.25 [-1.24, 0.73]
Winn 2017 a	-37	35.07	8	17.3	32.4	3	1.5%	-1.44 [-2.96, 0.09]
Winn 2017 b	-20.1	18.66	8	17.3	32.42	3	1.4%	-1.52 [-3.07, 0.02]
Zhang 2016 a	-6.35	6.45	69	-2.2	9.42	37	20.9%	-0.54 [-0.95, -0.14]
Zhang 2016 b	-7.15	6.3	66	-2.2	9.42	37	20.2%	-0.65 [-1.06, -0.24]
**Total (95% CI)**			**329**			**189**	**100.0%**	**-0.59 [-0.78, -0.41]**

Heterogeneity: $Tau^2 = 0.00; Chi^2 = 8.20, df = 16 (P = 0.94); I^2 = 0\%$
Test for overall effect: Z = 6.28 (P < 0.00001)

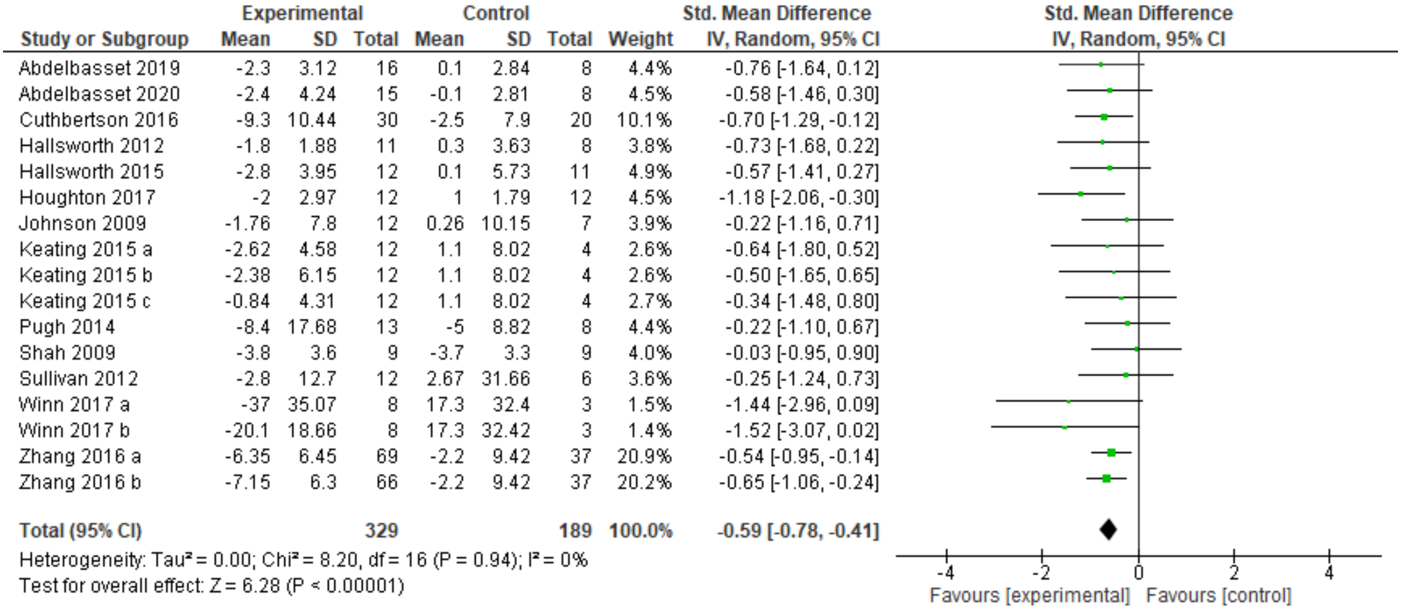

Articles are presented in alphabetical order. Articles are presented in alphabetical order. Keating 2015 (a): high intensity, low volume aerobic exercise; Keating 2015 (b): low intensity, high volume aerobic exercise. Keating (c): low intensity, low volume aerobic exercise. Winn (a): high intensity interval training; Winn (a): moderate intensity continuous training; Zhang (a): moderate intensity training; Zhang (b): vigorous intensity training.

Figure S5. Funnel plot.

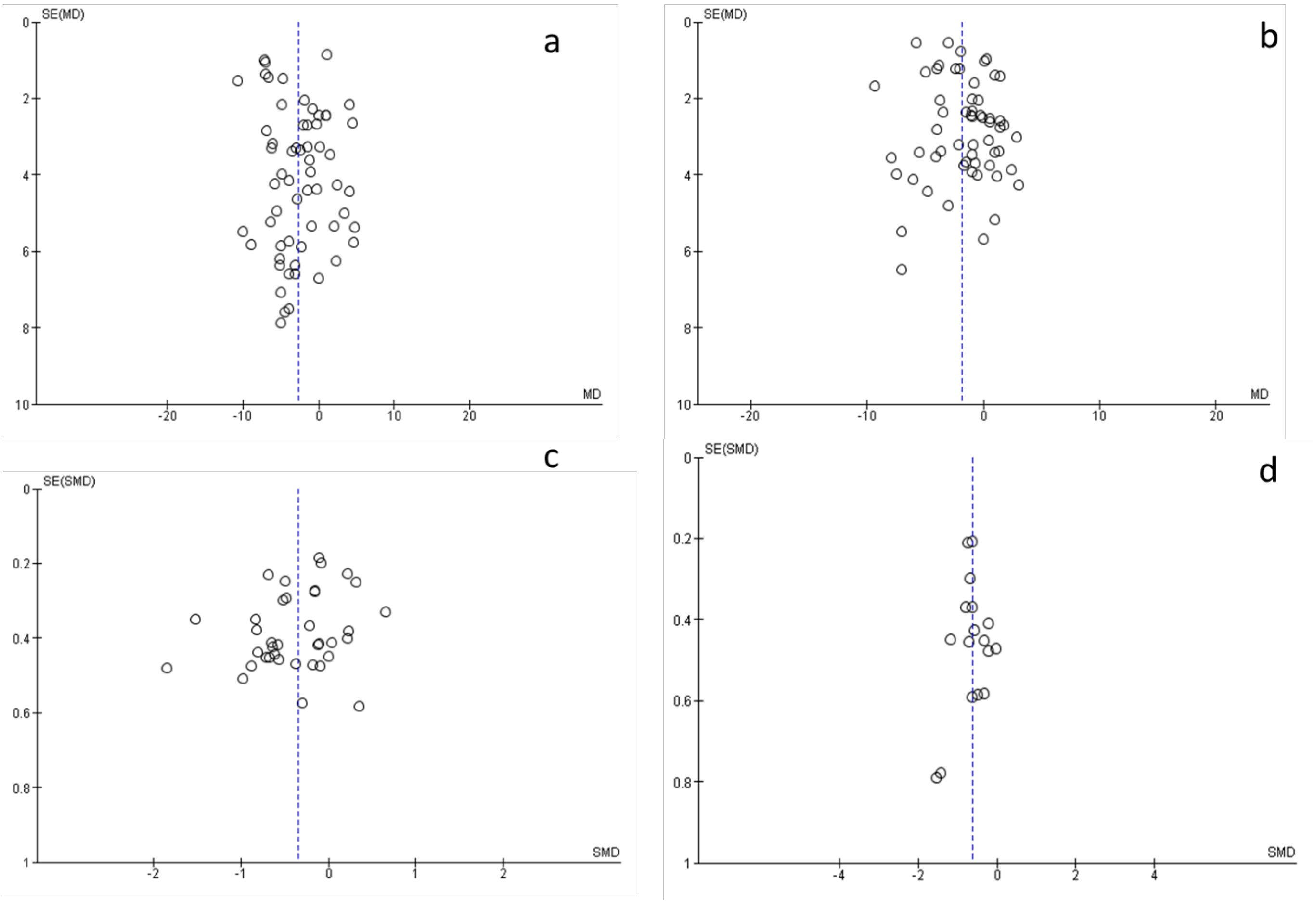

a. Systolic blood pressure; b. Diastolic blood pressure; c. HOMA-IR; d. Intrahepatic fat.

Table S2. Summary of quality assessment of original studies.

**References**	**Criteria**														Total "Yes"	Total "No"	Total "other"	Quality rating
	**1**	2	3	4	5	6	**7**	8	9	10	11	12	13	**14**				
Controlled trials																		
Abdelaal et al, 2014	**Y**	Y	Y	NA	N	Y	**Y**	Y	NR	Y	Y	Y	Y	**N**	10	2	2	Fair
Abdelbasset et al, 2019	**Y**	Y	Y	NA	N	Y	**Y**	Y	Y	Y	Y	Y	Y	**NA**	11	1	2	Good
Abdelbasset et al, 2020	**Y**	Y	Y	Y	N	Y	**Y**	Y	NR	Y	Y	Y	Y	**N**	11	2	1	Fair
Andersen et al, 2014	**Y**	N	N	NA	N	Y	**Y**	Y	Y	Y	Y	N	Y	**N**	8	5	1	Poor
Balducci et al, 2012	**Y**	Y	Y	NA	N	Y	**Y**	Y	NR	Y	Y	N	Y	**N**	9	3	2	Fair
Bouchonville et al, 2014	**Y**	Y	Y	NA	Y	Y	**Y**	Y	Y	Y	Y	Y	Y	**Y**	13	0	1	Good
Cao et al, 2019	**Y**	N	Y	NA	NR	Y	**Y**	Y	NR	Y	Y	N	Y	**N**	8	3	3	Poor
Croymans et al, 2014	**Y**	Y	Y	NA	Y	Y	**Y**	Y	Y	Y	Y	N	Y	**N**	11	2	1	Fair
Cuthbertson et al, 2016	**Y**	Y	Y	NA	N	Y	**N**	Y	Y	Y	Y	N	Y	**N**	9	4	1	Poor
Fenkci et al, 2006	**Y**	NR	NA	NA	NR	Y	**Y**	Y	NR	Y	Y	N	Y	**N**	7	2	5	Fair
Figueroa et al, 2012	**Y**	N	N	NA	NR	Y	**Y**	Y	NR	Y	Y	N	Y	**N**	7	4	3	Poor
Figueroa et al, 2015	**Y**	Y	Y	NA	Y	Y	**Y**	Y	Y	Y	Y	N	Y	**N**	10	2	2	Fair
Fritz et al, 2012	**Y**	Y	Y	NA	NR	Y	**Y**	Y	Y	Y	Y	Y	Y	**Y**	12	0	2	Good
Garcia-Unciti et al 2012	**Y**	N	NR	NA	NR	Y	**Y**	Y	Y	Y	Y	N	Y	**N**	8	3	3	Poor
Goodpaster et al, 2010	**Y**	Y	NA	NR	Y	Y	**Y**	Y	Y	Y	Y	N	Y	**Y**	11	1	2	Good
Gorostegi-Anduaga et al, 2018	**Y**	Y	Y	NA	Y	Y	**Y**	Y	Y	Y	Y	Y	Y	**Y**	13	0	1	Good
Hallsworth et al, 2011	**Y**	Y	NR	NA	Y	Y	**Y**	Y	Y	Y	Y	N	Y	**NA**	10	1	3	Good
Hallsworth et al, 2015	**Y**	Y	N	NA	N	Y	**Y**	Y	NR	Y	Y	Y	Y	**N**	9	3	2	Fair

Heydary et al, 2013	**Y**	Y	Y	NA	Y	Y	**Y**	Y	Y	Y	Y	N	Y	**N**	11	2	1	Fair
Hinderliter et al, 2014	**Y**	Y	Y	NA	N	Y	**Y**	Y	NR	Y	Y	N	Y	**Y**	10	1	3	Good
Ho et al, 2012	**Y**	Y	Y	NA	N	Y	**Y**	Y	CD	Y	Y	N	Y	**N**	9	3	2	Fair
Houghton et al, 2017	**Y**	Y	Y	NA	N	Y	**Y**	Y	NR	Y	Y	Y	Y	**N**	10	2	2	Fair
Johnson et al, 2009	**Y**	Y	NR	NA	Y	Y	**Y**	N	Y	Y	Y	N	Y	**N**	9	3	2	Fair
Kadoglou et al, 2010	**Y**	Y	Y	NA	NR	Y	**Y**	Y	Y	Y	Y	N	Y	**N**	10	2	2	Fair
Keating et al, 2015	**Y**	Y	Y	Y	Y	Y	**Y**	Y	Y	Y	Y	N	Y	**Y**	13	1	0	Good
Kim JW et al, 2012	**Y**	N	NR	NA	NR	Y	**Y**	Y	NR	Y	Y	N	Y	**N**	7	3	4	Poor
Kim YS et al, 2014	**Y**	N	NR	NA	NR	Y	**Y**	Y	NR	Y	Y	N	Y	**N**	7	3	4	Poor
Kolahdouzi et al 2018	**Y**	N	NR	NA	NR	Y	**Y**	Y	NR	Y	Y	N	Y	**N**	7	3	4	Poor
Kozey-Keadle et al, 2014	**Y**	Y	Y	NA	N	Y	**Y**	Y	Y	Y	Y	Y	Y	**N**	11	2	1	Fair
Kucio et al, 2017	**Y**	N	N	NA	N	Y	**Y**	Y	Y	Y	Y	N	Y	**N**	8	5	1	Poor
Labrunée et al, 2012	**Y**	N	NR	NA	NR	Y	**Y**	Y	NR	N	Y	N	Y	**N**	6	4	4	Poor
Larson-Meyer et al, 2009	**Y**	CD	NR	NA	NR	Y	**Y**	Y	NR	Y	Y	N	Y	**N**	8	2	4	Poor
Masuo et al, 2014	**Y**	Y	NR	NA	NR	Y	**Y**	Y	Y	Y	Y	N	Y	**N**	9	2	3	Fair
Meckling et al, 2007	**Y**	N	NR	NA	NR	Y	**N**	Y	NR	Y	Y	Y	Y	**N**	7	3	4	Poor
Mendham et al, 2015	**Y**	Y	N	NA	N	Y	**Y**	Y	N	Y	Y	N	Y	**N**	8	5	1	Poor
Mohr et al, 2014	**Y**	N	NR	NA	NR	Y	**Y**	Y	Y	Y	N	N	Y	**N**	7	4	3	Poor
Oh et al, 2017	**Y**	Y	Y	N	Y	Y	**Y**	N	Y	Y	Y	N	Y	**N**	10	4	0	Fair
Plotnikoff et al, 2010	**Y**	Y	Y	NA	Y	Y	**Y**	Y	N	Y	Y	Y	Y	**Y**	12	1	1	Good
Pugh et al, 2014	**Y**	Y	Y	NA	N	Y	**N**	Y	Y	Y	Y	N	Y	**N**	9	4	1	Poor
Pourranjbar et al, 2018	**Y**	N	NR	NA	NR	Y	**Y**	Y	NR	Y	Y	Y	Y	**N**	8	2	4	Poor
Ryan et al, 2014	**Y**	N	NR	NA	NR	Y	**N**	Y	Y	Y	Y	N	Y	**N**	7	4	3	Poor
Schroeder et al, 2018	**Y**	Y	Y	NA	Y	Y	**Y**	Y	Y	Y	Y	N	Y	**Y**	12	1	1	Good
Shah et al, 2009	**Y**	Y	NR	NA	Y	Y	**Y**	Y	Y	Y	Y	Y	Y	**N**	11	1	2	Fair
Stensvold et al, 2010	**Y**	Y	Y	NA	NR	Y	**Y**	Y	Y	N	Y	N	Y	**N**	9	3	2	Fair
Straznicky et al, 2012	**Y**	Y	N	NA	N	Y	**Y**	Y	CD	Y	Y	N	Y	**N**	8	4	2	Poor
Sullivan et al, 2012	**Y**	Y	N	NA	N	Y	**N**	Y	CD	Y	Y	Y	Y	**N**	8	4	2	Poor
Swift et al, 2012	**Y**	Y	Y	Y	Y	Y	**Y**	Y	Y	Y	Y	N	Y	**N**	12	2	0	Fair
Taghian et al, 2014	**Y**	N	NR	NA	NR	N	**Y**	Y	NR	Y	Y	N	Y	**N**	6	4	4	Poor
Tjønna et al, 2008	**Y**	N	N	NA	Y	Y	**Y**	Y	Y	Y	Y	Y	Y	**N**	10	3	1	Fair
Waib et al, 2010	**Y**	N	N	NA	N	Y	**Y**	Y	Y	Y	Y	Y	Y	**CD**	10	2	2	Fair
Winding et al, 2017	**Y**	N	Y	NA	NR	Y	**N**	NR	NR	Y	Y	N	Y	**N**	6	4	4	Poor
Winn et al, 2017	**Y**	Y	N	NA	N	N	**Y**	Y	CD	Y	Y	N	Y	**N**	7	5	2	Poor
Zelber-Sagi et al, 2014	**Y**	N	Y	NA	Y	Y	**N**	Y	NR	Y	Y	Y	Y	**N**	10	3	1	Poor
Zhang et al 2016	**Y**	Y	Y	Y	Y	Y	**Y**	Y	Y	Y	Y	Y	Y	**Y**	14	0	0	Good

Criteria controlled trials: (1) Randomized study; (2) Adequate randomization method; (3) Treatment allocation concealment; (4) Blinding treatment assignment; (5) Blinding outcome assessors; (6) Similar baseline characteristics; (7) Drop-out rate <20%; (8) Differential drop-out rate between groups <15%; (9) High adherence; (10) Similar background treatments; (11) Valid and reliable outcome measures; (12) Sample size justification; (13) Pre-specified outcomes/subgroups; (14) All randomized participants analysed (ITT analysis). In bold text are reported fatal flaws. Y: yes; N=no; NR: not reported; NA: not applicable; CD: cannot determine.

Table 3. Characteristics of original studies

Reference	Study design	Population	Intervention	Comparison	Outcomes	Follow-up duration
Abdelaal et al, 2015[1]	RCT	Resistance training (N=20)<br>Age: 52.2±3 y<br>BMI: 34.76±1.14 Kg/m^2<br>% female: 55%<br>Comorbidities: HTN, T2DM.<br><br>Aerobic training (N=20)<br>Age: 53±3.5 y<br>BMI: 34.6±1.13 Kg/m^2<br>% female: 60%<br>Comorbidities: HTN, T2DM.<br><br>Control (N=19)<br>Age: 52±3.3 y<br>BMI: 34.1±1.17 Kg/m^2<br>% female: 53%<br>Comorbidities: HTN, T2DM.	Resistance training<br>- Program duration: 12 weeks<br>- Number of sessions: 3/week<br>- Type of training 60—65% of 1RM was used during the first month; 65—70% 1RM during the second month; 70—75% 1RM during the 3rd month. 2 sets of 10 repetitions of 10 different exercises during the 1st month; 3 sets of the 10 repetitions of 10 different exercises during the 2nd and 3rd months. 90—120 s rest between each 2 successive sets.<br>- Supervision: fully<br><br>Aerobic training<br>- Program duration: 12 weeks<br>- Number of sessions: 3/week<br>- Type of training: treadmill; rate of perceived exertion between 12 and 14 on Borg's score scale. Training began with 20—35 min duration on 60—65% of HRmax intensity during the first month. 35—40 min duration on 65—70% of HRmax intensity during the second month, and then 40—50 min duration on 70—75% of HRmax intensity during the third month.<br>- Supervision: fully<br><br>Sufficient warm up and cool down (about 10—15 min) in the form of stretching of major muscle groups, flexibility and active movements of trunk, upper and lower limbs, deep breathing exercises and pace walking at low intensity (30—40% of HRmax achieved from the treadmill exercise testing or at 10—12 grade on Borge's score scale) were performed before and after every training session.<br><br>Controls<br>Usual care.	- Resistance vs. control<br>- Aerobic vs. control	- Systolic blood pressure<br>- Diastolic blood pressure	Short term: 12 weeks.
Abdelbasset et al, 2019[2]	RCT	HIIT (N=16)<br>Age: 54.4±5.8 y<br>BMI: 36.3±4.5 Kg/m^2<br>% female: 38%<br>Comorbidities: T2DM, dyslipidaemia, NAFLD<br><br>Control (N=16)<br>Age: 55.2±4.3 y<br>BMI 35.9±5.3 Kg/m^2<br>% female: 44%<br>Comorbidities: T2DM, dyslipidaemia, NAFLD.	HIIT<br>- Program duration: 8 weeks<br>- Number of sessions: 3/week<br>- Type of training 40 minutes HIIT, on a cycle-ergometer 5-minute warm-up which involved cycling exercise without resistance followed by 3 sets of 4-min cycling sessions at 80% to 85% of the VO2max with 2-minute interval at 50% of the VO2max between sets<br>- Supervision: no<br><br>Control group<br>- Pharmacological treatment for comorbidities only. No change in lifestyle.	- HIIT vs. control	- Intrahepatic fat	Short term: 8 weeks
Abdelbasset et al 2020[3]	RCT	MICT (N=15)<br>Age: 54.9±4.7 y<br>BMI: 36.7±3.4 Kg/m^2<br>% female: 46.7%<br>Comorbidities: T2DM, dyslipidaemia, NAFLD.	MICT<br>- Program duration: 8weeks<br>- Number of sessions: 3/week<br>- Type of training: 40–50 minutes. 5-minute warming-up followed by a cycling with continuous intensity at 60–70% of the maximum heart rate (max HR) and the exercise program ended with 5-minute cooling- down.	- MICT vs control	- Intrahepatic fat	Short term: 8 weeks

	RCT	Control (N=16)<br>Age: 55.2±4.3 y<br>BMI 35.9±5.3 Kg/m^2<br>% female: 44%<br>Comorbidities: T2DM, dyslipidaemia, NAFLD	- Supervised: no<br><br>Control group<br>No change in sedentary lifestyle and general advices.			
Andersen et al, 2014[4]	RCT	Exercise (N=10)<br>Age: 50.6±7.1 y<br>BMI: 30.4±1 Kg/m^2<br>Only males.<br>Comorbidities: T2DM.<br><br>Control group (N=8)<br>Age: 48.7±9.2 y<br>BMI 30.4±2.2 Kg/m^2<br>Only males<br>Comorbidities: T2DM.	Exercise<br>- Program duration: 24 weeks<br>- Number of sessions: 2/week<br>- Type of training: 60 minutes of football training (four-a-side, five-a-side, six-a-side), played on a 20m-wide and 40m-long indoor court. 10-minute games interspersed with 2 min passive rest.<br>- Supervised: fully<br><br>Control group<br>- No change in sedentary lifestyle.	- Exercise vs. control	- HOMA-IR	Intermediate term: 24 weeks
Balducci et al, 2012[5]	RCT	Exercise (N=36)<br>Age: 59.6±8.7 y<br>BMI: 31.8±5.3 Kg/m^2<br>%females: 43%<br>Comorbidities: T2DM, MetS.<br><br>Control group: (N=34)<br>Age: 61.6±7.8 y<br>BMI 30.7±4.1 Kg/m^2<br>%females: 47%<br>Comorbidities: T2DM, MetS.	Exercise<br>- Program duration: 48 weeks<br>- Number of sessions: 2/week<br>- Type of training: Each session lasted 75 min and included aerobic exercise plus four resistance exercises.<br>- Supervised: fully<br><br>Control group<br>- Counseling as part of standard care.	- Exercise vs. control	- Systolic blood pressure<br>- Diastolic blood pressure	Long term: 48 weeks.
Bouchonville et al, 2014[6]	RCT	Exercise (N=26)<br>Age: 70±4 y<br>BMI: 36.9±5.4 Kg/m^2<br>%females: 61%<br>Comorbidities: MetS.<br><br>Control (N=27)<br>Age: 64±9 y<br>BMI 37.3±4.7 Kg/m^2<br>%females: 67%<br>Comorbidities: MetS.<br><br>Diet + exercise: (N=28)<br>Age: 70±4 y<br>BMI 37.2±5.4 Kg/m^2<br>%females: 57%<br>Comorbidities: MetS.<br><br>Diet (N=26)<br>Age: 70±4 y<br>BMI 37.2±4.5 Kg/m^2<br>%females: 65%<br>Comorbidities: MetS.	Exercise<br>- Program duration: 24 weeks<br>- Number of sessions: 2/week<br>- Type of training: 90 min duration (15-min flexibility exercise, 30-min aerobic exercise, 30-min progressive resistance training and 15-min balance exercises) Aerobic exercises: treadmill, stationary cycling and stair climbing. 70–85% of peak heart rate. Resistance training: weight-lifting machines and consisted of nine upper and lower extremity exercises. Initially performed 1–2 sets of 8–12 repetitions at 65% of the one-repetition maximum. The number of repetitions was then decreased to 6–8 repetitions per set and resistance was increased to 70–85% of the one-repetition maximum.<br>- Supervised: fully<br><br>Control<br>- Counseling as part of standard care.<br><br>Diet<br>- Balanced diet that provided a deficit of 500–750 kcal per day from daily energy requirement.	- Exercise vs. control<br>- Diet+exercise vs. Diet	- HOMA-IR<br>- Systolic blood pressure<br>- Diastolic blood pressure	Long term:48 weeks

Cao et al, 2018[7]	RCT	Exercise (N=13)<br>Age: 63.8±5.9 y<br>BMI 28±2.9 Kg/m^2<br>Only females<br>Comorbidities: HTN.<br><br>Control (N=15)<br>Age: 64±4.6 y<br>BMI 26.4±1.4 Kg/m^2<br>Only females.<br>Comorbidities: HTN.	Exercise<br>- Program duration: 12 weeks<br>- Number of sessions: 2/week<br>- Type of training: 10-minute warm-up; 20-40 minutes of walking or jogging with the intensity controlled at the individualized FATmax HR. The exercise time was 20 minutes in week 1, 30 minutes in weeks 2-4, and 40 minutes in weeks 5-12. Short breaks of 1-2 minutes were allowed; and finally, there was a 10-minute cool-down.<br>- Supervised: fully<br><br>Control<br>No exercise.	- Exercise vs. control	- Systolic blood pressure<br>- Diastolic blood pressure	Short term: 12 weeks.
Croymans et al, 2014[8]	RCT	Exercise (N=28)*<br>Age: 21.5 (20-23) y<br>BMI: 30.9 (29.7-32.7) Kg/m^2<br>Only males<br>Comorbidities: none.<br><br>Control (N=8)*<br>Age: 22 (20.8-22.8) y<br>BMI 33.6 (31.2-34.7) Kg/m^2<br>Only males<br>Comorbidities: none.	Exercise<br>- Program duration: 12 weeks<br>- Number of sessions: 3/week<br>- Type of training: weeks 1–2: two sets of exercises, with 12–15 repetitions at 100% of their approximated 12–15 RM. Weeks 3–7: 3 sets, with 8–12 repetitions at 100% of their 8–12 RM. Weeks 8–12: 6–8 repetitions, at 100% of their 6–8 RM.<br>- Supervised: fully<br><br>Control group (N=8)<br>- normal ad libitum diets and the normal activities of daily life	- Exercise vs. Control	- Systolic blood pressure<br>- Diastolic blood pressure	Short term: 12 weeks
Cuthbertson et al 2016.[9]	RCT	Exercise (N=30)*<br>Age: 50 (46, 58) y<br>BMI: 30.7 (29.2,32.9) kg/m^2<br>% female: 23%<br>Comorbidities: HTN, dyslipidaemia, NAFLD.<br><br>Control (N=20)*<br>Age: 52 years (46, 59) y<br>BMI: 29.7 (28.0, 33.8) kg/m^2<br>% female: 20%<br>Comorbidities: HTN, dyslipidaemia, NAFLD.	Exercise<br>- Program duration: 16 weeks<br>- Number of sessions: 3-5/week<br>- Type of training: 30 min moderate (30% HRR) aerobic exercise (treadmill, cross-trainer, bike ergometer, rower) progressing weekly based on HR responses (5/week 45 min at 60% HRR by week 12).<br>- Supervision: fully<br><br>Control<br>- General advices.	- Exercise vs control	- Intrahepatic fat	Short term: 16 weeks
Fenkci et al, 2006[10]	RCT	Aerobic exercise (N=17)<br>Age: 41.7± 6.9 y<br>BMI: 35.6±5.6 kg/m^2<br>Only females<br>Comorbidities: none.<br><br>Resistance exercise (N=17)<br>Age: 44±10.2 y<br>BMI: 34.3±3.63 kg/m^2<br>Only females<br>Comorbidities: none.<br><br>Control (N=17)<br>Age: 43.8± 7.4 y<br>BMI: 35.9±4.1 kg/m^2<br>Only females	Aerobic Exercise<br>- Program duration: 12 weeks<br>- Number of sessions: 3-5/week<br>- Type of training: walk briskly for 15 min and then exercise on a stationary leg cycle ergometer; target heart rate range 50%-85% HRR. Patients performed exercises in their target heart rate ranges: 1st mo—3 d a week for 12 to 15 min; 2nd mo—4 d a wk for 20 to 30 min; 3rd mo—5 d a week for 30 to 45 min.<br>- Supervision: not reported<br><br>Resistance Exercise<br>- Program duration: 12 weeks<br>- Number of sessions: 3/week<br>- Type of training: Six stations were used; first week: 1 set (10 repetitions) of lifting 40% to 60% weight with 1-RM; second week: 2 sets; third week: 3 sets of lifting the same weight. During the fourth and twelfth weeks, patients lifted 75% to 80% weight with 1-	- Aerobic exercise vs. control.<br>- Resistance exercise vs. control.	- Systolic blood pressure<br>- Diastolic blood pressure<br>- HOMA-IR	Short term: 12 weeks.

		Comorbidities: none.	RM for 3 sets. A rest of 15 to 30 sec between sets was provided. In both groups, flexibility exercises were performed before and after each exercise session.<br>- Supervision: not reported<br><br>Control<br>No exercise program.			
Figueroa et al, 2012[11]	RCT	Overall<br>Age 22.4±1.8 y<br>BMI, 29.9±0.8 kg/m^2<br><br>Exercise (N=5)<br>Only females.<br>Comorbidities: none.<br><br>Control (N=5)<br>Only females.<br>Comorbidities: none.	Intervention group<br>- Program duration: 6 weeks<br>- Number of sessions: 3/week<br>- Type of training: leg exercises standing on a Whole Body Vibration platform. Dynamic and static semi-squats with a 120 knee angle, wide-stand semi-squat and calf-raises.<br>- Supervised: fully<br><br>Control group<br>- No exercise	- Exercise vs. Control	- Systolic blood pressure<br>- Diastolic blood pressure	Short term: 6 weeks.
Figueroa et al, 2015[12]	RCT	Exercise normal ankle blood pressure (N=12)**<br>Age: 58±1 y<br>BMI: 34.6±0.9 kg/m^2<br>Only females.<br>Comorbidities: pre-HTN or HTN.<br><br>Exercise-high ankle blood pressure (N=12)**<br>Age: 56±1 y<br>BMI: 33.7±1.5 kg/m^2<br>Only females.<br>Comorbidities: pre-HTN or HTN.<br><br>Control group (N=12)**<br>Age: 58±1 y<br>BMI: 32.2±2.1 kg/m^2<br>Only females.<br>Comorbidities: pre-HTN or HTN.	Exercise<br>- Program duration: 12 weeks<br>- Number of sessions: 3/week<br>- Type of training: one to six sets of 4 leg exercises while standing on a WBV training platform. Exercises included unloaded dynamic and static squats with a 90- and 120-knee angle, wide-stand semisquats, and calf raises. Duration of the sets and interset recovery time were progressively increased (30-60 s) and decreased (60-30 s), respectively.<br>- Supervised: fully<br><br>Control<br>- No exercise	- Exercise high blood pressure vs. control<br>- Exercise normal blood pressure vs. control	- Systolic blood pressure	Short term: 12 weeks
Fritz et al, 2012[13]	RCT	<u>Normal glucose tolerance</u><br>Exercise (N=53)<br>Age: 59.4±5.4 y<br>BMI: 29.6±3.8 kg/m^2<br>% females: 62.2<br>Comorbidities: HTN, dyslipidaemia.<br><br>Control (N=75)<br>Age: 59.3±5.9 y<br>BMI: 29.3±2.7 kg/m^2<br>% females: 64<br>Comorbidities: HTN, dyslipidaemia.<br><br><u>Impaired glucose tolerance</u><br>Exercise (N=14)	Exercise<br>- Program duration: 16 weeks<br>- Number of sessions: 5h/week<br>- Type of training: Nordic walking at moderate intensity (slight shortness of breath and perspiration).<br>- Supervised: partially.<br><br>Control<br>- No exercise	- Exercise vs. control	- HOMA-IR.<br>- Systolic blood pressure<br>- Diastolic blood pressure	Short term: 16 weeks.

		Age: 59.1±6.2 y<br>BMI: 32±5.2 kg/m^2<br>% females: 64.2<br>Comorbidities: HTN, dyslipidaemia.<br><br>Control (N=21)<br>Age: 61.8±3.4 y<br>BMI: 30.8±3.5 kg/m^2<br>% females: 52.4<br>Comorbidities: HTN, dyslipidaemia.<br><br><u>Type 2 Diabetes</u><br>Exercise (N=20)<br>Age: 61.4±4.6 y<br>BMI: 31.7±5.2 kg/m^2<br>% females: 35<br>Comorbidities: HTN, dyslipidaemia.<br><br>Control (N=30)<br>Age: 61±4.7<br>BMI: 31.1±3.9<br>% females: 50<br>Comorbidities: HTN, dyslipidaemia.				
Garcia-Unciti et al, 2012[14]	RCT	Diet+Exercise (N=13)<br>Age: 48.6±6.4 y<br>BMI: 35±3.1 Kg/m^2<br>Only females.<br>Comorbidities: None<br><br>Diet (N=12)<br>Age: 51.4±5.5 y<br>BMI: 34.6±3.4 Kg/m^2<br>Only females.<br>Comorbidities: none.	Exercise<br>- Program duration: 16 weeks<br>- Number of sessions: 2/week<br>- Type of training: dynamic resistance exercise for 45–60 min per session (2 exercises for the leg extensor muscles, 1 exercise for the arm extensor muscle and 4–5 exercises for the main muscle groups of the body). During the first 8 weeks: loads of 50–70% of the individual 1-RM, and during the last 8 weeks 70–80% of the maximum. In addition, from week 8 to week 16 the subjects performed a part (20%) of the leg extensor and bench press sets with loads ranging from 30 to 50% of the maximum.<br>- Supervised: fully.<br><br>Diet<br>- No exercise<br>- Caloric restriction of 500 kcal/day<br><br>During the 16 weeks of the study the subjects maintained their customary recreational physical activities (e.g. walking).	- Diet +Exercise vs. diet	- HOMA-IR	Short term: 16 weeks
Goodpaster et al, 2010[15]	RCT	Exercise +Diet (N=67)<br>Age: 46.1±6.5 y<br>BMI: 43.5±4.8 Kg/m^2<br>% females: 85.1<br>Comorbidities: HTN, MetS.<br><br>Diet (N=63)<br>Age: 47.5±6.2 y<br>BMI: 43.7±5.9 Kg/m^2<br>% females: 92.1<br>Comorbidities: HTN, MetS.	Exercise<br>- Program duration: 24 weeks<br>- Number of sessions: 5/week<br>- Type of training: Moderate- intensity physical activity, similar in intensity to brisk walking, was prescribed and progressed to 60 minutes. Were provided with a pedometer and step goals of more than 10 000 steps per day.<br>- diet that result in a sustained 8%to10% weight loss in 12 months. Energy intake was reduced to1200 to2100kcal/d based on initial body weight.<br>- Supervised: partially.<br><br>Diet<br>- No exercise	- Diet+Exercise vs. diet	- HOMA-IR.<br>- Systolic blood pressure<br>- Diastolic blood pressure	Intermediate term: 24 weeks.

			- diet that result in a sustained 8%to10% weight loss in 12months. Energy intake was reduced to1200 to2100kcal/d based on initial body weight.			
Gorostegi-Anduaga et al, 2018[16]	RCT	Diet+High volume MICT (N=40)<br>Age: 54.7±7.6 y<br>BMI: 32.2±4.4 kg/m²<br>% females: 33.3<br>Comorbidities: HTN, MetS, T2DM.<br><br>Diet+High volume HIIT (N=42)<br>Age: 53.5±9.1 y<br>BMI: 31.2±3.6 kg/m²<br>% females: 27.3<br>Comorbidities: HTN, MetS, T2DM.<br><br>Diet+Low volume HIIT (N=41)<br>Age: 54.7±8.8 y<br>BMI: 32±4.6 kg/m²<br>% females: 31.8<br>Comorbidities: HTN, MetS, T2DM.<br><br>Control (N=40)<br>Age: 53.1±8.3 y<br>BMI: 31.9±4.6 kg/m²<br>% females: 33.3<br>Comorbidities: HTN, MetS, T2DM.	High volume MICT<br>- Program duration: 16 weeks<br>- Number of sessions: 2/week<br>- Type of training: 5–10-minute warm-up and a 10-minute cool-down.Aerobic exercises, i.e. one day of the week on the treadmill, and the second one on the bike. The high-volume MICT group performed 45 minutes aerobic exercise at 65% of VO2peak.<br>- A hypocaloric and controlled sodium diet (3–6 g/day) was prescribed for each participant.<br>- Supervised: fully.<br><br>High volume HIIT<br>- Program duration: 24 weeks<br>- Number of sessions: 5/week<br>- Type of training: 5–10-minute warm-up and a 10-minute cool-down. One day on the treadmill (4x4 minutes at 90% of VO2peak and 29 minutes at 65% of VO2peak), and one day on the exercise bike (18x30 seconds at 90% of VO2peak and 36 minutes at 65% of VO2peak).<br>- A hypocaloric and controlled sodium diet (3–6 g/day) was prescribed for each participant.<br>- Supervised: fully.<br><br>Low volume HIIT<br>- Program duration: 24 weeks<br>- Number of sessions: 5/week<br>- Type of training: 5–10-minute warm-up and a 10-minute cool-down. One day on the treadmill (2x4 minutes at 90% of VO2peak and 12 minutes at 65% of VO2peak), and one day on the exercise bike (9x30 seconds at 90% of VO2peak and 15:30 minutes at 65% of VO2peak).<br>- A hypocaloric and controlled sodium diet (3–6 g/day) was prescribed for each participant<br>- Supervised: fully.<br><br>Diet<br>- No exercise<br>- A hypocaloric and controlled sodium diet (3–6 g/day) was prescribed for each participant	- Diet+exercise vs. Diet	- Systolic blood pressure<br>- Diastolic blood pressure	Short term: 16 weeks.
Hallsworth K et al 2011[17]	RCT	Exercise (N=11)<br>Age: 52±13.3; range 33-72 y<br>BMI: 32.3 (4.9) kg/m²<br>% female: nr<br>Comorbidities: NAFLD<br><br>Standard care (N=8)<br>Age: 62±7.4; range 51-71 years<br>BMI: 32.3 ± 4.8 kg/m²<br>% female: nr<br>Comorbidities: NAFLD	Exercise<br>- Program duration: 8 weeks<br>- Number of sessions: 3/week<br>- Type of training: 45 and 60 min and consisted of a 10 min warm-up at 60% maximum heart rate on a cycle ergometer followed by resistance exercise done as a circuit, ending with warm-up. Initially, participants did two circuits using 50% of their one repetition maximum, progressing to three circuits, using a minimum 70% of their one repetition maximum by week 7.<br>- Supervision: partially	- Exercise vs usual care	- Intrahepatic fat	Short term: 8 weeks
Hallsworth K et al 2015[18]	RCT	HIIT (N=12)<br>Age: 54±10 y<br>BMI: 31±4 kg/m²	HIIT<br>Program duration: 12 weeks<br>- Number of sessions: 3/week	- HIIT vs usual care	- Intrahepatic fat.	Short term: 12 weeks

		% female: nr<br>Comorbidities: NAFLD<br><br>Standard care (N=11).<br>Age: 52±12 y<br>BMI: 31±5 kg/m^2<br>% female: nr<br>Comorbidities: NAFLD	- Type of training: a cycle ergometer-based HIIT. 5-min warm up; 5 intervals of cycling at an RPE of 16–17 ('very hard') interspersed with 3-min recovery periods and followed by a 3-min cool down after the last interval. Each interval was 2-min long in the first week with 10 s added per week, so that intervals were 3 min and 50 s long by week 12. Sessions therefore lasted 30–40 min. Recovery periods: 90 s of passive recovery, 60 s of light band resisted upper body exercise and 15 s each to transition off and on the ergometer.<br>- Supervision: no.<br><br>Usual care<br>Any prescription medication and going for regular monitoring of their condition(s) with their normal general practitioner and/or consultant(s).			
Heydary et al, 2013[19]	RCT	Exercise (N=17)<br>Age: 24.4±4.7 y<br>BMI: 28.4±0.6 kg/m^2<br>Only males<br>Comorbidities: none<br><br>Control (N=17)<br>Age: 25.2±4 y<br>BMI: 29.2±0.9 kg/m^2<br>Only males<br>Comorbidities: none	Exercise<br>- Program duration: 12 weeks<br>- Number of sessions: 3/week<br>- Type of training: cycle at a workload of 80–90% of their age-predicted maximum HR with a pedal cadence between 120 and 130 rpm. Each exercise session consisted of a 5-min warm-up, 20-min of 8 s sprint and 12 s recovery, and a 5-min cool- down.<br>- Supervision: fully.<br><br>Control<br>Continue habitual lifestyle.	- Exercise Vs. control	- Systolic blood pressure<br>- Diastolic blood pressure	Short term: 12 weeks
Hinderliter et al, 2014[20]	RCT	Diet+Exercise (N=46)<br>Age: 52.3±10 y<br>BMI: 33.5±4.4 kg/m^2<br>% females: 69<br>Comorbidities: never treated-HTN.<br><br>Diet (N=46).<br>Age: 51.8±10 y<br>BMI: 32.8±3.4 kg/m^2<br>% females: 63<br>Comorbidities: never treated-HTN.	Exercise<br>- Program duration: 16 weeks<br>- Number of sessions: 3/week<br>- Type of training: 10 minutes of warm-up exercises, 30 minutes of biking and/ or walking or jogging at 70%–85% of the initial heart rate reserve, and 5 minutes of cool-down exercises<br>- Supervision: fully.<br><br>Diet<br>DASH diet.	- Diet+exercise vs diet alone	- Systolic blood pressure<br>- Diastolic blood pressure	Short term: 16 weeks
Ho et al, 2012[21]	RCT	Aerobic exercise (N=15)<br>Age: 55 (44-62) y<br>BMI: 32.7 (25-45.6) kg/m^2<br>% females: nr<br>Comorbidities: HTN.<br><br>Resistance exercise (N=16)<br>Age: 52 (43-59) y<br>BMI: 33 (25.8-44.6) kg/m^2<br>% females: nr<br>Comorbidities: HTN.<br><br>Combination exercise (N=17)<br>Age: 53 (43-64) y<br>BMI: 33.3 (23.4-40.2) kg/m^2<br>% females: nr<br>Comorbidities: HTN.	Aerobic exercise<br>- Program duration: 12 weeks<br>- Number of sessions: 5/week<br>- Type of training: 30 minutes of aerobic exercise on a treadmill at 60% heart rate reserve (HRR) ±10 beats/min.<br>- Supervision: partially.<br>Resistance exercise<br>- Program duration: 16 weeks<br>- Number of sessions: 5/week<br>- Type of training: 30-min of resistance exercise (four sets of 8–12 repetitions at 10-RM for leg press, leg curl, leg extension, bench press and rear deltoid row, with each set completed in approximately 30-sec with 1-min rest).<br>- Supervision: partially.<br>Combined exercise<br>- Program duration: 16 weeks<br>- Number of sessions: 5/week<br>- Type of training: combination of 15-min aerobic exercise and 15-min of resistance exercise (two sets of each exercise). 10-RM would be approximately 75% of 1-RM.	- Aerobic exercise vs. control<br>- Resistance exercise vs. control<br>- Combined exercise vs. control	- Systolic blood pressure<br>- Diastolic blood pressure	Short term: 12 weeks

		Control (N=16).<br>Age: 52 (40-66) y<br>BMI: 32.4 (26-48) kg/m^2<br>% females: nr<br>Comorbidities: HTN.	- Supervision: partially.<br><br>Control<br>- take a teaspoon of supplement in a glass of water once/day which contained approximately 2 grams breadcrumbs and 0.1 grams “Equal” artificial sweetener.			
Houghton D et al 2017[22]	RCT	Exercise (N=12)<br>Age: 54±12 y<br>BMI: 33±7 kg/m^2<br>% female: nr<br>Comorbidities: NASH<br><br>Standard care (N=12).<br>Age: 51±16 y<br>BMI: 33±5 kg/m^2<br>% female: nr<br>Comorbidities: NASH	Exercise<br>Program duration: 12 weeks<br>Number of sessions: 3/week<br>Type of training: cycling included a 5-minute warm-up and 3 intervals on a fixed bike for 2 minutes with a 1-minute rest in-between based on the Borg with bike intervals corresponding to a rating of perceived exertion of 16 to 18 (very hard). This was followed by a resistance exercise circuit on a rating of perceived exertion of 14 to 16 (hard). 45-60 minutes/session.<br>Supervision: fully.	- HIIT vs usual care	- Intrahepatic fat.	Short term: 12 weeks
Johnson et al, 2009[23]	RCT	Exercise HIIT (N=12)<br>Age: 49.1 (2.3) y<br>BMI: 32.2 (0.8) kg/m^2<br>Comorbidities: HTN<br><br>Control (N=7)<br>Age: 47.3 (3.6) y<br>BMI: 31.1 (1.1) kg/m^2<br>Comorbidities: HTN<br><br>Overall % female: 34.7	Exercise<br>- Program duration: 4 weeks<br>- Number of sessions: 3/week<br>- Type of training: cycle ergometer sessions (30-45 minutes each) per week, with intensity increased such that subjects exercised at a power output designed to elicit 50% of pretrainingVO2peak for week 1, 60% for week 2, and 70% for weeks 3 and 4. Sessions were undertaken as 15-minute bouts with intervening 5-minute rests.<br>- Supervision: fully.<br><br>Control<br>- 30-minute home-based whole-body stretching routine to perform three times per week.	- Exercise vs. control	- Intrahepatic fat<br>- HOMA-IR	Short term: 4 weeks.
Kadoglou et al, 2010[24]	RCT	Exercise (N=22)<br>Age: 56.91±7.09 y<br>BMI: 31.14±3.58 kg/m^2<br>% female: 63.6<br>Comorbidities: HTN, T2DM.<br><br>Control (N=21)<br>Age: 60.32±9.28 y<br>BMI: 29.96±1.03 kg/m^2<br>% female: 61.9<br>Comorbidities: HTN, T2DM.<br><br>Exercise+rosiglitazone (N=23)<br>Age: 57.083±7.61 y<br>BMI: 28.96± 1.03 kg/m^2<br>% female: 60.8<br>Comorbidities: HTN, T2DM.<br><br>Rosiglitazone alone (N=23)<br>Age: 59.04±7.35 y<br>BMI: 30.04±2.99 kg/m^2<br>% female: 65.2<br>Comorbidities: HTN, T2DM.	Exercise<br>- Program duration: 48 weeks<br>- Number of sessions: 4/week<br>- Type of training: Each session included 10 minutes of warm-up, 30 to 45 minutes of aerobic exercise, and 5 minutes of cooldown. Exercise training at 50% to 80% VO2peak for 45 minutes.<br>- Supervision: partially.<br><br>Rosiglitazone: 8 mg/d<br><br>Control<br>Habitual activities.	- Exercise vs. control.<br>- Exercise+rosiglitazone vs. rosiglitazone alone.	- HOMA-IR<br>- Systolic blood pressure<br>- Diastolic blood pressure	Long term: 48 weeks.

Keating SE et al 2015[25]	RCT	HI:LO (N=12)<br>Age: 44.2 (2.8) y<br>BMI: 36.3 (1.7) kg/m^2<br>% female: 50<br>Comorbidities: none<br><br>LO:HI (N=12)<br>Age: 45.5 (2.3) y<br>BMI: 33.9 (0.9) kg/m^2<br>% female: 58<br>Comorbidities: none<br><br>LO:LO (N=12)<br>Age: 45.6 (3.6) y<br>BMI: 31.3 (0.8) kg/m^2<br>% female: 75<br>Comorbidities: none<br><br>PLA (N=12)<br>Age: 39.1 (2.9) y<br>BMI: 32.2 (1.4) kg/m^2<br>% female: 75<br>Comorbidities: none	Supervision: fully.<br>Program duration: 8 weeks<br>HI:LO High intensity low volume<br>- Number of sessions: 2/week<br>- Type of training: continuous cycling on the ergometer at an intensity of 60–70% of VO2peak+ additional brisk walk at the same intensity at home one day per week. Training progressed from 30 min at 50% VO2peak in week one to 45 min at 70% VO2peak by the third week, totalling 90–135 min per week.<br><br>LO:HI Low to moderate intensity high volume<br>- Number of sessions: 3/week<br>- Type of training: continuous cycling on the ergometer at 50% of VO2peak +brisk walk at the same intensity at home one day per week. Training progressed from 45 min in week one to 60 min by the third week, totalling 180–240 min per week.<br><br>LO:LO Low to moderate intensity low volume<br>- Number of sessions: 2/week<br>- Type of training: continuous cycling on the ergometer at 50% of VO2peak+ brisk walk at the same intensity at home one day per week. Training progressed from 30 min in week one to 45 min by the third week, totalling 90–135 min per week.<br><br>PLA<br>- Number of sessions: 3/week<br>- Type of training: sham exercise stretching, self-massage and fitball program. Participants received one fortnightly supervised session which involved instructions of new exercises and a 5 min cycle at very low intensity (30W) to maintain familiarity with the cycle ergometer.	- HI-LO vs control<br>- LO-HI vs. control<br>- LO-LO vs control	- Intrahepatic fat<br>- Systolic Blood pressure<br>- Diastolic Blood pressure	Short term: 8 weeks
Kim JW et al, 2012[26]	RCT	Overall Mean Age: 54.5± 2.82 y<br><br>Exercise (N=15)<br>BMI: 25 ±1.3 kg/m^2<br>Only females.<br>Comorbidities: none<br><br>Control (N=15).<br>BMI: 25.1 ±1.5 kg/m^2<br>Only females.<br>Comorbidities: none	Exercise<br>- Program duration: 16 weeks<br>- Number of sessions: 3/week<br>- Type of training: 60 min per session on non-consecutive days at a specified intensity based on age-predicted maximal heart rate (HRmax) for 16 weeks. The intensity of exercise was initially set at 55%–65% of the age-predicted HRmax and was gradually increased until 70%–80% of the age-predicted HRmax was reached.<br>- Supervision: fully.<br><br>Control<br>No exercise.	- Exercise vs. control	- Systolic blood pressure<br>- Diastolic blood pressure	Short term: 16 weeks.
Kim YS et al, 2014[27]	RCT	Overall mean age: 25.3±2.3. y<br><br>Exercise (N=29)<br>BMI: 28.5 ±2.4 Kg/m^2<br>Only males.<br>Comorbidities: none.<br><br>Control (N=10).<br>BMI: 28.2±2.4<br>Only males.<br>Comorbidities: none.	Exercise<br>- Program duration: 8 weeks<br>- Number of sessions: 4/week<br>- Type of training: Aerobic exercise was comprised of a treadmill running at 65–75% VO2max to burn approximately 600 Kcal per session. The subjects maintained their habitual recreational physical activities.<br>- Supervision: fully.<br><br>Control<br>No exercise.	- Exercise vs. control	- HOMA-IR	Short duration: 8 weeks.
Kolahdouzi et al, 2018[28]	RCT	Exercise (N=13)<br>Age: 23.0 ±3.8 y	Exercise<br>- Program duration: 8 weeks<br>- Number of sessions: 3/week	- Exercise vs. control	- HOMA-IR	Short term: 8 weeks

		BMI: 30.12 ±2.99 kg/m^2<br>Only males.<br>Comorbidities: HTN.<br><br>Control (N=13).<br>Age: 24.0 ±4.8 y<br>BMI: 31.1±3.2 kg/m^2<br>Only males.<br>Comorbidities: HTN	- Type of training: first four weeks: 65%–75% of 1-RM, two circuits per session, with eight to 12 repetitions in each exercise. Second four weeks: 75%–85% of 1-RM, four circuits per session, with six to eight repetitions in each exercise. Rest between stations: <15 seconds. Rest between circuits: 3 minutes of active recovery. Eight exercises: squat, standing curls, bench press, leg extension, leg flexion, leg press, military press, and lat pull down<br>- Supervision: not reported.<br><br>Control<br>Habitual activities.			
Kozey-Keadle et al, 2014[29]	RCT	Exercise (N=16)<br>Age: 43.9±9.7 y<br>BMI: 35.2±5.3 kg/m^2<br>Comorbidities: HTN, MetS<br><br>Control (N=8)<br>Age: 42.7±10.1 y<br>BMI: 35.3±5.2 kg/m^2<br>Comorbidities: HTN, MetS<br><br>Exercise+reducing sitting time (N=16)<br>Age: 42.4±10.7 y<br>BMI: 35±4.2 kg/m^2<br>Comorbidities: HTN, MetS<br><br>Reducing sitting time (N=14)<br>Age: 44.5±9.5 y<br>BMI: 34.8±4.3 kg/m^2<br>Comorbidities: HTN, MetS<br><br>Overall %females: 67.2	Exercise<br>- Program duration: 12 weeks<br>- Number of sessions: 5/week<br>- Type of training: 40 min (a total of 200 min per week). Treadmill (3 of 5 sessions per week), stationary cycle ergometer or Arctrainer. Week 1: 40%–50% of HRR for 30 min/session; week 2: 50%–60% of HRR for 35 min/session; weeks 3–6: 50%–60% of HRR for 40 min/session; weeks 7–12: 55%–65% of HRR for 40 min/session.<br>- Supervision: fully.<br><br>Reducing sitting time<br>- At the beginning of the intervention period, a trained researcher discussed home, work, and discretionary time strategies to increase participants' non exercise physical activities (e.g., standing during all commercials, taking a 5-min movement break each hour at work) and counselled participants on the benefits of reducing sedentary time.<br><br>Control<br>Habitual activities.	- Exercise vs. control<br>- Exercise+reducing sitting time vs. reducing sitting time	- Systolic blood pressure<br>- Diastolic blood pressure	Short term: 12 weeks
Kucio et al, 2017[30]	RCT	Exercise (N=15)<br>Age: 56.7±5.8 y<br>BMI: 31.8±5.0 kg/m^2<br>Only males.<br>Comorbidities: HTN<br><br>Control (N=11)<br>Age: 57±4.6 y<br>BMI: 31.2±4.2 kg/m^2<br>Only males.<br>Comorbidities: HTN	Exercise<br>- Program duration: 4 weeks<br>- Number of sessions: 5/week<br>- Type of training: Each exercise session consisted of a 10-minute warm- -up. During the first week, the participants performed a march at the speed of 3 km/h for a duration of 30 minutes. During weeks 2–4, the speed of the march was raised to 5 km/h, and the duration of the workout was raised to 40 minutes. During NW training, the patients reached about 40–70% of maximum heart rate.<br>- Supervision: no.<br><br>Control<br>Usual care.	- Exercise vs. control	- Daytime systolic blood pressure<br>- Daytime diastolic blood pressure	Short term: 4 weeks.
Labrunée et al, 2012[31]	RCT	Exercise (N=11)<br>Age: 52.4±8.2 y<br>BMI: 39.3±9.9 kg/m^2<br>% females: 45.5<br>Comorbidities: T2DM, dyslipidaemia.<br><br>Control (N=12)<br>Age: 52.8±8.5 y<br>BMI: 40.1±7.3 kg/m^2	Exercise<br>- Program duration: 12weeks<br>- Number of sessions: 7/week<br>- Type of training: daily cyclergometer session with: an initial 5 min warm-up at 20 W, then 20 min at the cardiac frequency corresponding to the first ventilation threshold measured during the initial test of effort, then active recovery at 20 W during 5 min.<br>- Supervision: partially.<br><br>Control	- Exercise vs. control	- HOMA-IR	Short term: 12 weeks

		% females: 66.6<br>Comorbidities: T2DM, dyslipidaemia.	General advices.			
Larson-Myer et al, 2009[32]	RCT	Diet+Exercise (N=12)<br>Age: 36.±6 y<br>BMI: 27.5±1.6 kg/m^2<br>% females: 58.3<br>Comorbidities: none.<br><br>Diet (N=12)<br>Age: 39±5 y<br>BMI: 27.8±1.4 kg/m^2<br>% females: 50<br>Comorbidities: none.	Diet+Exercise<br>- Program duration: 24 weeks<br>- Number of sessions: 5-7/week<br>- Type of training: increase energy expenditure by 12.5% above baseline requirements by undergoing structured aerobic exercise (i.e., walking, running, or stationary cycling) 5 d/wk. Participants were allowed to select their exercise intensity (as long as their heart rate was within 65%–90% of maximal heart rate).<br>- Supervision: partially.<br>- All diets were based on the American Heart Association Step 1 recommendations.<br><br>Diet<br>All diets were based on the American Heart Association Step 1 recommendations.	- Diet+exercise vs. diet	- Systolic blood pressure<br>- Diastolic blood pressure.	Intermediate term: 24 weeks.
Masuo et al, 2012[33]	RCT	Diet+Exercise (N=30)<br>Age: 38.±5 y<br>BMI: 30.5±1.8 kg/m^2<br>Only males.<br>Comorbidities: HTN.<br><br>Diet (n=30)<br>Age: 38±5 y<br>BMI: 30.2±1.5 kg/m^2<br>Only males.<br>Comorbidities: HTN.	Exercise<br>- Program duration: 24 weeks<br>- Number of sessions: 5-7/week<br>- Type of training: aerobic exercise of >1h daily, for example, walking, jogging or gym exercise.<br>- Supervision: partially.<br>- a low-caloric (1760–1840kcal (22–23 units) per day, 55% of calories from carbohydrate, 30% from protein and 15% from fat) and low-sodium diet (7 g NaCl per day)<br>Diet<br>a low-caloric (1760–1840kcal (22–23 units) per day, 55% of calories from carbohydrate, 30% from protein and 15% from fat) and low-sodium diet (7 g NaCl per day)	- Diet+exercise vs. diet	- HOMA-IR<br>- Systolic blood pressure<br>- Diastolic blood pressure	Intermediate term: 24 weeks.
Meckling et al, 2007[34]	RCT	Control diet (N=8)<br>Age: 47.±12 y<br>BMI: 28.7±2.3 Kg/m^2<br>Only females.<br>Comorbidities: none<br><br>Control diet+exercise (N=11)<br>Age: 41.±10 y<br>BMI: 29.2±3.5 kg/m^2<br>Only females.<br>Comorbidities: none<br><br>High protein diet (N=10)<br>Age: 45.±16 y<br>BMI: 31.2±3.5 kg/m^2<br>Only females.<br>Comorbidities: none<br><br>High protein diet+exercise (N=14)<br>Age: 37±10 y<br>BMI: 30.8±4.7 kg/m^2<br>Only females.<br>Comorbidities: none	Exercise<br>- Program duration: 12 weeks<br>- Number of sessions: 3/week<br>- Type of training: The circuit (36 min) consisted of alternating 60 s resistance and endurance exercise bouts. Subjects began exercising at 65% of their maximum heart rate for the first 3 weeks and gradually increased the intensity to 80% by study's end.<br>- Supervision: partially.<br><br>Control diet<br>- 1 g protein : 3 g of carbohydrate<br><br>High protein diet<br>- 1 g protein : 1 g of carbohydrate	- Exercise+control diet vs. control diet<br>- Exercise +High protein diet vs high protein diet	- Systolic blood pressure<br>- Diastolic blood pressure	Short term: 12 weeks
Mendham et al, 2014[35]	RCT	Exercise (N=11)<br>Age: 39.5±10.6 y<br>BMI: 31.6±3.1 kg/m^2	Exercise<br>- Program duration: 12 weeks<br>- Number of sessions: 2-3/week	- Exercise vs. control	- HOMA-IR<br>- Systolic blood pressure	Short term: 12 weeks.

		Only males.<br>Comorbidities: none.<br><br>Control (N=10)<br>Age: 36.1±16.1 y<br>BMI: 34.5±6.6 kg/m^2<br>Only males.<br>Comorbidities: none.	- Type of training: 45 and 60 min sessions (including 5-10min of dynamic warm-up), with exercise intensity prescribed to maintain 70-85% HRmax. Supervised group-based cardiovascular and resistance exercises (45 min): strength training, core exercises and cardiovascular training of continuous stationary cycling, running and rowing ergometry. An additional session (60 min) comprised of boxing specific circuit training, including multiple stations and passive recovery. Throughout the program work to rest ratio progressed from 1:1 (weeks 1-3), 2:1 (weeks 4-6), 3:1 (weeks 7-9) and 4:1 (weeks 10-12).<br>- Supervision: partially.<br><br>Control<br>General advices.		- Diastolic blood pressure	
Mohr et al, 2014[36]	RCT	HIT (N=21) **<br>Age: 44±2 y<br>Height (cm): 164±1<br>Weight (Kg): 76.5±1.9<br>Only females.<br>Comorbidities: HTN.<br><br>MICT (N=21)**<br>Age: 46±2 y<br>Height (cm): 165±1<br>Weight (Kg): 83.8±4.3<br>Only females.<br>Comorbidities: HTN.<br><br>Control (N=20)**<br>Age: 45±2 y<br>Height (cm): 166±1<br>Weight (Kg): 76.4±2.6<br>Only females.<br>Comorbidities: HTN.	HIT<br>- Program duration: 15 weeks<br>- Number of sessions: 3/week<br>- Type of training: ~15–25min (3–5 min of effective swimming) and consisted of 6–10 30 s all-out free-style swimming (front crawl) intervals interspersed by 2min of passive recovery. First 6wks: 6 intervals, the following 6wks: 8 intervals, and the final 3wks: 10 all-out swimming intervals.<br>- Supervision: fully.<br><br>MICT<br>- Program duration: 15 weeks<br>- Number of sessions: 3/week<br>- Type of training: 1 h and consisted of continuous front crawl swimming where the participants were encouraged to swim as far as possible in every session.<br>- Supervision: fully.<br><br>Control<br>No training or lifestyle changes.	- HIT vs control<br>- MICT vs. control	- Systolic blood pressure<br>- Diastolic blood pressure	Short term: 15 weeks.
Oh S. et al, 2018[37]	RCT	Diet+Exercise (N=10)<br>Age: 37.3 ± 7.3 y<br>BMI: 27.5 ±2.6 kg/m^2<br>% female: 58.3<br>Comorbidities: none<br><br>Diet (N=9)<br>Age: 32.9 ± 7.3 y<br>BMI: 27.6 ± 2.8 kg/m^2<br>% female: 76.9<br>Comorbidities: none<br><br>Exercise (N=8)<br>Age: 35.7 ± 7.9 y<br>BMI: 28.3 ± 4.1 kg/m^2<br>% female: 30<br>Comorbidities: none<br><br>Control (N=8)<br>Age: 40.6 ± 10y	Exercise<br>- Program duration: 8 weeks<br>- Number of sessions: 3/week<br>- Type of training: 1) 5 min of warm-up; 2) 40 min of resistance training; 3) 20 min of aerobic exercise; 4) 5 min of cool-down. Three sets of 4 exercises. Rest between the sets of the exercise was set as 60–90 s. The repetition of resistance training was set as 15 reps a set at 70% 10RM intensity; 12 reps a set at 80% and 90% of 10RM intensity; and 10 reps a set at 100% of 10RM intensity. The aerobic exercise was performed on motorized treadmills for at least 20 min and the intensity of exer- cise was corresponding to approximately 60–85% of age-predicted maximal heart rate.<br>- Supervision: partially.<br><br>Diet<br>- The participants consumed 25% of daily recommend energy intake in 3 days alternately on 'fast days' (400–500 kcal), and consumed ad libitum on the remaining 4 days of the week, known as 'feed days'.<br><br>Control<br>- Habitual lifestyle.	- Diet+exercise vs. diet<br>- Exercise vs. control	- HOMA-IR<br>- Systolic blood pressure<br>- Diastolic blood pressure	Short term: 8 weeks.

		BMI: 26.3 ± 3.0 kg/m^2<br>% female: 60<br>Comorbidities: none				
Plotnikoff et al, 2010[38]	RCT	Exercise (N=27)<br>Age: 55 ±12 y<br>BMI: 35 ±8 kg/m^2<br>% female: 70.4<br>Comorbidities: HTN, T2DM.<br><br>Control group (N=21)<br>Age: 54 ±12 y<br>BMI: 36 ±5 kg/m^2<br>% female: 61.9<br>Comorbidities: HTN, T2DM	Exercise<br>- Program duration: 16 weeks<br>- Number of sessions: 3/week<br>- Type of training: 8 exercises/session, of which, four were core exercises and four were complementary assistance exercises. First week: 2 sets of 10–12 repetitions at 50–60% of 1RM. Week 2, 3 sets of exercises, rest between sets for 90–120 s. Weeks 3–8, exercise intensity was increased to 70–80% 1RM. Week 9: recovery week at 70% of 1RM, only two sets of 8–10. After 1RM retesting at the start of week 10, patients performed three sets of 8–10 repetitions at 70–85% of 1RM with 60–90 s rest throughout weeks 10–15. Week 16: recovery week of 8–10 repetitions at 80% of 1RM.<br>- Supervision: partially.<br><br>Control<br>- No exercise.	- Exercise vs. control	- Systolic blood pressure<br>- Diastolic blood pressure	Short term: 16 weeks.
Pugh et al, 2014[39]	RCT	Exercise (N=13)<br>Age: 48.6 (2.2) y<br>BMI: 37.1 (1.1) kg/m^2<br>% female: 66.6<br>Comorbidities: NAFLD, MetS<br><br>Control group (N=8)<br>Age: 47.5 (3.1) y<br>BMI: 40.0 (2.2) kg/m^2<br>% female: 83.3<br>Comorbidities: NAFLD, MetS	Exercise (N 13)<br>16 weeks moderate exercise.<br>Number of sessions: 3-5/week<br>Type of training: treadmill or cycle ergometer from 30 to 60% of HRR for 30-45 minutes.<br>Supervision: yes.<br><br>Control group (N 8)<br>Lifestyle advice.	- Moderate aerobic exercise vs usual care	- Intrahepatic fat<br>- HOMA-IR<br>- Systolic blood pressure<br>- Diastolic blood pressure	Short term: 16 weeks
Pourranjbar et al, 2018.[40]	RCT	Exercise (N=38)<br>Age (years): 38.15±2.33<br>BMI (Kg/m^2): 30.7±2.33<br>Only females.<br>Comorbidities: none.<br><br>Control (N=42)<br>Age (years): 38.89±1.78<br>BMI (Kg/m^2): 30.01±2.70<br>Only females.<br>Comorbidities: none.	Exercise<br>- Program duration: 8 weeks<br>- Number of sessions: 3/week<br>- Type of training: week (10 minutes of warm-up training, 30 minutes of running with 50–70% of maximum heart rate and 5 minutes of cooling down), with the first two weeks exercising at 50% of maximum heart rate, in the second two weeks at 60%, in the third two weeks at 65%, and in the last two weeks at 70% of the maximum heart rate<br>- Supervision: partially.<br><br>Control<br>- No exercise.	- Exercise vs. control	- HOMA-IR	Short term: 8 weeks
Ryan et al, 2014[41]	RCT	Diet+exercise (N=37)**<br>Age (years): 60±1<br>BMI (Kg/m^2): 32±1<br>Only females.<br>Comorbidities: HTN, dyslipidaemia.<br><br>Diet (N=40)**<br>Age (years): 61±1<br>BMI (Kg/m^2): 33±1<br>Only females.<br>Comorbidities: HTN, dyslipidaemia.	Resistance Exercise<br>- Program duration: 24 weeks<br>- Number of sessions: 3/week<br>- Type of training: using treadmills and elliptical trainers. Each exercise session included a 5- to 10-minute warm-up and a 5- to 10-minute cool- down. Women exercised at approximately 50% to 60% of their heart rate reserve (HRR) and gradually progressed in duration and intensity until they were able to exercise at more than 85% of their HRR for 45 minutes..<br>Supervision: not reported.<br><br>Diet<br>Individuals were instructed to restrict their caloric intake by 300 to 500 kcal/d to achieve weight loss.	- Diet+Exercise vs. Diet	- HOMA-IR<br>- Systolic blood pressure<br>- Diastolic blood pressure	Intermediate term: 24 weeks.

Schroeder et al, 2018 [42]	RCT	Aerobic training (N=17)<br>Age (years): 58±7 y<br>BMI (Kg/m^2): 32.5±5.9 Kg/m^2<br>%females: 59 %.<br>Comorbidities: HTN.<br><br>Resistance training (N=17)<br>Age (years): 57±9 y<br>BMI (Kg/m^2): 33.1±5.9 Kg/m^2<br>%females: 59%.<br>Comorbidities: HTN.<br><br>Combined training (N=18)<br>Age (years): 58±7 y<br>BMI (Kg/m^2): 31.9±5.5 Kg/m^2<br>%females: 61%.<br>Comorbidities: HTN.<br><br>Combined training (N=17)<br>Age (years): 58±6 y<br>BMI (Kg/m^2): 32.4±3.7 Kg/m^2<br>%females: 65%.<br>Comorbidities: HTN.	Aerobic training<br>- Program duration: 8 weeks<br>- Number of sessions: 3/week<br>- Type of training: 60 min treadmill or cycle ergometer. Starting at 40% of their heart rate reserve, participants were progressed to approximately 70% of their heart rate reserve. Participants could choose to exercise at a higher intensity but not to exceed 80% of their heart rate reserve.<br>- Supervision: fully<br><br>Aerobic training<br>- Program duration: 8 weeks<br>- Number of sessions: 3/week<br>- Type of training: 12 exercises; the program started with 2 sets of 18–20 maximal repetitions and progressed to 3 sets of 10–14 maximal repetitions with a rest of 1–2 minutes between sets. Participants achieved exhaustion in each set, indicating the lower the repetition, the higher the intensity rate reserve.<br>- Supervision: fully<br><br>Combination training<br>- Program duration: 8 weeks<br>- Number of sessions: 3/week<br>- Type of training: 30 minutes of aerobic and 30 minutes of resistance exercise per session. Participants followed the same intensity and protocol as the individual groups, but the resistance training was reduced to 2 sets of 8 exercises.<br>- Supervision: fully<br><br>Control<br>Usual care.	- Aerobic training vs. control<br>- Resistance training vs. control<br>- Combination training vs. control.	- Systolic blood pressure<br>- Diastolic blood pressure	Short term: 8 weeks
Shah K. et al, 2009[43]	RCT	Diet + Exercise (N=9)**<br>Age: 68.5 (1.3) y<br>% female: 78 kg/m^2<br>Comorbidities: NAFLD, MetS<br><br>Diet (N=9)**<br>Age: 68.6 (1.1) y<br>% female: 67% Kg/m^2<br>Comorbidities: NAFLD, MetS	Diet + Exercise (N 10)<br>- 24 weeks moderate exercise.<br>- Number of sessions: 3/week<br>- Type of training: 15 min of flexibility exercises, followed by 30 min of aerobic exercise, 30 min of strength training, and 15 min of balance exercises. Total 90 minutes. 70-85% HR peak for aerobic exercise. 65-80% of 1-RM for resistance training.<br>- Supervision: yes (physical therapist).<br><br>Diet (N 9)<br>- Balanced diet to provide an energy deficit of 500–1.000 kcal/day from daily energy requirement	- Diet + Exercise vs Diet	- Intrahepatic fat<br>- HOMA-IR<br>- Systolic blood pressure<br>- Diastolic blood pressure	Intermediate term: 24 weeks
Stensvold et al, 2010[44]	RCT	Aerobic interval training (N=11)<br>Age (years): 49.9±10.1<br>BMI (Kg/m^2): 31.3±4.3<br>%females: nr.<br>Comorbidities: HTN, T2DM, MetS.<br><br>Strength training (N=11)<br>Age (years): 50.9±7.6<br>BMI (Kg/m^2): 32.2±4.2<br>%females: nr.<br>Comorbidities: HTN, T2DM, MetS.<br><br>Combined training (N=10)<br>Age (years): 52.9±10.4	Aerobic interval training<br>- Program duration: 12 weeks<br>- Number of sessions: 3/week<br>- Type of training: 10-min warmup period at 70% of HRpeak; 4 intervals of 4 min at 90–95% of HRpeak; 3-min active recovery period at 70% of HRpeak. 5-min cooldown. Total exercise time of 43 min<br>- Supervision: fully.<br><br>Strength training<br>- Program duration: 12 weeks<br>- Number of sessions: 3/week<br>- Type of training: warmup period where the participants performed 2 sets of 15–20 repetitions at 40–50% of one repetition maximum (1-RM). First week: 60% of each	- Exercise vs. control	- Systolic blood pressure<br>- Diastolic blood pressure	Short term: 12 weeks

		BMI (Kg/m^2): 30.3±3.5<br>%females: nr.<br>Comorbidities: HTN, T2DM, MetS.<br><br>Control (N=11)<br>Age (years): 47.3±10.2<br>BMI (Kg/m^2): 31.9±4.1<br>%females: nr.<br>Comorbidities: HTN, T2DM, MetS.	individual's 1-RM. After three sets at 80% of 1-RM (corresponding to a maximum of 8–12 repetitions). The total exercise time was 40 -50 min.<br>- Supervision: fully.<br><br>Combined training<br>- Aerobic interval training twice a week and strength training once a week		
Straznicky et al, 2012[45]	RCT	Diet+exercise (N=19)<br>Age: 55 ±6 y<br>BMI: 32.9 ± 4.9 Kg/m^2<br>% female: 43<br>Comorbidities: MetS<br><br>Diet (N=20)<br>Age: 54 ± 4 y<br>BMI: 32.4 ± 3.9 Kg/m^2<br>% female: 36<br>Comorbidities: MetS	Diet+exercise<br>- Program duration: 12 weeks<br>- Number of sessions: 3/week<br>- Type of training: 40-min bicycle riding on alternate days at a moderate intensity of 65% of predetermined maximum heart rate. Workload was increased as necessary to maintain target heart rate<br>- Supervision: fully<br><br>Diet<br>DASH: modified dietary approaches to stop hypertension at a moderate energy deficit of 30%.	- Diet+Exercise vs.diet	- HOMA-IR<br>- Systolic blood pressure<br>- Diastolic blood pressure	Short term: 12 weeks
Sullivan et al, 2012[46]	RCT	Exercise (N=12)<br>Age: 48.6 (2.2) y<br>BMI: 37.1 (1.1) Kg/m^2<br>% female: 66.6<br>Comorbidities: NAFLD<br><br>Control group (N=6)<br>Age: 48 (2) y<br>BMI: 40.0 (2.2) Kg/m^2<br>% female: 83.3<br>Comorbidities: NAFLD	Exercise<br>- 16 weeks moderate exercise.<br>- Number of sessions: 5/week<br>- Type of training: brisk walking 45% to 55% of their maximum VO2 peak for 30-60 minutes.<br>- Supervision: partially.<br><br>Control group<br>Current activities of daily living.	- Exercise vs control	- Intrahepatic fat.	Shirt term: 16 weeks
Swift et al, 2012 [47]	RCT	Exercise 8 KKW (N=82)<br>Age: 56.6±6.3 y<br>BMI: 32.2 ± 4.0 Kg/m^2<br>Only females<br>Comorbidities: HTN, MetS.<br><br>Exercise 12 KKW (N=93)<br>Age: 56.8 ± 6.4 y<br>BMI: 31.1 ± 3.6 Kg/m^2<br>Only females<br>Comorbidities: HTN, MetS.<br><br>Control (N=89)<br>Age: 57.0 ± 5.8 y<br>BMI: 32.0 ± 4.0 Kg/m^2<br>Only females<br>Comorbidities: HTN, MetS.	Exercise<br>- Program duration: 24 weeks<br>- Number of sessions: 3/week<br>- Type of training: During the first week, each group expended 4 KKW. Those assigned to that treatment arm (4-KKW group) continued to expend 4 KKW for 6 months. All the other groups increased their energy expenditure by 1 KKW until they reached the energy expenditure required for their group<br>- Supervision: fully<br><br>Diet<br>- Usual lifestyle.	- Exercise vs. control	- Systolic blood pressure<br>- Diastolic blood pressure	Intermediate term: 24 weeks.
Taghian et al, 2014[48]	RCT	Exercise (N=10)<br>BMI: 32.71 ±3.73 Kg/m^2<br>Only females<br>Comorbidities: none	Exercise<br>- Program duration: 12 weeks<br>- Number of sessions: 3/week<br>- Type of training: warm-up: 10 minutes (walking, stretching, and jog- ging). Principal aerobic activity: maximum heart rate of 60-65% progressing during the first week to 25-	- Exercise vs. control	- HOMA-IR	Short term: 12 weeks

		Control (N=10)<br>BMI: 33.02 ±0.98 Kg/m^2<br>Only females<br>Comorbidities: none<br><br>Overall mean age: 37.0 ± 9.89	30 minutes. Third week: 75-80%, 35-40 minutes. Seventh week: 80-85%, 45-50 minutes. At the end of each session slow cool- down time with stretching exercises which would last for 10 minutes.<br>Control<br>- No exercise.			
Tjønna et al, 2018[49]	RCT	Continuous moderate exercise (N=8)<br>Age: 52 ± 10.6 y<br>BMI: 29.4± 4.9 Kg/m^2<br>% female: 50<br>Comorbidities: HTN, MetS, T2DM.<br><br>Aerobic interval training (N=11)<br>Age: 55.3 ± 13.2 y<br>BMI: 29.8± 5.5 Kg/m^2<br>% female: 63.6<br>Comorbidities: HTN, MetS, T2DM.<br><br>Control (N=9)<br>Age: 49.6 ± 9.0 y<br>BMI: 32.1± 3.3 Kg/m^2<br>% female: 44.4<br>Comorbidities: HTN, MetS, T2DM.	- Both exercise groups performed endurance training as walking/ running "uphill" on a treadmill 3 times a week for 16 weeks.<br><br>Continuous moderate exercise<br>- Program duration: 16 weeks<br>- Number of sessions: 3/week<br>- Type of training: 47 minutes at 70% of HRmax at each exercise session<br>- Supervision: nr.<br><br>Aerobic interval training<br>- Program duration: 16 weeks<br>- Number of sessions: 3/week<br>- Type of training: warm-up for 10 minutes at 70% of maximal heart frequency (Hfmax) before performing four 4-minute intervals at 90% of Hfmax with a 3-minute active recovery at 70% of Hfmax between intervals and a 5-minute cool-down period, giving a total exercise time of 40 minutes<br>- Supervision: nr.<br>Control<br>- General advices.	- Exercise vs. control	- Systolic blood pressure<br>- Diastolic blood pressure	Short term: 16 weeks
Waib et al, 2011[50]	RCT	Exercise (N=55)<br>Age: 49 (47-52) y<br>BMI: 30.0 (28.8-31.2) Kg/m^2<br>% females: 54.5<br>Comorbidities: HTN<br><br>Control (N=24)<br>Age: 53 (50-56) y<br>BMI: 29.6 (27.8-31.5)<br>%females:75<br>Comorbidities: HTN	Exercise<br>- Program duration: 12 weeks<br>- Number of sessions: 5/week<br>- Type of training: 5-minute stretching warm-up, 50-minute jogging on an electronic treadmill, 5-minute cool-down) at an exercise intensity corresponding to 50% to 70% VO2max.<br>- Supervision: fully.<br>Control<br>General advices.	- Exercise vs. control	- HOMA-IR<br>- Systolic blood pressure<br>- Diastolic blood pressure	Short term: 12 weeks
Winding et al, 2017[51]	RCT	Endurance training (N=12)<br>Age: 58±8 y<br>BMI: 27.4±3.1 Kg/m^2<br>% female: 41.6<br>Comorbidities: T2DM.<br><br>HIIT (N=13)<br>Age: 54± 6 y<br>BMI: 28.1±3.5 Kg/m^2<br>% female: 49.3<br>Comorbidities: T2DM.<br><br>Control (N=7)<br>Age: 57±7 y<br>BMI: 28.0±3.5 Kg/m^2<br>% female: 28.5<br>Comorbidities: T2DM.	Endurance training<br>- Program duration: 11 weeks<br>- Number of sessions: 3/week<br>- Type of training: bicycle intervention (40 min/session); brief 5 minute standardized warm-up (40% of Wpeak), 40 minutes of cycling at 50% of Wpeak.<br>- Supervision: not reported.<br><br>HIIT<br>- Program duration: 16 weeks<br>- Number of sessions: 3/week<br>- Type of training: bicycle intervention (20 min/session); brief 5 minute standardized warm-up (40% of Wpeak), 20 minutes of cycling consisting of cycles of 1 min at 95% Wpeak and 1 minute of active recovery (20% Wpeak).<br>- Supervision: not reported.<br><br>Control<br>No exercise.	- Exercise vs. control	- HOMA-IR<br>- Systolic blood pressure<br>- Diastolic blood pressure	Short term: 11 weeks

Winn et al, 2018[52]	RCT	HIIT (N=8)<br>Age: 41 ± 14 y<br>BMI: 32.4 ± 3.9 Kg/m²<br>% female: 36<br>Comorbidities: T2DM, NAFLD<br><br>MICT (N=8)<br>Age: 46 ± 9 y<br>BMI: 40.3 ± 5.2 Kg/m²<br>% female: 36<br>Comorbidities: T2DM, NAFLD<br><br>Control (N=5)<br>Age, 51.4 ± 5.7 y<br>BMI, 30.3 ± 1.7 kg/m²<br>Comorbidities: T2DM, NAFLD	HIIT<br>- Program duration: 4 weeks<br>- Number of sessions: 4/WEEK<br>- Type of training: 4 min intervals, 80% VO2peak; separated by 3 min active recovery, 50% VO2peak<br>- Supervision: fully<br><br>MICT<br>- Program duration: 12 weeks<br>- Number of sessions: 4/WEEK<br>- Type of training: motorized treadmill 55% VO2-peak.<br>- Supervision: fully<br><br>Control<br>- No exercise	- Exercise vs. control	- Intrahepatic fat.	Short term: 4 weeks
Zelber-Sagi et al, 2014[53]	RCT	Exercise (N=33)<br>Age: 46.32±10.32 y<br>BMI: 30.75±4.52 Kg/m²<br>% female: 51.5<br>Comorbidities: HTN, NAFLD<br><br>Control (N=31)<br>Age: 46.64±11.4 y<br>BMI: 31.3±4.14 Kg/m²<br>% female: 41.9<br>Comorbidities: HTN, NAFLD	Exercise<br>- Program duration: 12 weeks<br>- Number of sessions: 3/week<br>- Type of training: Exercises included: leg press, leg extension, leg curl, seated chest press, seated rowing, latissimus pull down, biceps curl and shoulder press with 8-12 repetitions, 3 sets for each exercise with 1-2 min rest between sets, for a total duration of about 40 min.<br>- Supervision: fully<br><br>Control<br>- Stretching.	- Exercise vs. control	- HOMA-IR	Short term: 12 weeks
Zhang et al, 2016[54]	RCT	Vigorous exercise (N=66)<br>Age: 53.2 (7.1) y<br>BMI: 27.9 (2.7) Kg/m²<br>% female: 71.2<br>Comorbidities: HTN, DM2, dyslipidaemia<br><br>Moderate exercise (N=69)<br>Age: 54.4 (7.4) y<br>BMI: 28.1 (3.3) Kg/m²<br>% female: 69.9<br>Comorbidities: HTN, T2DM, dyslipidaemia<br><br>Control group (N=73)<br>Age: 54.0 (6.8) y<br>BMI: 28.0 (2.7) Kg/m²<br>% female: 62.2<br>Comorbidities: HTN, T2DM, dyslipidaemia	Vigorous exercise<br>6 months vigorous +6 months moderate exercise.<br>Number of sessions: 5/week<br>Type of training: jogged on a treadmill and gradually increased exercise intensity so that their heart rate was 65%to 80% of their maximum predicted heart rate (equivalent to 8.0-10.0 metabolic equivalents). They were instructed to exercise at this intensity for 30 minutes.<br>Supervision: nr.<br><br>Moderate exercise<br>12 months moderate exercise.<br>Number of sessions: 5/week<br>Type of training: briskly walk at approximately 120 steps per minute so that their heart rate was 45% to 55%<br>of their maximum predicted heart rate (equivalent to 3.0-6.0 metabolic equivalents) for 30 minutes.<br>Supervision: nr.<br><br>Control group<br>Habitual activities	- Exercise vs. control	- Intrahepatic fat	Intermediate term: 24 weeks

Abbreviations: 1-RM: one repetition maximum; BMI: body mass index; HIIT: high intensity interval training; HR: heart rate; HRR: heart rate reserve; HTN: arterial hypertension; KKW: Kcal/week; MetS: metabolic syndrome; MICT: moderate intensity continuous training; NAFLD: non alcoholic fatty liver disease; NASH: non alcoholic steatohepatitis; nr: not reported. RM: repetition maximum; T2DM: type 2 diabetes; y: years. Unless otherwise specified, values are presented as mean±SD. * median (IQR). ** mean±SEM. *** mean (95%CI).

Table 4. Findings of original studies.

Reference	Findings	Study author's conclusion	Overview authors' assessment of conclusions
Abdelaal et al, 2014[1]	Aerobic pre vs post<br>Systolic blood pressure (mmHg): 145.3±2.2 vs 138.3±1.17<br>Diastolic blood pressure (mmHg): 93.7±1.34 vs 88.05±1.05<br><br>Resistance pre vs post<br>Systolic blood pressure (mmHg): 145.5±1.91 vs 141.0±5.97<br>Diastolic blood pressure (mmHg): 94.0±0.86 vs 91.2±1.24<br><br>Control pre vs post<br>Systolic blood pressure (mmHg): 145.0±2.94 vs 145.26±2.83<br>Diastolic blood pressure (mmHg): 94.0±1.63 vs 94.21±1.44	Within-groups comparison revealed that there were significant decrease in systolic blood pressure mean values between evaluation 1 (baseline) and evaluation 2 (end of treatment) in exercise groups, while there was non-significant increase in control group. Within-groups comparison revealed that there were significant decrease in DBP mean values between the 1 (baseline) and evaluation 2 (end of treatment), while there was non-significant increase in DBP of the control group.	Agree that results demonstrate small, but significant benefit from intervention.
Abdelbasset et al, 2019[2]	HIIT pre vs post<br>Intrahepatic triglycerides (%): 12.4±4.5 vs 10.1±1.3<br>HOMA-IR: 4.9 ±1.7 vs. 4.1±0.6<br><br>Control pre vs post<br>Intrahepatic triglycerides (%): 11.2±5.1 vs 11.1±5.2<br>HOMA-IR: 4.8 ±1.5 vs. 4.98±1.8	According to the present study findings, 8-week HII exercise in form of cycling exercise at 80% to 85% of the VO2max with interval at 50% of the VO2max for 40 minutes 3 times per week showed a significant reduction in IHTG.	Appropriate conclusions based on available data.
Abdelbasset et al, 2020[3]	HIIT pre vs post<br>Intrahepatic triglycerides (%): 12.9±4.2 vs 10.5±1.5<br>HOMA-IR: 4.7 ±1.4 vs. 3.9±0.5<br><br>Control (16) pre vs post<br>Intrahepatic triglycerides (%): 11.2±5.1 vs 11.1±5.2<br>HOMA-IR: 4.8 ±1.7 vs. 4.98±1.8	The results of the present trial emphasized that moderate-intensity continuous exercise three times per week for eight weeks (cycling exercise at 60–70% of max HR for 40–50 minutes) exhibited a definite decrease of hepatic triglycerides.<br>The post-intervention outcomes showed significant differences in favor of the HII group.	Non-significant difference between mean changes in intervention and control group but significant difference in post intervention outcome.
Andersen et al, 2014[4]	Exercise pre vs. post **<br>HOMA-IR : 3.3±0.6 vs. 2.4±0.4<br><br>Control pre vs. post **<br>HOMA-IR : 2.9±0.5 vs. 3.7±0.6	HOMA-IR did not change during intervention program.	Appropriate conclusions based on available data.
Balducci et al, 2012[5]	Exercise pre vs. post **<br>Systolic blood pressure (mmHg): 143±20-136±14 p=0.001<br>Diastolic blood pressure (mmHg): 82±11-79±8 p=0.012<br><br>Control pre vs. post**<br>Systolic blood pressure (mmHg): 145±19-142±14 p=0.27<br>Diastolic blood pressure (mmHg): 86±11-84±7 p=0.27	Systolic and diastolic BP were significantly reduced in the exercise group, whereas only total cholesterol decreased significantly in the control group.	Small but significant benefit from intervention in improving long-term glycaemic control and blood pressure.
Bouchonville et al, 2014[6]	HOMA-IR<br>Exercise:-0.7±1.8<br>Control: -0.3±2.9<br>Diet+exercise:-1.7±1.9<br>Diet: -1.4 ±1.8<br>Systolic blood pressure (mmHg)<br>Exercise:-1.2±15.5<br>Control: -5.9±23	Diet-induced weight loss alone, but not exercise training alone, improved insulin sensitivity. Importantly, the combination of these two interventions resulted in an even greater improvement in insulin sensitivity at 12 months, a novel finding in this population, suggesting a distinct complementary effect of exercise training added to weight loss. In conclusion, lifestyle interventions associated with weight loss result in clinically important improvements in insulin sensitivity and multiple other cardio-metabolic risk factors in obese older adults.	Appropriate conclusions based on available data.

	Diet+exercise:-15.9±18.9<br>Diet: -13.1±15.1<br><br>Diastolic blood pressure (mmHg)<br>Exercise:-2.1±6.1<br>Control: -1.1±11.2<br>Diet+exercise:-4.9±9.5<br>Diet: -6.7±10.3		
Cao et al, 2019 [7]	Exercise Pre vs. post<br>Systolic blood pressure (mmHg): 135±16 vs. 131±16<br>Diastolic blood pressure (mmHg): 83±12 vs. 81±10<br><br>Control Pre vs. post<br>Systolic blood pressure (mmHg): 136±14 vs. 136±14<br>Diastolic blood pressure (mmHg): 80±11 vs. 84±5	Following 12 weeks of FATmax training, beneficial effects in diastolic BP were attained in the Exercise group, compared to the Control group he 12-week FATmax exercise training.	Appropriate conclusions based on available data.
Croymans et al, 2014[8]	Exercise***<br>Systolic blood pressure (mmHg) -6.5 (-13.5-0.0)<br>Diastolic blood pressure (mmHg) -5.0 (-7.5- -1.5)<br><br>Controls***<br>Systolic blood pressure (mmHg) -1.0 (-10.0-7.5)<br>Diastolic blood pressure (mmHg) -5.0 (-16.0-5.0)	RT reduces both central and brachial blood pressures without weight loss and independently of effects on arterial stiffness in overweight/obese young men.	Appropriate conclusions based on available data.
Cuthbertson D et al 2016[9]	Exercise group change ***<br>Intrahepatic fat -9.3 (-13.1,-5.3)<br>HOMA-IR: -0.43 (-0.81,-0.05)<br><br>Control group ***<br>IHCL -2.5 (-6.2, 1.2)<br>HOMA-IR: -0.03 (-0.3, 0.4)	We have demonstrated in a randomized controlled study that 16 weeks of supervised moderate-intensity aerobic exercise in NAFLD reduces liver fat.	Agree that results demonstrate significant benefit from intervention
Fenkci et al, 2006[10]	Aerobic Exercise Pre vs. post<br>Systolic blood pressure (mmHg): 125±17.7 vs. 117.4±14.2<br>Diastolic blood pressure (mmHg): 82.6±9.7 vs. 77.6±10.3<br>HOMA-IR: 3.06±1.3 vs. 2.18±1.1<br><br>Resistance Exercise Pre vs. post<br>Systolic blood pressure (mmHg): 123.5±14.6 vs. 114.7±10.7<br>Diastolic blood pressure (mmHg): 81.8±8.1 vs. 74.4±9<br>HOMA-IR: 2.94±1.05 vs. 3.09±1.50<br><br>Control Pre vs. post<br>Systolic blood pressure (mmHg): 127.3±14.3 vs. 123.7±17.2<br>Diastolic blood pressure (mmHg): 84.0±9.7 vs. 80.7±8.8<br>HOMA-IR: 3.4±1.9 vs. 3.5±1.9	Intragroup comparisons showed that after 12 week of exercise, significant decreases in systolic blood pressure and diastolic blood pressure in all study groups. However, significantly reduced HOMA-IR was observed only in group 1 (Aerobic exercise).	Appropriate conclusions on available data.
Figueroa et al, 2012[11]	Pre vs. post **<br>Exercise<br>Systolic blood pressure 117±3 vs. 112±2<br>Diastolic blood pressure 64±2 vs. 62±2<br><br>Control **<br>Systolic blood pressure 116±2 vs. 118±2<br>Diastolic blood pressure 64±2 vs. 66±2	There was significant treatment-by-time interactions for brachial systolic blood pressure ($P<0.01$). We found that whole body vibration training decreased brachial SBP (-5.3 mmHg).	Significant benefit from intervention despite small sample size.

Figueroa et al, 2015[12]	Pre vs. post **<br>Exercise- high ankle blood pressure<br>Systolic blood pressure 143±4 vs. 132±4<br><br>Exercise-normal ankle blood pressure**<br>Systolic blood pressure 134±2 vs. 122±4<br><br>Control **<br>Systolic blood pressure 139±3 vs. 141±3	Mean brachial systolic blood pressure was similarly decreased in the whole body vibration-high ankle-pressure and whole body vibration -normal ankle-pressure groups, respectively, compared with the control group.	Agree that results demonstrate significant benefit from intervention
Fritz et al, 2012[13]	Normal glucose tolerance<br>Exercise<br>Systolic blood pressure: 2±11.9<br>Diastolic blood pressure: 0.9±7.8<br>HOMA-IR: -0.1±0.4<br>Control<br>Systolic blood pressure: -2.1±11.9<br>Diastolic blood pressure: -0.3±8.0<br>HOMA-IR: 0.02±1.8<br><br>Impaired glucose tolerance<br>Exercise<br>Systolic blood pressure: -13.1±10.7<br>Diastolic blood pressure: -3.8±9.1<br>HOMA-IR: -0.1±0.4<br>Control<br>Systolic blood pressure: 1.6±11.1<br>Diastolic blood pressure: 0.5±7.3<br>HOMA-IR: 0.04±1.4<br><br>Type 2 Diabetes<br>Exercise<br>Systolic blood pressure: 3.9±13.9<br>Diastolic blood pressure: -3.3±13.5<br>HOMA-IR: -0.04±0.4<br>Control<br>Systolic blood pressure: -3.8±15.2<br>Diastolic blood pressure: -.1.7±8.7<br>HOMA-IR: -0.6±2.7	Blood pressure and HOMA IR were unaltered between the normal glucose tolerance, impaired glucose tolerance and T2DM exercise and control groups.	Appropriate conclusions based on available data.
Garcia-Unciti et al, 2012[14]	Exercise +Diet<br>HOMA-IR: -1.1±1.3<br><br>Diet<br>HOMA-IR: -1.4±1.4	No significant differences were observed in these anthropometric variables in either intervention group.	Appropriate conclusions based on available data.
Goodpaster et al, 2010[15]	Exercise pre vs. post ***<br>HOMA-IR : 4.03 (3.47-4.60) vs. 2.70 (2.30-3.11)<br>Systolic blood pressure (mmHg): 135.43 (132.08-138.78) Vs. 132.04 (128.14-135.94)<br>Diastolic blood pressure (mmHg): 78.01 (75.97-80.05) Vs. 75.68 (73.29-78.08).<br><br>Control pre vs. post ***	Blood pressure was significantly and similarly reduced in both intervention groups. Insulin resistance improved significantly and similarly in both intervention groups.	Appropriate conclusions based on available data.

	HOMA-IR : 3.68 (3.07-4.28) Vs. 2.65 (2.23-3.07)<br>Systolic blood pressure (mmHg): 134.43 (130.97-137.88) Vs. 132.57 (128.59-136.54)<br>Diastolic blood pressure (mmHg): 77.0 (74.89-79.1) Vs. 75.46 (73.03-77.89).		
Gorostegi-Anduaga et al, 2018[16]	Diet+High volume MICT pre vs. post.<br>Systolic blood pressure (mmHg): 132.7±12.7 vs. 125.4±8.9<br>Diastolic blood pressure (mmHg): 75.4±8.0 vs. 72±6.7<br><br>Diet+High volume HIIT pre vs. post.<br>Systolic blood pressure (mmHg): 131.7±10.4vs. 127.1±9.7<br>Diastolic blood pressure (mmHg): 79.0±6.9 vs. 74.1±6.2<br><br>Diet+Low volume HIIT pre vs. post.<br>Systolic blood pressure (mmHg): 135.6±13.2 vs. 127.1±10.5<br>Diastolic blood pressure (mmHg): 78.2±8.2 vs. 73.9±7.4<br><br>Diet pre vs. post (mean±SD).<br>Systolic blood pressure (mmHg): 140.0±13.2 vs. 133.0±15.3<br>Diastolic blood pressure (mmHg): 79.9±7.2 vs. 75.1±9.1	Following the 16-week intervention, resting systolic blood pressure, diastolic blood pressure and mean blood pressure decreased (P<0.05). Hence, the hypocaloric DASH diet along with both supervised aerobic exercise and no supervised physical activity recommendations could offer an optimal non- pharmacological tool in the management of hypertension.	Appropriate conclusions based on available data.
Hallsworth K et al 2012[17]	Resistance Exercise Pre vs. post;<br>Intrahepatic lipid (%) 14.0 (9.1) vs. 12.2 (9.0);<br>HOMA-IR: 5.9 (5.9) vs. 4.6 (4.6)<br><br>Control Pre vs. post<br>Intrahepatic lipid (%) 11.2 (8.4) vs 11.5 (7.4);<br>HOMA-IR: 4.7 (2.1) vs. 5.1 (2.5)	An 8-week resistance exercise program brought about an approximately 13% reduction in liver fat without changes in body weight.<br>We observed a pure exercise effect on IHL, which did not involve any change in visceral fat in the patients.	Agree that results demonstrate benefit between intervention and control despite non-significant.
Hallsworth K et al 2015[18]	Resistance Exercise Pre vs. post<br>Intrahepatic lipid (%)-10.6 (4.9) vs. 7.8 (2.4);<br>HOMA-IR: 2.4 (0.8) vs. 2.1 (0.8)<br><br>Control Pre vs. post<br>Intrahepatic lipid (%)10.3 (4.4) vs 10.4 (3.9)<br>HOMA-IR: 1.9 (0.9) vs. 1.9 (0.6)	HIIT performed three times per week for 12 weeks led to a 27% reduction in IHL in people with clinically defined NAFLD. It should be noted that the changes in IHL with HIIT are modest when compared with more substantive weight loss programmes, which can deliver a ~80% reduction in IHL with 8% loss of body weight.	Significant effect within group.<br>Small and non significant effect when compared to controls.
Heydary et al, 2013[19]	Exercise **<br>Systolic blood pressure (mmHg): -4.8±4.7<br>Diastolic blood pressure (mmHg): -5.9±3.3<br><br>Control **<br>Systolic blood pressure (mmHg): 1.6±2.5<br>Diastolic blood pressure (mmHg): 1.6±2.3	Systolic and diastolic blood pressure significantly decreased in the exercise compared to the control group.	Significant benefit from intervention despite small sample size.
Hinderliter et al, 2014[20]	Diet+Exercise ***<br>Systolic blood pressure (mmHg): -16.1 (-19.2;-13.0)<br>Diastolic blood pressure (mmHg): -9.9 (-11.6;-8.1)<br><br>Diet ***.<br>Systolic blood pressure (mmHg): -11.2 (-14.3;-8.1)<br>Diastolic blood pressure (mmHg): -7.5 (-9.3;-5.8)	Blood pressure was reduced in the diet-weight management group by 16.1 (95% confidence interval (CI) = 13.0–19.2)/9.9 (95% CI = 8.1–11.6) mm Hg, compared with 11.2 (95% CI = 8.1– 14.3)/7.5 (95% CI = 5.8–9.3) mm Hg in DASH and the contrast between DASH-weight management and DASH was statistically significant for both systolic BP (P = 0.01) and diastolic BP (P = 0.03).	Appropriate conclusions based on available data.
Ho et al, 2012[21]	Aerobic exercise pre vs. post**.<br>Systolic blood pressure (mmHg): 119.93±4.064 vs. 120.53±4.076	SBP decreased significantly in the control group by 3.3% (P=.038) and in the combination group by 4.2% (P=.034) at week 12 compared with baseline (Table II). DBP decreased significantly in the control group by 3.3% (P=.039) at week 12 compared with baseline (Table II). Although DBP	Appropriate conclusions based on available data.

	Diastolic blood pressure (mmHg): 67.4±2.054 vs. 67.6±2.244<br><br>Resistance exercise pre vs. post **<br>Systolic blood pressure (mmHg): 125.94±4.757 vs. 124.25±4.704<br>Diastolic blood pressure (mmHg): 70.94±2.274 vs. 69.94±2.578<br><br>Combined exercise pre vs. post**.<br>Systolic blood pressure (mmHg): 117.71±3.284 vs. 112.76±3.623<br>Diastolic blood pressure (mmHg): 66.41±1.544 vs. 63.53±1.978<br><br>Aerobic exercise pre vs. post**.<br>Systolic blood pressure (mmHg): 120.06±1.707 vs. 116.06±2.584<br>Diastolic blood pressure (mmHg): 65.38±1.893 vs. 63.19±1.701	decreased at the end of the study from baseline by 4.3% in the combination exercise group, this difference was not statistically significant (P=.055).	
Houghton et al, 2017[22]	Exercise Pre vs. post **<br>Hepatic triglyceride content: 12 (9) vs. 10 (6)<br>HOMA-IR: 2.3 (1.4) vs. 1.9 (0.8)<br><br>Control Pre vs. Post**<br>Hepatic triglyceride content: 10 (5) vs. 11 (5)<br>HOMA-IR: 1.6 (1.1) vs. 1.7 (1.0)	12 weeks of exercise resulted in: (1) a 16% reduction in liver fat in adults with biopsy-proven NASH, independent of weight loss.	Appropriate conclusions based on available data
Johnson et al, 2009[23]	Exercise pre vs. post **<br>HOMA-IR: 4.59±0.69 vs. 4.40±0.76<br>Intrahepatic fat (%): 8.55±2.49 vs. 6.79±1.9<br><br>Control pre vs. post **<br>HOMA-IR): 4.75±1.09 vs. 4.81±0.93<br>Intrahepatic fat (%): 9.18±3.08 vs. 9.44±3.89	Mean intrahepatic fat was significantly reduced in the Exercise group versus the Placebo group (P = 0.042) Ten of 12 individuals in the Exercise group demonstrated a lowering of intrahepatic fat, compared with only one in the Placebo group. Mean reduction in HTGC in the Exercise group was 21%.	Appropriate conclusions based on available data
Kadoglou et al, 2010[24]	Exercise mean.<br>HOMA-IR: -0.85±0.85<br>Systolic blood pressure (mmHg): -6.29±4.25<br>Diastolic blood pressure (mmHg): -2.86±4.29<br><br>Control mean<br>HOMA-IR: 2.12±2.16<br>Systolic blood pressure (mmHg): 0.79±2.54<br>Diastolic blood pressure (mmHg): 0.89±8.48<br><br>Rosiglitazone+exercise.<br>HOMA-IR: -3.25±2.81<br>Systolic blood pressure (mmHg): -13.08±6.28<br>Diastolic blood pressure (mmHg): -7.31±3.35<br><br>Rosiglitazone alone<br>HOMA-IR: -1.87±2.44<br>Systolic blood pressure (mmHg): -6.38±3.22<br>Diastolic blood pressure (mmHg): -3.45±4.45	Exercise training exerted beneficial effects on insulin resistance (HOMA- IR), and systolic BP compared with controls (P < .05). The addition of exercise training to rosiglitazone further decreased HOMA-IR as compared with CO (P b .001) and EX (P = .018) groups.	Appropriate conclusions based on available data

Keating et al, 2015[25]	Intrahepatic fat **<br>HI:LO -2.38 ± 0.73<br>LO:HI -2.62 ± 1.00<br>LO:LO -0.84± 0.47<br>PLA 1.10 ± 0.62<br><br>Systolic blood pressure **<br>HI:LO -4.83 ± 2.49 mmHg<br>LO:HI -5.60 ± 2.04 mmHg<br>LO:LO 5.76± 1.76 mmHg<br>PLA 1.33 ± 1.99 mmHg.<br><br>Diastolic blood pressure**<br>HI:LO -3.83 ± 1.80 mmHg,<br>LO:HI -8.05 ± 2.03 mmHg,<br>LO:LO -1.67± 2.42 mmHg<br>PLA -0.17 ± 1.60 mmHg	Significant reduction in liver fat in all aerobic exercise intervention groups, with the effect size when compared with PLA being large (ES = 1.42, 1.23, and 0.96 for the HI:LO, LO:HI, and LO:LO groups, respectively). These benefits were observed in the absence of meaningful weight loss. We did not detect any difference between the exercise intervention doses.	Appropriate conclusions based on available data
Kim JW et al, 2012[26]	Exercise pre vs. post<br>Systolic blood pressure: 133.23±5.05 vs. 124.62±4.65<br>Diastolic blood pressure: 86.08±6.6 VS. 78.54±3.86<br>HOMA-IR: 2.14±0.18 vs.2.07±0.14<br><br>Control pre vs. post<br>Systolic blood pressure: 131.62±3.71 vs. 133.77±3.17<br>Diastolic blood pressure: 86.92±2.9 vs. 88.69±3.22<br>HOMA-IR: 2.16±0.18 vs. 2.25±1.10	Postmenopausal women who engage in regular and continuous physical activity improve blood pressure and HOMA-IR.	Appropriate conclusions based on available data.
Kim YS et al, 2014[27]	Exercise pre vs. post<br>HOMA-IR: 1.1±1.02 vs.0.57±0.43<br><br>Control pre vs. post<br>HOMA-IR: 0.99±0.84 vs. 1.26±0.86	The exercise induced a significant improvement in HOMA-IR.	Appropriate conclusions based on available data.
Kolahdouzi et al, 2018[28]	Exercise pre vs. post<br>HOMA-IR: 1.69±0.23 vs. 1.21±0.22<br><br>Control pre vs. post<br>HOMA-IR: 1.53±0.35 vs. 1.58±0.27	HOMA-IR (P<.001) significantly decreased in response to circuit resistance training. Also, there was no significant difference in HOMA-IR (P=.405) in the post-test compared to the pre-test in the control group.	Significant benefit from intervention despite small sample size.
Kozey-Keadle et al, 2014[29]	Exercise pre vs. post<br>Systolic blood pressure: 124.9±11 vs. 117.9±8.4<br>Diastolic blood pressure: 78.2±8.4vs. 76.9±8.5<br><br>Control pre vs. post<br>Systolic blood pressure: 133.8±7.2 vs. 127.9±9.5<br>Diastolic blood pressure: 80.1±10 vs. 78.3±6.3<br><br>Exercise+reducing sitting time pre vs. post<br>Systolic blood pressure: 122.6±7.9 vs. 116.7±12.3<br>Diastolic blood pressure: 78.8±6.8 vs. 75.3±8.3<br><br>Reducing sitting time pre vs. post<br>Systolic blood pressure: 127.3±10.4 vs. 122.6±11.8<br>Diastolic blood pressure: 82.9±5.3 vs. 78.9±6.9	Systolic blood pressure significantly decreased in all intervention groups. Diastolic blood pressure significantly decreased in reducing sitting time group. There were no significant changes in fasting glucose pre- to post-intervention or between groups.	Appropriate conclusions based on available data

Kucio et al, 2017[30]	Exercise pre vs. post median±SD.<br>Day time systolic blood pressure: 145.1±11.8 vs. 139.6±13.9<br>Daytime diastolic blood pressure: 85.4±6.2 vs. 82.5±6.9<br><br>Control pre vs. post median±SD.<br>Day time systolic blood pressure: 138.5±11.9 vs. 136.1±10.8<br>Daytime diastolic blood pressure: 79.8±5.8 vs. 79.0±5.1	No statistically significant differences were noted in median daily systolic blood pressure values between the groups both pre- and post-examination.	Appropriate conclusions based on available data
Labrunée et al, 2012[31]	Exercise pre vs. post mean±SD.<br>HOMA-IR: 26.8±75.4 vs. 14.9±20.6<br><br>Control pre vs. post mean±SD.<br>HOMA-IR: 23.1±53.4 vs. 18.1±30.4	The anthropometric and biological parameters did not show significant modification	Appropriate conclusions based on available data
Larson-Myer et al, 2009[32]	Exercise mean change ±SD.<br>Systolic blood pressure (mmHg) -1.66±2.44<br>Diastolic blood pressure (mmHg): -4.0±2.1<br><br>Control mean change ±SD.<br>Systolic blood pressure (mmHg) -2.75±1.65<br>Diastolic blood pressure (mmHg): -2.06±1.71	Diastolic blood pressure was significantly (P <0.02) improved versus baseline only in the caloric restriction+exercise group but not in the caloric restriction group. Systolic blood pressure was not changed by any of the treatments.	Significant benefit from intervention and small sample size.
Masuo et al, 2012[33]	Diet+Exercise pre vs. post mean±SD.<br>Systolic blood pressure (mmHg): 156±6 vs. 136±6<br>Diastolic blood pressure (mmHg): 98±5 vs. 81±7<br>HOMA-IR: 2.9±0.5 vs. 1.8±0.5<br><br>Control pre vs. post mean±SD.<br>Systolic blood pressure (mmHg): 154±4 vs. 141±5<br>Diastolic blood pressure (mmHg): 98±5 vs. 86±4<br>HOMA-IR: 2.8±0.3 vs. 2.3±0.4	The weight-loss protocol with a combination of Diet+Exercise had the strongest ameliorative effect on weight loss, being especially effective on blood pressure reduction, normalization of blood pressure, and insulin resistance.	Appropriate conclusions based on available data
Meckling et al, 2007[34]	Control diet pre vs. post<br>Systolic blood pressure (mmHg): 127±14 vs. 118±12<br>Diastolic blood pressure (mmHg): 81±11 vs. 75±14<br><br>Control diet+exercise pre vs. post<br>Systolic blood pressure (mmHg): 129±7 vs. 122±10<br>Diastolic blood pressure (mmHg): 82±8 vs. 77±9<br><br>High protein diet pre vs. post<br>Systolic blood pressure (mmHg): 128±19 vs. 119±13<br>Diastolic blood pressure (mmHg): 79±8 vs. 72±9<br><br>High protein diet+exercise pre vs. post<br>Systolic blood pressure (mmHg): 134±12 vs. 127±17<br>Diastolic blood pressure (mmHg): 82±7 vs. 78±10	A 4 intervention groups experienced improvements in several risk factors. All experienced a decrease in systolic blood pressure and most experienced a decrease in diastolic blood pressure as well. The magnitudes of these changes are particularly significant, because no subject was enrolled with a starting blood pressure greater than 140/90 mmHg. Thus, even normotensive individuals experienced decreased blood pressure, which is associated with decreased risk of CVD and related diseases.	Appropriate conclusions based on available data. The main outcome was not the additive effect of exercise. The additive effect of exercise in reducing blood pressure is not so evident.
Mendham et al, 2014[35]	Exercise pre vs. post mean±SD.<br>Systolic blood pressure (mmHg): 123.7±8.3 vs. 123.4±8.2<br>Diastolic blood pressure (mmHg): 79.9±6.9 vs. 78.4±8.6<br>HOMA-IR: 4.1±2.8 vs. 3.2±1.6<br><br>Control pre vs. post mean±SD.<br>Systolic blood pressure (mmHg): 128.8±15.7 vs. 122.1±10<br>Diastolic blood pressure (mmHg): 80.5±10.1 vs. 79.5±10.3	In conclusion, a 12-week exercise program within Indigenous Australian men shows improvements in metabolic, anthropometric and fitness variables.	Negligible effect for the selected outcomes. Negligible differences between intervention and controls.

	HOMA-IR: 3.9±2.2 vs. 4.9±3.4		
Mohr et al, 2014[36]	HIT pre vs. post **<br>Systolic blood pressure (mmHg): 138±4 vs. 132±3.9<br>Diastolic blood pressure (mmHg): 86±3 vs. 84±3<br><br>MICT pre vs. post **<br>Systolic blood pressure (mmHg): 142±3.9 vs. 138±4<br>Diastolic blood pressure (mmHg): 87±2 vs. 87±2<br><br>Control pre vs. post **<br>Systolic blood pressure (mmHg): 134.4 ±3.71 vs. 133.5±3<br>Diastolic blood pressure (mmHg): 81.5±2 vs. 82.5±2	In HIT, systolic blood decreased (p< 0.05) by 6±1mmHg (4±1%) during the 15 week intervention period, while the MICT group displayed a decrease (p < 0.05) of 4±1 mmHg (3±1%) in SBP. DBP was similar before and after intervention for HIT and MICT. No significant changes took place in neither SBP nor DBP in CON (0±0 and 0±0mmHg).	Appropriate conclusions based on available data
Oh S. et al, 2018[37]	Diet+Exercise. Mean change<br>Systolic blood pressure (mmHg) -3.3±8.3<br>Diastolic blood pressure (mmHg): -2.3±4.6<br>HOMA-IR: -0.9±1.3<br><br>Diet. Mean change<br>Systolic blood pressure (mmHg) -1.8±5.8<br>Diastolic blood pressure (mmHg): -1.4±8.6<br>HOMA-IR: 0.6±2.8<br><br>Exercise Mean change<br>Systolic blood pressure (mmHg) -5.8±8.8<br>Diastolic blood pressure (mmHg): -3.7±8.3<br>HOMA-IR: 0.0±0.8<br><br>Control Mean change<br>Systolic blood pressure (mmHg) 0.0±8.1<br>Diastolic blood pressure (mmHg): 1.8±5<br>HOMA-IR: 0.5±0.9	The most beneficial changes can be seen in diet+exercise for glucose and HOMA- IR levels.	Appropriate conclusions based on available data.
Plotnikoff et al, 2010[38]	Exercise pre vs. post.<br>Systolic blood pressure (mmHg) 125.1±12.7 vs. 122.4±8.6<br>Diastolic blood pressure (mmHg): 75.3±8.1 vs. 73.9±7.3<br><br>Control pre vs. post<br>Systolic blood pressure (mmHg) 127.1±12.6 vs. 126.7±10.7<br>Diastolic blood pressure (mmHg): 75.0±8.9 vs. 75.1±7.9	We found no improvements in blood pressure.	Appropriate conclusions based on available data.
Pugh et al, 2014[39]	Exercise mean ***<br>Systolic blood pressure (mmHg) -0.5 (-4.2,4.4)<br>Diastolic blood pressure (mmHg): -0.3 (-2.9, 2.6)<br>Intrahepatic fat (%): -8.4 (-12.5, -4.2)<br>HOMA-IR: -0.2 (-0.9- 0.5)<br><br>Control mean ***<br>Systolic blood pressure (mmHg) -2 (-7.1,4.3)<br>Diastolic blood pressure (mmHg): -3.1 (-5.6, -0.1)<br>Intrahepatic fat (%): -5 (-10.3, 0.2)<br>HOMA-IR: -0.2 (-1- 0.5)	There was no statistically significant difference in liver fat between exercise training and conventional care [Difference between groups -3.3% (95% CI -10.0, 3.4), p =0.18].	Small and not significant effect of exercise on intrahepatic fat. Small effect on fasting glucose.
Pourranjbar et al, 2018[40]	Exercise pre vs. post mean ±SD.<br>HOMA-IR: 3.52±0.11 vs. 2.33±0.09	The serum insulin resistance level in the experimental group was significantly lower than that in the control group (p=0.000). Results of this survey demonstrated that insulin resistance decreased	Appropriate conclusions based on available data.

	Control pre vs. post mean ±SD.<br>HOMA-IR: 3.88±1.3 vs. 3.87±1.2	significantly in obese women after a period of aerobic training, demonstrating the positive effects of aerobic exercise on improving insulin-dependent indices in obesity.	
Ryan et al, 2014[41]	Diet+Exercise pre vs. post **.<br>Systolic blood pressure (mmHg): 123±2 vs. 118±2<br>Diastolic blood pressure (mmHg): 69±1vs. 67±1<br>HOMA-IR: 2.9±0.3 vs. 2.3±0.2<br><br>Diet pre vs. post **.<br>Systolic blood pressure (mmHg): 119±1 vs. 116±2<br>Diastolic blood pressure (mmHg): 68±1vs. 65±1<br>HOMA-IR: 3.0±0.2 vs. 2.1±0.1	Systolic blood pressure (SBP) decreased (p=.003), and diastolic BP (DBP) tended to decrease (p= .08) after aerobic exercise+weight loss. SBP and diastolicBP decreased after weight loss (p=.04).	Appropriate conclusions based on available data.
Schroeder et al, 2018[42]	Aerobic Exercise change ***<br>Systolic blood pressure (mmHg): 0 (-4; 4)<br>Diastolic blood pressure (mmHg): -2 (-4; 0)<br><br>Resitance Exercise change ***<br>Systolic blood pressure (mmHg): -1 (-5; 3)<br>Diastolic blood pressure (mmHg): 0 (-2; 3)<br><br>Combined Exercise change ***<br>Systolic blood pressure (mmHg): 0 (-4; 4)<br>Diastolic blood pressure (mmHg): -4 (-6; 1)<br><br>Control Exercise change ***<br>Systolic blood pressure (mmHg): -1 (-5; 3)<br>Diastolic blood pressure (mmHg): 0 (-2; 0)	Our exercise intervention did not result in systolic blood pressure reductions. However, during the baseline visit 15 participants had a blood pressure <120/80. With normal baseline blood pressure, reductions with exercise are less likely to be detected.	Appropriate conclusions based on available data.
Shah et al, 2009[43]	Diet+exercise **.<br>Intrahepatic fat (%) −3.8 ± 1.2<br>Systolic blood pressure (mmHg): −24.4 ± 4.2<br>Diastolic blood pressure (mmHg): −7.8 ± 2.1<br>HOMA-IR: -1.2±0.4<br><br>Diet **.<br>Intrahepatic fat (%):−3.7 ± 1.1<br>Systolic blood pressure (mmHg): −19.9 ± 6.3<br>Diastolic blood pressure (mmHg): −3.0 ± 3.9<br>HOMA-IR: -1.5±0.6	Our findings demonstrate that weight loss induced by Diet or Diet+Exercise are equally effective in reducing intrahepatic fat content (by ~50%) and improving insulin sensitivity (by ~60%) in obese older adults. The results of our study show that exercise training did not have an additive effect on diet-induced weight loss in reducing intrahepatic fat. Diet+Exercise group had a greater reduction in systolic and diastolic blood pressure than the Diet group, suggesting that combining diet and exercise had an additive effect in improving blood pressure.	Appropriate conclusions based on available data.
Stensvold et al, 2010[44]	Aerobic interval training pre vs. post.<br>Systolic blood pressure (mmHg): 140.0±14.6 vs. 134.2±12<br>Diastolic blood pressure (mmHg): 89.0±8.1 vs. 85.0±5.5<br><br>Strength training pre vs. post<br>Systolic blood pressure (mmHg): 142.7±14.2 vs. 139.9±16.9<br>Diastolic blood pressure (mmHg): 90.7±10.9 vs. 88.9±11.2<br><br>Combined training pre vs. post<br>Systolic blood pressure (mmHg): 148.6±14.0 vs. 145.1±15.2<br>Diastolic blood pressure (mmHg): 89.0±7.1 vs. 89.8±6.4<br><br>Control pre vs. post<br>Systolic blood pressure (mmHg): 141.5±12.3 vs. 142.1±24.1<br>Diastolic blood pressure (mmHg): 90.1±7.1 vs. 89.5±13.6	Although not significantly, the Aerobic Interval Training group had an estimated reduction in systolic blood pressure of -5.5 mmHg (95% CI: -11.4 to 0.4) and in diastolic blood pressure of -4.1 mmHG (95% CI: -8.3 to 0.12; Table 2), which indicated a tendency toward an effect.	Appropriate conclusions based on available data.

Straznicky et al, 2012[45]	Diet+exercise mean change ±SD.<br>Fasting plasma glucose (mmol/L): −0.7 ± 0.4<br>Systolic blood pressure (mmHg): −10.3± 10.5<br>Diastolic blood pressure (mmHg): −3.6± 6.7<br>HOMA-IR: -0.84±1<br><br>Diet mean change ±SD.<br>Fasting plasma glucose (mmol/L): −0.6 ± 0.7<br>Systolic blood pressure (mmHg): −10.5± 9.9<br>Diastolic blood pressure (mmHg): −3.2± 6.0<br>HOMA-IR: -1.66±1.44	Co-intervention with moderate-intensity aerobic exercise training facilitated greater reduction in central adiposity measures (waist circumference and trunk fat mass) and improved maximal oxygen consumption by 19%, but did not translate to incremental benefits on liver enzyme concentrations or other metabolic (insulin resistance, dyslipidemia) and cardiovascular parameters (blood pressure, resting SNS activity), beyond that attained by WL alone.	None additive effect of exercise
Sullivan et al, 2012[46]	Exercise pre vs. post **<br>Intrahepatic fat (%): 20.36±4.18 vs. 17.56±2.73<br><br>Control pre vs. post **<br>Intrahepatic fat (%): 21.41±8.88 vs. 24.08±9.38	Our data demonstrate that this moderate intensity exercise program causes a small decrease in intrahepatic fat content, even when body weight and total body fat mass are maintained.	Small sample size. Small decrease in intrahepatic TG.
Swift et al, 2012[47]	Exercise 8KKW mean change (95%CI).<br>Systolic blood pressure (mmHg): −2.4 (-2.5, -1.29)<br>Diastolic blood pressure (mmHg): −0.38 (-1.86, 1.09)<br><br>Exercise 12KKW mean change (95%CI).<br>Systolic blood pressure (mmHg): −3.5 (-5.9, -1.16)<br>Diastolic blood pressure (mmHg): −0.20 (-1.59, 1.19)<br><br>Control mean change (95%CI).<br>Systolic blood pressure (mmHg): −1.59 (-3.9, -0.79)<br>Diastolic blood pressure (mmHg): −0.48 (-1.89, 0.93)	Following exercise training, there was no significant change in resting systolic blood pressure in the 8 (−2.4 mmHg, CI: −1.29, 2.50), and 12 kcal/kg/week groups (−3.5, CI: −5.9, −1.16) compared to control (−1.59 mmHg, CI: −3.9, 0.79). Similarly, there was no significant change in resting diastolic blood pressure in the 8 (−0.38 mmHG, CI: −1.86, 1.09) and 12 (−0.20, CI: −1.59, 1.19) kcal/kg/week groups following exercise training in compared to control (−0.48, CI: −1.89, 0.93). Analyses for trend between dose of exercise and resting blood pressure were not significant (p >0.05).	Appropriate conclusions based on available data.
Taghian et al, 2014[48]	Exercise pre vs. post<br>HOMA-IR: 3.76±1.79 vs. 2.57±0.59<br><br>Control pre vs. post<br>HOMA-IR: 1.38±0.27 vs. 1.23±0.19	Significant difference in insulin sensitivity	Appropriate conclusions based on available data.
Tjønna et al, 2018[49]	Continuous moderate pre vs. post **<br>Systolic blood pressure (mmHg): 131±6 vs. 121±5<br>Diastolic blood pressure (mmHg): 88±4 vs. 82±5<br><br>Aerobic interval training pre vs. post **.<br>Systolic blood pressure (mmHg): 144±5 vs. 135±5<br>Diastolic blood pressure (mmHg): 95±3 vs. 89±3<br><br>Control pre vs. post **<br>Systolic blood pressure (mmHg): 146±6 vs. 141±5<br>Diastolic blood pressure (mmHg): 95±5 vs. 96±4	Both aerobic interval training and continuous moderate exercise decreased systolic and diastolic blood pressures by 10 mm Hg (both P<0.05) and 6mm Hg (AIT, P<0.05; CME, P=0.24), respectively.	Appropriate conclusions based on available data.
Waib et al, 2011[50]	Exercise pre vs. post ***.<br>HOMA-IR: 2.7 (1.7-3.7) vs. 2.0 (1.6-2.5)<br>Daytime Systolic blood pressure (mmHg): 145.3 (142.1-148.5) vs. 145.0 (142.0-148.0)<br>Daytime Diastolic blood pressure (mmHg): 93.7 (91.2-96.2) vs. 93.0 (90.0-96.0)<br><br>Exercise pre vs. post***	No significant changes in blood pressure occurred after the 3-month period in either group. In the aerobic exercise group, HOMA-IR significantly decreased by 25%, whereas no change was observed in the control group.	Appropriate conclusions based on available data.

	HOMA-IR: 1.6 (1.2-1.9) vs. 2.0 (1.4-2.6)<br>Daytime Systolic blood pressure (mmHg): 144.0 (139.8-148.3) vs. 144.0 (139.2-148.9)<br>Daytime Diastolic blood pressure (mmHg): 91.6 (87.9-95.2) vs. 91.2 (86.7-95.7).		
Winding et al, 2017[51]	Endurance pre vs. post.<br>Systolic blood pressure (mmHg): 134±17 vs. 133±22<br>Diastolic blood pressure (mmHg): 82±7 vs. 79.±9<br>HOMA-IR: 1.28±0.56 vs. 1.58±0.72<br><br>HIIT pre vs. post.<br>Systolic blood pressure (mmHg): 134±17 vs. 133±22<br>Diastolic blood pressure (mmHg): 85±5 vs. 84±5<br>HOMA-IR: 2.38±2.24 vs. 1.79±1.47<br><br>Control pre vs. post.<br>Systolic blood pressure (mmHg): 139±7 vs. 143±9<br>Diastolic blood pressure (mmHg): 87±7 vs. 85±5<br>HOMA-IR: 2.18±1.32 vs. 2.18±1.16	HIIT lowered HOMA- IR (P<0.05)	Appropriate conclusions based on available data.
Winn NC et al, 2018[52]	MICT<br>Intrahepatic fat%: −20.1 ± 6.6<br><br>HIIT<br>Intrahepatic fat%: −37.0 ± 12.4<br><br>Control<br>Intrahepatic fat %: 17.3 ± 14.5	The most profound finding of the present investigation was that 4 weeks of energy-matched HIIT and MICT caused marked reductions in intrahepatic fat content without clinically significant changes in body mass, abdominal adiposity, liver enzyme levels, or biomarkers of hepatic function.	Appropriate conclusions based on available data.
Zelber-Sagi et al, 2014[53]	Exercise mean change<br>HOMA-IR: 0.37±2.4<br><br>Control mean change.<br>HOMA-IR: -0.24±1.75	RT had no significant impact on serum glucose, insulin, glycosylated haemoglobin.	Appropriate conclusions based on available data.
Zhang HJ et al, 2016[54]	Moderate exercise ***<br>Intrahepatic fat %: − 6.3 (-7.9, -4.8)<br><br>Vigorous exercise ***<br>Intrahepatic fat: − 7.2 (- 8.7, -5.6)<br><br>Control ***<br>Intrahepatic fat: − 2.2 (-3.7, -0.7)	In Chinese adults with abdominal obesity and NAFLD, intrahepatic triglyceride content was significantly reduced by 5.0% in the vigorous exercise group and 4.2 %in the moderate exercise group compared with a control group during 6 months. The change in intrahepatic triglyceride content was not significantly different between the vigorous and moderate exercise groups.	Appropriate conclusions based on available data

Abbreviations: 1-RM: one repetition maximum; BMI: body mass index; HIIT: high intensity interval training; HR: heart rate; HRR: heart rate reserve; HTN: arterial hypertension; MetS: metabolic syndrome; MICT: moderate intensity continuous training; NAFLD: non alcoholic fatty liver disease; NASH: non alcoholic steatohepatitis; RM: repetition maximum; T2DM: type 2 diabetes; y: years. Unless otherwise specified, values are presented as mean±SD. * median (IQR). ** mean±SEM. *** mean (95%CI).

References

1. Abdelaal AAM, Mohamad MA. Obesity indices and haemodynamic response to exercise in obese diabetic hypertensive patients: Randomized controlled trial. *Obes Res Clin Pract*. 2015;9(5):475-486. doi:10.1016/j.orcp.2014.11.001
2. Abdelbasset WK, Tantawy SA, Kamel DM, Alqahtani BA, Soliman GS. A randomized controlled trial on the effectiveness of 8-week high-intensity interval exercise on intrahepatic triglycerides, visceral lipids,

and health-related quality of life in diabetic obese patients with nonalcoholic fatty liver disease. *Medicine (Baltimore)*. 2019;98(12).
3. Abdelbasset WK, Elsayed SH, Nambi G, et al. Effect of Moderate-Intensity Aerobic Exercise on Hepatic Fat Content and Visceral Lipids in Hepatic Patients with Diabesity: A Single-Blinded Randomised Controlled Trial. *Evidence-based Complement Altern Med*. 2020;2020. doi:10.1155/2020/1923575
4. Andersen TR, Schmidt JF, Thomassen M, et al. A preliminary study: Effects of football training on glucose control, body composition, and performance in men with type 2 diabetes. *Scand J Med Sci Sport*. 2014;24(SUPPL.1):43-56. doi:10.1111/sms.12259
5. Balducci S, Zanuso S, Cardelli P, et al. Supervised exercise training counterbalances the adverse effects of insulin therapy in overweight/obese subjects with type 2 diabetes. *Diabetes Care*. 2012;35(1):39-41. doi:10.2337/dc11-1450
6. Bouchonville M, Armamento-Villareal R, Shah K, et al. Weight loss, exercise or both and cardiometabolic risk factors in obese older adults: Results of a randomized controlled trial. *Int J Obes*. 2014;38(3):423-431. doi:10.1038/ijo.2013.122
7. Cao L, Jiang Y, Li Q, Wang J, Tan S. Exercise training at maximal fat oxidation intensity for overweight or obese older women: A randomized study. *J Sport Sci Med*. 2019;18(3):413-418.
8. Croymans DM, Krell SL, Oh CS, et al. Effects of resistance training on central blood pressure in obese young men. *J Hum Hypertens*. 2014;28(3):157-164. doi:10.1038/jhh.2013.81
9. Cuthbertson DJ, Shojaee-Moradie F, Sprung VS, et al. Dissociation between exercise-induced reduction in liver fat and changes in hepatic and peripheral glucose homoeostasis in obese patients with non-alcoholic fatty liver disease. *Clin Sci*. 2016;130(2):93-104. doi:10.1042/CS20150447
10. Fenkci S, Sarsan A, Rota S, Ardic F. Effects of resistance or aerobic exercises on metabolic parameters in obese women who are not on a diet. *Adv Ther*. 2006;23(3):404-413. doi:10.1007/BF02850161
11. Figueroa A, Gil R, Wong A, et al. Whole-body vibration training reduces arterial stiffness, blood pressure and sympathovagal balance in young overweight/obese women. *Hypertens Res*. 2012;35(6):667-672. doi:10.1038/hr.2012.15
12. Figueroa A, Kalfon R, Wong A. Whole-body vibration training decreases ankle systolic blood pressure and leg arterial stiffness in obese postmenopausal women with high blood pressure. *Menopause*. 2015;22(4):423-427. doi:10.1097/GME.0000000000000332
13. Fritz T, Caidahl K, Lundstrom P, et al. Effects of Nordic walking on cardiovascular risk factors in overweight individuals with type 2 diabetes, impaired or normal glucose tolerance. *Diabetes Metab Res Rev*. 2013;29:25–32. doi:10.1002/dmrr
14. García-Unciti M, Izquierdo M, Idoate F, et al. Weight-loss diet alone or combined with progressive resistance training induces changes in association between the cardiometabolic risk profile and abdominal fat depots. *Ann Nutr Metab*. 2012;61(4):296-304. doi:10.1159/000342467
15. Goodpaster BH, DeLany JP, Otto AD, et al. Effects of diet and physical activity interventions on weight loss and cardiometabolic risk factors in severely obese adults: A randomized trial. *JAMA - J Am Med Assoc*. 2010;304(16):1795-1802. doi:10.1001/jama.2010.1505
16. Gorostegi-Anduaga I, Corres P, MartinezAguirre-Betolaza A, et al. Effects of different aerobic exercise programmes with nutritional intervention in sedentary adults with overweight/obesity and hypertension: EXERDIET-HTA study. *Eur J Prev Cardiol*. 2018;25(4):343-353. doi:10.1177/2047487317749956
17. Hallsworth K, Fattakhova G, Hollingsworth KG, et al. Resistance exercise reduces liver fat and its mediators in non-alcoholic fatty liver disease independent of weight loss. *Gut*. 2011;60(9):1278-1283. doi:10.1136/gut.2011.242073
18. Hallsworth K, Thoma C, Hollingsworth KG, et al. Modified high-intensity interval training reduces liver fat and improves cardiac function in non-alcoholic fatty liver disease: A randomized controlled trial. *Clin Sci*. 2015;129(12):1097-1105. doi:10.1042/CS20150308
19. Heydari M, Boutcher YN, Boutcher SH. The effects of high-intensity intermittent exercise training on cardiovascular response to mental and physical challenge. *Int J Psychophysiol*. 2013;87(2):141-146. doi:10.1016/j.ijpsycho.2012.11.013
20. Hinderliter AL, Sherwood A, Craighead LW, et al. The long-term effects of lifestyle change on blood pressure: One-year follow-up of the ENCORE study. *Am J Hypertens*. 2014;27(5):734-741. doi:10.1093/ajh/hpt183
21. Ho SS, Radavelli-Bagatini S, Dhaliwal SS, Hills AP, Pal S. Resistance, aerobic, and combination training on vascular function in overweight and obese adults. *J Clin Hypertens*. 2012;14(12):848-854. doi:10.1111/j.1751-7176.2012.00700.x
22. Houghton D, Thoma C, Hallsworth K, et al. Exercise Reduces Liver Lipids and Visceral Adiposity in Patients With Nonalcoholic Steatohepatitis in a Randomized Controlled Trial. *Clin Gastroenterol Hepatol*. 2017;15(1):96-102.e3. doi:10.1016/j.cgh.2016.07.031
23. Johnson NA, Sachinwalla T, Walton DW, et al. Aerobic exercise training reduces hepatic and visceral lipids in obese individuals without weight loss. *Hepatology*. 2009;50(4):1105-1112. doi:10.1002/hep.23129
24. Kadoglou NPE, Iliadis F, Sailer N, et al. Exercise training ameliorates the effects of rosiglitazone on traditional and novel cardiovascular risk factors in patients with type 2 diabetes mellitus. *Metabolism*. 2010;59(4):599-607. doi:10.1016/j.metabol.2009.09.002
25. Keating SE, Hackett DA, George J, Johnson NA. Exercise and non-alcoholic fatty liver disease: A systematic review and meta-analysis. *J Hepatol*. 2012;57(1):157-166. doi:10.1016/j.jhep.2012.02.023
26. Kim JW, Kim DY. Effects of aerobic exercise training on serum sex hormone binding globulin, body fat index, and metabolic syndrome factors in obese postmenopausal women. *Metab Syndr Relat Disord*. 2012;10(6):452-457. doi:10.1089/met.2012.0036
27. Kim YS, Nam JS, Yeo DW, Kim KR, Suh SH, Ahn CW. The effects of aerobic exercise training on serum osteocalcin, adipocytokines and insulin resistance on obese young males. *Clin Endocrinol (Oxf)*. 2015;82(5):686-694. doi:10.1111/cen.12601

28. Kolahdouzi S, Baghadam M, Kani-Golzar FA, et al. Progressive circuit resistance training improves inflammatory biomarkers and insulin resistance in obese men. *Physiol Behav*. 2019;205:15-21. doi:10.1016/j.physbeh.2018.11.033
29. Keadle SK, Lyden K, Staudenmayer J, et al. The independent and combined effects of exercise training and reducing sedentary behavior on cardiometabolic risk factors. *Appl Physiol Nutr Metab*. 2014;39(7):770-780. doi:10.1139/apnm-2013-0379
30. Kucio C, Narloch D, Kucio E, Kurek J. The application of nordic walking in the treatment hypertension and obesity. *Fam Med Prim Care Rev*. 2017;19(2):144-148. doi:10.5114/fmpcr.2017.67870
31. Labrunée M, Antoine D, Vergès B, Robin I, Casillas JM, Gremeaux V. Effects of a home-based rehabilitation program in obese type 2 diabetics. *Ann Phys Rehabil Med*. 2012;55(6):415-429. doi:10.1016/j.rehab.2012.06.001
32. Larson-Meyer DE, Redman L, Heilbronn LK, Martin CK, Ravussin E. Caloric restriction with or without exercise: The fitness versus fatness debate. *Med Sci Sports Exerc*. 2010;42(1):152-159. doi:10.1249/MSS.0b013e3181ad7f17
33. Masuo K, Rakugi H, Ogihara T, Lambert GW. Different mechanisms in weight loss-induced blood pressure reduction between a calorie-restricted diet and exercise. *Hypertens Res*. 2012;35(1):41-47. doi:10.1038/hr.2011.134
34. Meckling KA, Sherfey R. A randomized trial of a hypocaloric high-protein diet, with and without exercise, on weight loss, fitness, and markers of the Metabolic Syndrome in overweight and obese women. *Appl Physiol Nutr Metab*. 2007;32(4):743-752. doi:10.1139/H07-059
35. Mendham AE, Duffield R, Marino F, Coutts AJ. A 12-week sports-based exercise programme for inactive Indigenous Australian men improved clinical risk factors associated with type 2 diabetes mellitus. *J Sci Med Sport*. 2015;18(4):438-443. doi:10.1016/j.jsams.2014.06.013
36. Mohr M, Nordsborg NB, Lindenskov A, et al. High-Intensity intermittent swimming improves cardiovascular health status for women with mild hypertension. *Biomed Res Int*. 2014;2014. doi:10.1155/2014/728289
37. Oh M, Kim S, An KY, et al. Effects of alternate day calorie restriction and exercise on cardio-metabolic risk factors in overweight and obese adults: An exploratory randomized controlled study. *BMC Public Health*. 2018;18(1):1-10. doi:10.1186/s12889-018-6009-1
38. Plotnikoff RC, Eves N, Jung M, Sigal RJ, Padwal R, Karunamuni N. Multicomponent, home-based resistance training for obese adults with type 2 diabetes: A randomized controlled trial. *Int J Obes*. 2010;34(12):1733-1741. doi:10.1038/ijo.2010.109
39. Pugh CJA, Sprung VS, Kemp GJ, et al. Exercise training reverses endothelial dysfunction in nonalcoholic fatty liver disease. *Am J Physiol - Hear Circ Physiol*. 2014;307(9):H1298-H1306. doi:10.1152/ajpheart.00306.2014
40. Pourranjbar M, Arabnejad N, Naderipour K, Rafie F. Effects of Aerobic Exercises on Serum Levels of Myonectin and Insulin Resistance in Obese and Overweight Women. *J Med Life*. 2018;11(4):381-386. doi:10.25122/jml-2018-0033
41. Ryan AS, Ge S, Blumenthal JB, Serra MC, Prior SJ, Goldberg AP. Aerobic exercise and weight loss reduce vascular markers of inflammation and improve insulin sensitivity in obese women. *J Am Geriatr Soc*. 2014;62(4):607-614. doi:10.1111/jgs.12749
42. Schroeder EC, Franke WD, Sharp RL, Lee D chul. Comparative effectiveness of aerobic, resistance, and combined training on cardiovascular disease risk factors: A randomized controlled trial. *PLoS One*. 2019;14(1):1-14. doi:10.1371/journal.pone.0210292
43. Shah K, Stufflebam A, Hilton TN, Sinacore DR, Klein S, Villareal DT. Diet and exercise interventions reduce intrahepatic fat content and improve insulin sensitivity in obese older adults. *Obesity*. 2009;17(12):2162-2168. doi:10.1038/oby.2009.126
44. Stensvold D, Tjønna AE, Skaug EA, et al. Strength training versus aerobic interval training to modify risk factors of metabolic syndrome. *J Appl Physiol*. 2010;108(4):804-810. doi:10.1152/japplphysiol.00996.2009
45. Straznicky1, E.A.Lambert1, 6, M. T. Grima1, N. Eikelis2, P.J.Nestel3, T. Dawood1, M.P. Schlaich2, 5, K. Masuo1, R.Chopra1, C.I.Sari1, J.B.Dixon4, 6, A. J. Tilbrook6 & G. W. Lambert1 5. The effects of dietary weight loss with or without exercise training on liver enzymes in obese metabolic syndrome subjects. *Diabetes, Obes Metab*. 2012;14:139-148.
46. Sullivan S, Kirk EP, Mittendorfer B, Patterson BW, Klein S. Randomized trial of exercise effect on intrahepatic triglyceride content and lipid kinetics in nonalcoholic fatty liver disease. *Hepatology*. 2012;55(6):1738-1745. doi:10.1002/hep.25548
47. Swift, Damon L. Ph.D.1, Conrad P. Earnest, Ph.D.2, Peter T. Katzmarzyk, Ph.D3, Tuomo Rankinen, Ph.D.4, Steven N. Blair, P.E.D.5, and Timothy S. Church, M.D. PD. The Effect of Different Doses of Aerobic Exercise Training on Exercise Blood Pressure in Overweight and Obese Postmenopausal Women. *Menopause*. 2012;19(5):503-509. doi:10.1038/jid.2014.371
48. Taghian F, Zolfaghari M, Hedayati M. Effects of aerobic exercise on serum retinol binding protein4, insulin resistance and blood lipids in obese women. *Iran J Public Health*. 2014;43(5):658-665.
49. Tjønna AE, Lee SJ, Rognmo Ø, et al. Aerobic interval training versus continuous moderate exercise as a treatment for the metabolic syndrome: A pilot study. *Circulation*. 2008;118(4):346-354. doi:10.1161/CIRCULATIONAHA.108.772822
50. Waib PH, Gonçalves MI, Barrile SR. Improvements in Insulin Sensitivity and Muscle Blood Flow in Aerobic-Trained Overweight-Obese Hypertensive Patients Are Not Associated With Ambulatory Blood Pressure. *J Clin Hypertens*. 2011;13(2):89-96. doi:10.1111/j.1751-7176.2010.00393.x
51. Winding KM, Munch GW, Iepsen UW. The effect of low-volume high-intensity interval training. *Diabetes, Obes Metab*. 2018;20(6):1-27.
52. Winn NC, Liu Y, Rector RS, Parks EJ, Ibdah JA, Kanaley JA. Energy-matched moderate and high intensity exercise training improves nonalcoholic fatty liver disease risk independent of changes in body mass or abdominal adiposity — A randomized trial. *Metabolism*. 2018;78:128-140. doi:10.1016/j.metabol.2017.08.012

53. Zelber-Sagi S, Buch A, Yeshua H, et al. Effect of resistance training on non-alcoholic fatty-liver disease a randomized-clinical trial. *World J Gastroenterol*. 2014;20(15):4382-4392. doi:10.3748/wjg.v20.i15.4382
54. Zhang HJ, He J, Pan LL, et al. Effects of moderate and vigorous exercise on nonalcoholic fatty liver disease: A randomized clinical trial. *JAMA Intern Med*. 2016;176(8):1074-1082. doi:10.1001/jamainternmed.2016.3202
